# Highly electrophilic, *gem-* and *spiro-*activated trichloromethylnitrocyclopropanes: synthesis and structure

**DOI:** 10.3762/bjoc.22.5

**Published:** 2026-01-14

**Authors:** Ilia A Pilipenko, Mikhail V Grigoriev, Olga Yu Ozerova, Igor A Litvinov, Darya V Spiridonova, Aleksander V Vasilyev, Sergey V Makarenko

**Affiliations:** 1 Department of Chemistry, Saint-Petersburg State Forest Technical University, Saint-Petersburg 194021, Russia; 2 Department of Organic Chemistry, Herzen State Pedagogical University of Russia, Saint-Petersburg 191186, Russia; 3 A. E. Arbuzov Institute of Organic and Physical Chemistry, FRC Kazan Scientific Center of the Russian Academy of Sciences, Kazan 420088, Russia; 4 Department of Organic Chemistry, Institute of Chemistry, Saint-Petersburg State University, Saint-Petersburg 199034, Russia

**Keywords:** bromonitropropene, CH-acids, cyclopropanes, nitrocyclopropanes, trichloromethyl group, X-ray

## Abstract

New highly electrophilic *gem*- and *spiro*-activated trichloromethylnitrocyclopropanes were obtained by the Michael-initiated ring closure (MIRC) reaction of 1-bromo-1-nitro-3,3,3-trichloropropene with linear and cyclic CH-acids catalyzed by bases. Conditions for obtaining the target cyclopropanes were optimized. The process is characterized by high diastereoselectivity and allows obtaining cyclopropanes with *trans*-configuration of -NO_2_ and -CCl_3_ groups. Monocyclic (based on malonic acid dinitrile, methyl cyanoacetate, ethyl cyanoacetate, benzoylacetonitrile), spirocarbo- (based on 1,3-indanedione) and spiroheterocyclic (based on Meldrum's acid, dimethylbarbituric acid, 3-methyl-1-phenyl-5-pyrazolone) cyclopropane structures were isolated and characterized.

## Introduction

**Figure 1:**
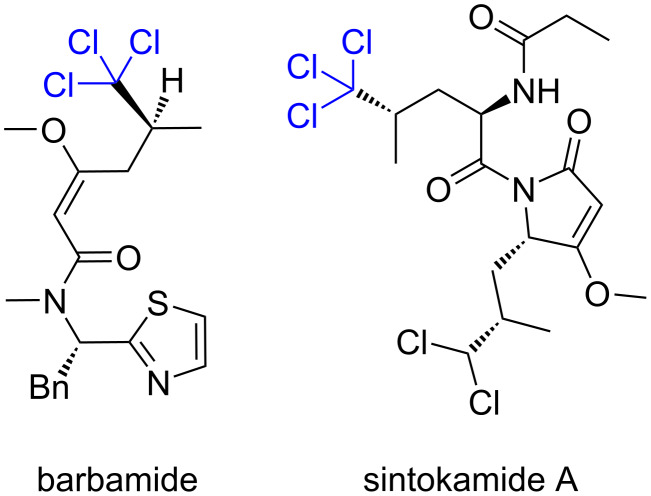
Two natural trichloromethyl-containing compounds.

Trichloromethyl groups containing compounds are widely used in the organic synthesis of practically significant substances [1–2]. Based on them, methods have been developed for the synthesis of hard-to-access 5-aminoisoxazoles [3], α- and γ-heterosubstituted unsaturated carboxylic acids [4]. The trichloromethyl group is a convenient precursor of the carboxylic function, which determines their use in the synthesis of α-amino acids [5]. A number of natural trichloromethyl-containing compounds are metabolites of symbionts of marine sponges – cyanobacteria [6–8] – and have biologically active properties. Thus, barbamide exhibits molluscicidal activity [7], and sintokamide A is active against prostate cancer [8] (Figure 1).

As derivatives of strained and unique structure and properties [9–10] cyclopropanes are of interest for entering into various transformations along the path of ring opening or expanding [11–13]. Thus, known trichloromethyl-containing cyclopropanes can serve as precursors for hard-to-access halogenated β,γ- and γ,δ-unsaturated esters [14–15].

Nitrocyclopropanes are known as highly electrophilic substrates and can form a carbanion stabilized by the nitro group upon breaking the C–C bond [16–17]. Substituted nitrocyclopropanes in reactions with various nucleophiles form linear precursors for the synthesis of γ-substituted α-aminobutyric acids [18–19], cyclic nitropyrrolines [20] and isoxazoline *N*-oxides [18]. The nitrocyclopropane fragment is a part of the hormaomycin antibiotic [21], and also acts as a precursor for the synthesis of the aminocyclopropane [22] moiety, which is a component of some drugs, such as ciprofloxacin [23] and belactosin A [24]. Thus, the construction of cyclopropanes containing vicinal nitro- and trichloromethyl groups seems attractive for both theoretical chemistry and the synthesis of 2-aminocyclopropanecarboxylic acids, of which representatives have biologically active properties against kynurenine-3-monooxygenase [25] and GABA receptors [26]. Such aminocyclopropane derivatives can be classified as donor–acceptor cyclopropanes (DACs), the chemistry of which has been studied particularly intensively in recent years [27–30].

**Scheme 1:**
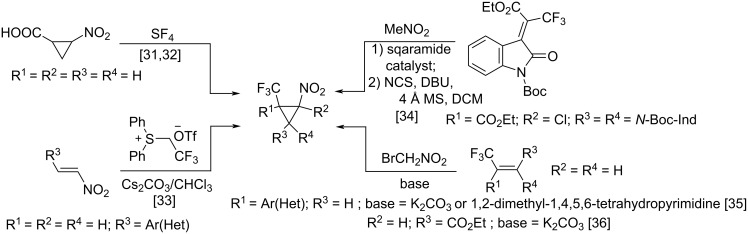
Approaches to the synthesis of *vic*-trifluoromethylnitrocyclopropanes.

It is worth noting that vicinal trichloromethylnitrocyclopropanes have not been previously described. Their analogues, *vic*-trifluoromethylnitrocyclopropanes, have been obtained by several approaches: forming of the CF_3_-group in a nitrocyclopropane (reaction of 2-nitrocyclopropanecarboxylic acid with sulfur tetrafluoride [31–32]), cyclopropane formation from a nitroethene substrate and a CF_3_-containing reagent (Corey–Chaykovsky reaction [33]), as well as reactions involving a CF_3_-containing substrate and a nitromethylene component (the tandem reaction of trifluoromethyl-substituted alkenes with nitromethane [34] or bromonitromethane derivatives [35–36]) (Scheme 1).

At the same time, one of the methods of synthesis of vicinally substituted nitrocyclopropanes is the Michael-initiated ring closure (MIRC) reaction of *gem*-halonitroalkenes and CH-acids [37–38]. Thus, 2-nitrocyclopropanecarboxylates were obtained based on the tandem reactions of cyclic CH-acids with alkyl 3-bromo-3-nitroacrylates [39]. Despite the structural proximity and high activity of 1-bromo-1-nitro-3,3,3-trichloropropene (**1**) [40] in reactions with nucleophiles, including those following the formation of cyclic products in tandem transformations [41–44], methods for obtaining cyclopropane structures based on it are not known. In this way, it seemed desirable to synthesize vicinal trichloromethylnitrocyclopropanes based on the well-known 1-bromo-1-nitro-3,3,3-trichloropropene (**1**).

## Results and Discussion

It turned out that the synthesis of the target trichloromethylnitrocyclopropane **2** based on the reaction of 1-bromo-1-nitro-3,3,3-trichloropropene (**1**) with malononitrile under conditions similar to those described earlier [39] results in its formation in 18% of yield (Table 1, method A). Optimization of the process by using various bases and solvents showed that the best yield of cyclopropane **2** (64%) was obtained in a tetrahydrofuran (THF) solution in the presence of triethylamine (Table 1, method E).

**Table 1:** Reaction of compound **1** with malononitrile leading to cyclopropane **2** under various conditions.

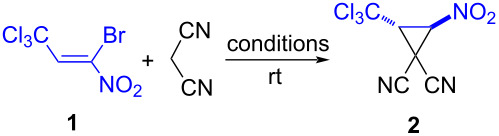				

Method	Reaction conditions			Yield of **2**, %

	Solvent	Base	Time, min	

A	MeOH	AcOK	15	18
B	MeOH	MeONa	10	oligomers
C	THF	AcOK	120	20
D	THF	DBU	60	oligomers
E	THF	Et_3_N	60	64

**Scheme 2:**
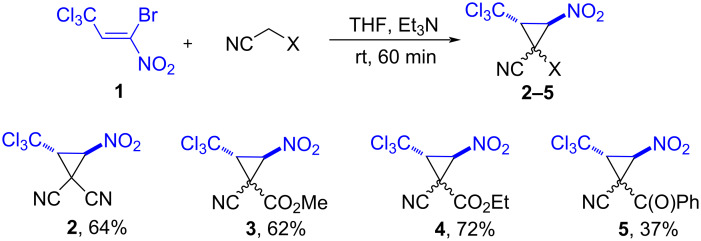
Synthesis of monocyclic trichloromethylnitrocyclopropanes **2**–**5**.

The use of method E in the reaction of 1-bromo-1-nitro-3,3,3-trichloropropene (**1**) with methyl cyanoacetate, ethyl cyanoacetate or benzoylacetonitrile makes it possible to obtain cyclopropanes **3**–**5** in the yields up to 72% (Scheme 2). They are formed as single diastereomers (according to the ^1^H NMR spectra of the crude compounds).

**Scheme 3:**
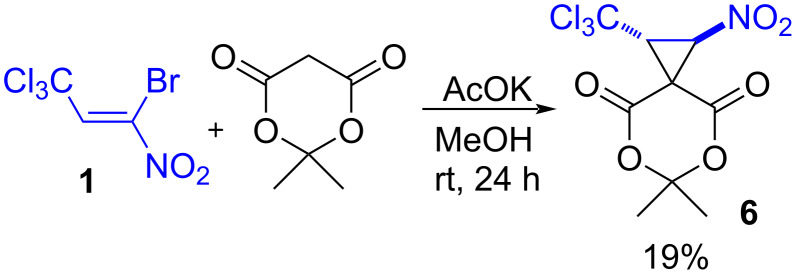
Synthesis of spiro-fused trichloromethylnitrocyclopropane **6**.

The synthesis of spiro-fused trichloromethylnitrocyclopropane **6** based on the reaction of *gem*-bromonitroalkene **1** and Meldrum’s acid under the conditions of method E is completed by oiling-out the reaction mixture. At the same time, using the conditions of method A (bromonitroalkene/CH-acid/base = 1:1:1.5 ratio) for 24 hours makes it possible to isolate the target cyclopropane **6** with a yield of 19% (Scheme 3).

**Scheme 4:**
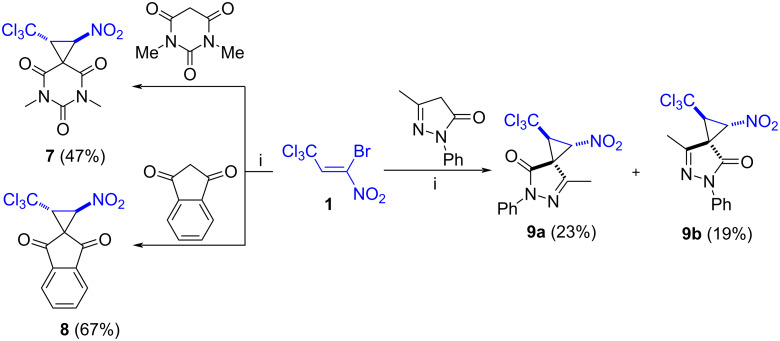
Synthesis of spiro-fused trichloromethylnitrocyclopropanes **7**–**9**. i: 1.5 AcOK, MeOH, rt, 3 h.

The use of method A, but with a longer reaction time (3 hours), analogous to literature procedures [39], proved to be more successful in reactions with other cyclic CH-acids (dimethylbarbituric acid, 1,3-indanedione, 3-methyl-1-phenyl-5-pyrazolone). The spiro-fused *vic-*trichloromethylnitrocyclopropanes **7**–**9** were obtained in 42–67% yields (Scheme 4).

**Scheme 5:**
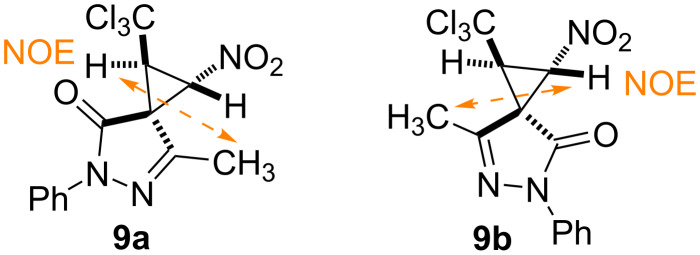
Main NOE correlations in **9a**, **9b**.

The pyrazolone-conjugated trichloromethyl-containing nitrocyclopropane **9** is formed as a mixture of two diastereomers **9a** and **9b** (1.3:1 dr, according to the ^1^H NMR spectrum) due to the axial chirality of this molecule. The mixture was easily separated by silica gel column chromatography. Each of the isomers is characterized by the *trans-*configuration of the nitro- and trichloromethyl groups in the cyclopropane ring (^3^*J*_H(1)H(2)_ = 6.6–6.8 Hz) which agrees with the literature data for structurally similar compounds [37–38,44]. According to ^1^H-^1^H NOESY spectroscopy data, NOE correlation of C^1^H (δ_H_ = 4.44 ppm)/CH_3_ (pyrazolone) (δ_H_ = 2.15 ppm) protons is observed in the major diastereomer **9a**, and C^2^H (δ_H_ = 5.54 ppm)/CH_3_ (pyrazolone) (δ_H_ = 2.15 ppm) in the minor isomer **9b** (Scheme 5). Thus, the relative configurations of the stereocenters in these molecules can be defined as 1*SR*,2*RS*,3*SR* (**9a**) and 1*SR*,2*RS*,3*RS* (**9b**).

Cyclopropanes **2**–**8** are formed as single diastereomers. The vicinal spin–spin coupling constants of C^1^H–C^2^H protons of the cyclopropane ring (^3^*J*_H(1)H(2)_ = 5.7–7.5 Hz) indicate their transoid arrangement [38–39,45]. This makes it possible to assign 1*SR*,2*RS* configurations to the stereocenters.

**Scheme 6:**
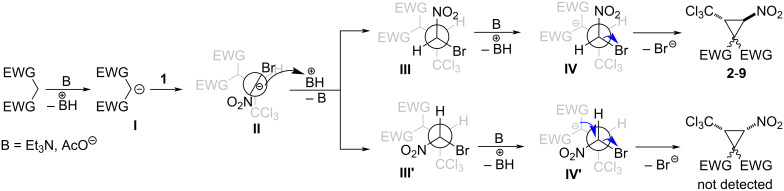
Proposed mechanism of the formation of trichloromethylnitrocyclopropanes.

The proposed mechanism for this transformation is depicted in Scheme 6. Michael addition of the CH-acid anion **I** to the bromonitroalkene afford the intermediate anion **II**, followed by tautomerization and formation of anion **IV**, which undergoes intramolecular nucleophilic substitution of the bromide along the *C*-alkylation pathway [37–39] (Scheme 6).

The *trans*-configuration of the methine protons of the cyclopropane ring is probably a consequence of the formation of a sterically hindered carbanion **II** in a conformation with an *anti*-periplanar position of the bulky substituents – bromine and a trichloromethyl group (Scheme 6). Due to the steric reasons, the anion in this conformation is selectively protonated by BH^+^ from the side opposite to the –CH(EWG)_2_. Thus, only diastereomer **III** is formed. Deprotonation of this intermediate leads to carbanion **IV**. For further attack by the carbanion center to the carbon atom bonded to bromine, the –C(EWG)_2_ moiety must hold an *anti*-periplanar position relative to the bromine. The cyclization step from this conformation leads to *trans*-cyclopropanes.

**Figure 2:**
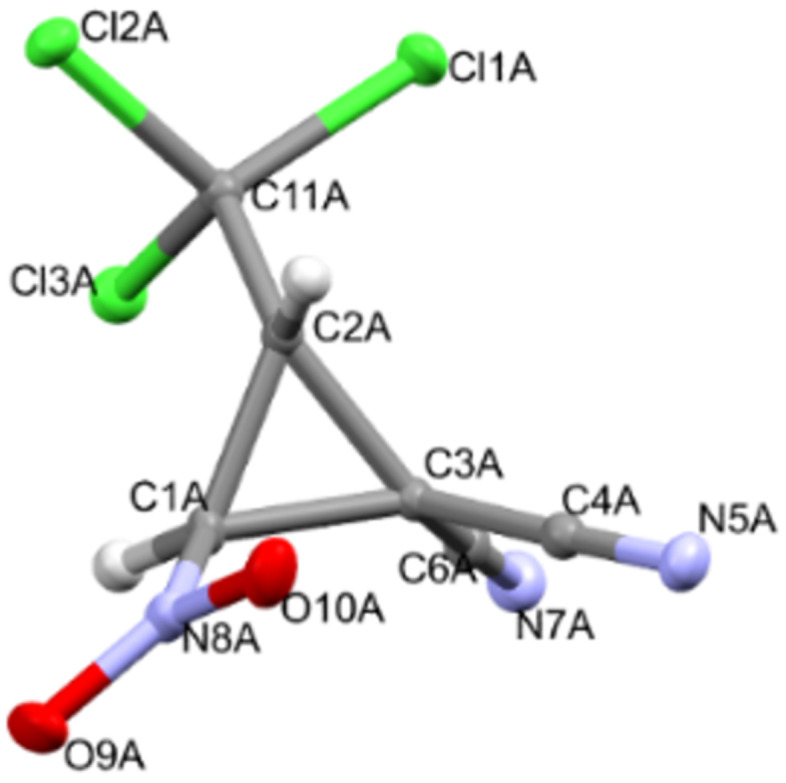
Geometry of **2** in the crystal.

**Figure 3:**
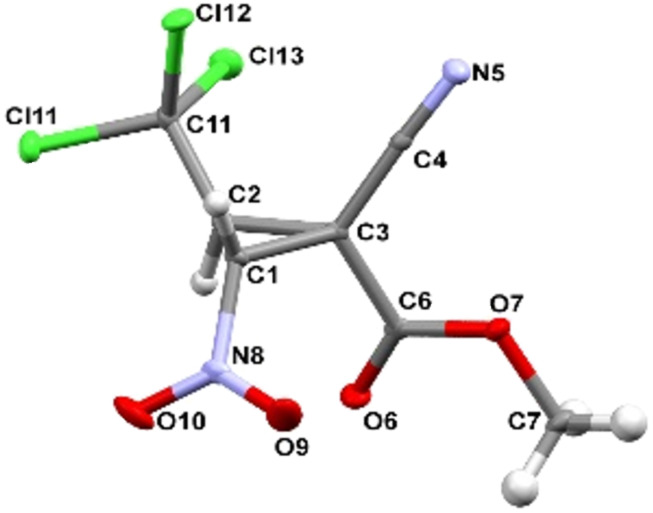
Geometry of **3** in the crystal.

**Figure 4:**
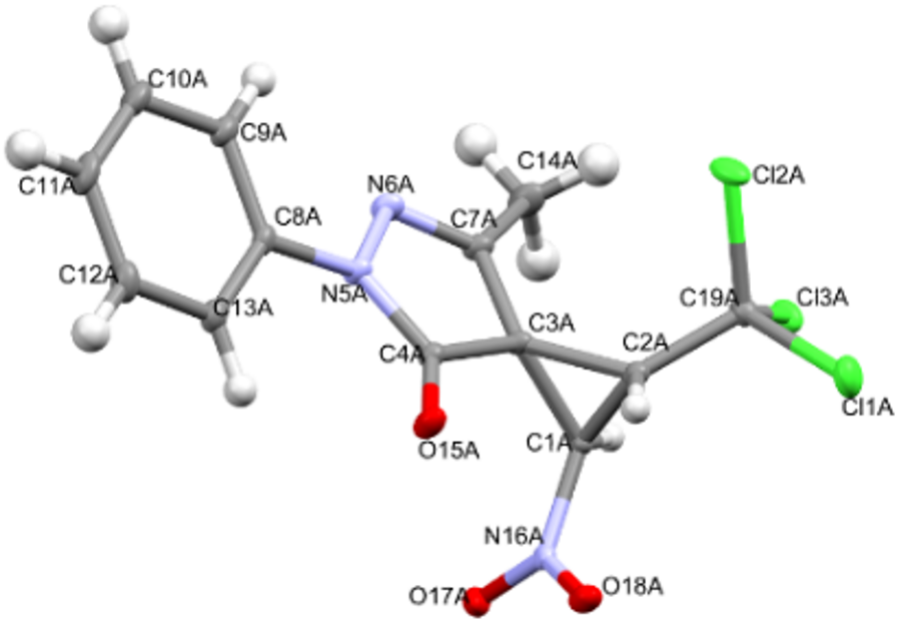
Geometry of **9a** in the crystal.

**Figure 5:**
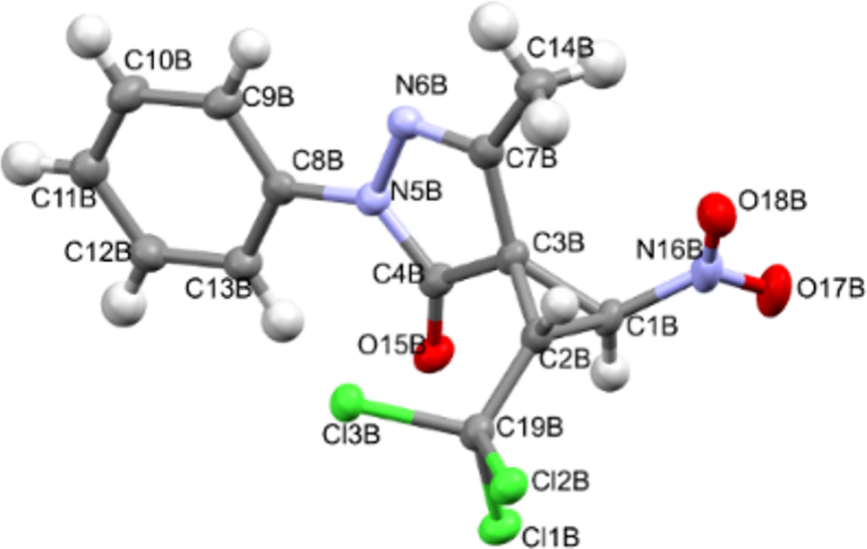
Geometry of **9b** in the crystal.

X-ray diffraction analysis data for compounds **2**, **3**, **9a**, and **9b** convincingly confirm the accepted structures, the position of cyclopropane protons, and the relative configurations of asymmetric atoms (Figures 2–5). It should be noted that the lengths of the C^1^–C^2^ (1.470(2)–1.491(4) Å) and C^2^–NO_2_ (1.472(4)–1.486(2) Å) bonds according to X-ray diffraction analysis in the molecules of nitrocyclopropanes **2**, **3**, **9a**, and **9b** turn out to be close to those in the molecules of nitrospirocyclopropanecarboxylates (C^1^–C^2^ (1.464(1)–1.474(2) Å), C^2^–NO_2_ (1.482(1)–1.485(1) Å) [39] and fused nitrocyclopropane (C^1^–C^2^ 1.4903(19) Å and C^2^–NO_2_ 1.4811(17) Å) [46].

In the crystal of **2**, the polar non-centrosymmetric space group contains two independent molecules with the same configuration of atoms C^1^ – *S*, C^2^ – *R*. That is, compound **2** is obtained as a racemic diastereomeric pair 1*SR*,2*RS*. Centrosymmetric crystals **9a** and **9b** are diastereomers and crystallize as a true racemate. The configuration of the chiral atoms C^1^ and C^2^ in the molecules is the same as in molecules **2**, and the difference is the configuration of atom C^3^. In crystal **9a**, the enatiomeric pair *S,R,S*/*R,S,R* is realized, and in crystal **9b** – *S,R,R*/*R,S,S*.

The main geometric parameters (bond lengths and valence angles) and the conformation of independent molecules in crystals of **2**, **3**, **9a** and **9b** coincide within the experimental errors, so Figures 2–5 show the geometry of one of the independent molecules. Note that crystals **2**, **3**, **9a** and **9b** have a relatively high density (*d*_calc_/g cm^−3^ 1.739, 1.748, 1.595 and 1.567) for crystals that do not contain heavy atoms.

In the absence of hydrogen bonds in the crystals of **2**, **3**, **9a** and **9b**, multiple specific interactions of the types such as lone (electron) pair – the π-system of the nitro group, and bonds of the Cl···O, Cl···N, C–H···X (X = Cl, O, N) are realized, which are shown in Figures S47–S57 in Supporting Information File 1. The energy of such interactions can be comparable with the energy of “classical” hydrogen bonds [47].

## Conclusion

In summary, we proposed a diastereoselective method for the synthesis of vicinal trichloromethylnitrocyclopropanes by forming a cyclopropane ring from a trichloromethylnitroethene substrate and an active methylene component. Variation of the methylene component structure – linear (malononitrile, methyl cyanoacetate, ethyl cyanoacetate, benzoylacetonitrile), carbocyclic (1,3-indanedione), and heterocyclic (Meldrum’s acid, dimethylbarbituric acid, 3-methyl-1-phenyl-5-pyrazolone) CH-acids – allows the synthesis of both monocyclic and spirocyclic 1*SR*,2*RS vic*-trichloromethylnitrocyclopropanes under mild conditions. The reaction is carried out either in tetrahydrofuran in the presence of triethylamine or in methanol in the presence of fused potassium acetate. The structures of the isolated individual products were characterized by ^1^H and ^13^C NMR, IR spectroscopy, mass spectrometry, and confirmed by single-crystal X-ray diffraction analysis.

## Experimental

Physicochemical studies were performed using the equipment of the Center for Collective Use "Physicochemical methods for the study of nitro compounds, coordination, biologically active substances and nanostructured materials" of the Interdisciplinary Resource Center for collective use "Modern physicochemical methods for the formation and study of materials for the needs of industry, science and education" Herzen State Pedagogical University of Russia.

Some spectral studies were performed at the Center for Magnetic Resonance, the Center for Chemical Analysis and Materials Research, and the Research Center for X-ray Diffraction Studies of Saint-Petersburg State University, Saint-Petersburg, Russia.

The X-ray diffraction study was performed at the Department of X-ray Diffraction Research of the Multiple-Access Center on the basis of the Laboratory of Diffraction Research Methods of the A. E. Arbuzov Institute of Organic and Physical Chemistry, the Kazan Scientific Center of the Russian Academy of Sciences.

The ^1^H-^13^C{^1^H}, ^1^H-^1^H dqfCOSY, ^1^H-^1^H NOESY, ^1^H-^13^C HMQC, ^1^H-^13^C HMBC NMR spectra were recorded on a Jeol ECX400A spectrometer operating at 399.78 MHz (^1^H), 100.53 MHz (^13^C) in CDCl_3_ using residual signals of the nondeuterated solvent (δH 7.26, δC 77.16) as the references. The vibrational spectra were measured on a Shimadzu IR-Prestige-21 Fourier-transform IR spectrometer in KBr pellets over 400–4000 cm^−1^ range (resolution was 2 cm^−1^). Mass spectra were obtained using a MaXis mass spectrometer (Bruker Daltonik GmbH) equipped with an electrospray ionization source (4.5 eV) and a quadrupole time-of-flight analyzer (ESI–QTOF) in the positive ions detection mode, with methanol (0.1% FA [formic acid]) as solvent.

Isolation of individual diastereomers was carried out by column chromatography on silica gel MN Kieselgel 60 Macherey-Nagel 140–270, eluent was a mixture of solvents hexane–EtOAc, 3:1. The reaction progress and purity of the obtained compounds were controlled by TLC on Silufol UV-254 plates with 3:1 hexane–EtOAc mobile phase. Visualization was performed under UV light (λ 254 nm).

Reagents were obtained from commercial suppliers and used without further purification unless otherwise noted.

**X-ray crystallography.** X-ray diffraction analysis of the structure **2**, **3**, **9a**, and **9b** was performed on a Rigaku 'SuperNova, Single source at offset/far, HyPix3000' automatic four-circle diffractometer with a Hybrid Pixel Array two-dimensional detector and a micro-focus sealed X-ray tube (λ [Cu Kα] = 1.54184 Å) at cooling conditions (100 K). Data collection and processing of diffraction data were performed using an CrysAlisPro 1.171.41.103a (Rigaku OD, 2021) software package. All of the structures were solved by direct methods using the SHELXT program [48] and refined by the full-matrix least squares method over F^2^ using the SHELXL program [49]. All of the calculations were performed in the WinGX software package [49], the calculation of the geometry of the molecules and the intermolecular interactions in the crystals was carried out using the PLATON program [50] and the drawings of the molecules were performed using the MERCURY [51] programs. The non-hydrogen atoms were refined in anisotropic approximation. The hydrogen atoms were placed in geometrically calculated positions and included in the refinement in the “riding” model.

Crystal of **2**, C_6_H_2_Cl_3_N_3_O_2_, M = 254.46, monoclinic, space group *P2_1_*, at 100.4(5) K: *a* = 11.6233(2), *b* = 6.39530(10), *c* = 13.1586(2) Å, β = 96.5730(10), *V* = 971.71(3) Å^3^, *Z* = 4 (two independent molecules), *D*_calc_ = 1.739 g·cm^−3^, μ(Mo Kα) 8.393 mm^−1^, F(000) = 504, 9545 reflections measured (6.762° ≤ 2Θ ≤ 139.896°), 3676 unique (R_int_° = 0.0391, R_sigma_° = 0.0425) which were used in all calculations. Flack parameter 0.299(14), crystal is a racemic twin, and final refinement of this structure was completed as racemic twin. The final R_1_ was 0.0260 (I > 2σ(I)) and wR_2_ was 0.0655 (all data).

Crystal of **3**, C_7_H_5_Cl_3_N_2_O_4_, M = 287.48, monoclinic, space group *P2_1_/n*, at 100.00(10) K: *a* = 8.3468(3), *b* = 6.1819(2), *c* = 21.2056(6) Å, α = 90, β = 93.234(3), γ = 90^o^, *V* = 1092.45(6) Å^3^, *Z* = 4, *D*_calc_ = 1.748 g·cm^−3^, μ(MoKα) 7.658 mm^−1^, F(000) = 576.0, 3763 reflections measured (8.352° ≤ 2Θ ≤ 139.956°), 2056 unique (R_int_ = 0.0223, R_sigma_ = 0.0260) which were used in all calculations. The final R_1_ was 0.0321 (I > 2σ(I)) and wR_2_ was 0.0844 (all data).

Crystal of **9а**, C_13_H_10_Cl_3_N_3_O_3_, M = 362.60, monoclinic, space group *P2_1_/n*, at 100(2) K: *a* = 11.4834(6), *b* = 11.0564(5), *c* = 23.7834(10) Å, α = 90, β = 91.057(4), γ = 90^o^, *V* = 3019.1(2) Å^3^, *Z* = 8 (two independent molecules), *D*_calc_ = 1.595 g·cm^−3^, μ(Mo Kα) 5.651 mm^−1^, F(000) = 1485.2, 21310 reflections measured (7.44° ≤ 2Θ ≤ 139.94°), 5708 unique (R_int_° = 0.0984, R_sigma_° = 0.0556) which were used in all calculations. The final R_1_ was 0.0696 (I>=2u(I)) and wR_2_ was 0.2072 (all data).

Crystal of **9b**, C_13_H_10_Cl_3_N_3_O_3_, M = 362.59, monoclinic, space group *P2_1_/n*, at 100(2) K: *a* = 16.6097(2), *b* = 9.76570(10), *c* = 19.4325(2) Å, α = 90, β = 102.7570(10), γ = 90^o^, *V* = 3074.25(6) Å^3^, *Z* = 8 (two independent molecules), *D*_calc_ = 1.567 g·cm^−3^, μ(Mo Kα) 5.550 mm^−1^, F(000) = 1472.0, 25819 reflections measured (6.346° ≤ 2Θ ≤ 140°), 5821 unique (R_int_° = 0.0567, R_sigma_° = 0.0359) which were used in all calculations. The final R_1_ was 0.0378 (I > 2σ(I)) and wR_2_ was 0.0979 (all data).

## Supporting Information

The crystallographic data of the structure are deposited in the Cambridge Crystal Structure Data Bank (CCDC **2**: 2237758; CCDC **3**: 2481941; CCDC **9a**: 2450586; CCDC **9b**: 2450587). Statistics on the collection of X-ray diffraction data and refinement of the structure are shown in Table S1 in Supporting Information File 1.

File 1 General synthetic procedures, characterization data and copies of IR spectra, ^1^H-^13^C{^1^H}, ^1^H-^1^H dqfCOSY, ^1^H-^1^H NOESY, ^1^H-^13^C HMQC, ^1^H-^13^C HMBC NMR spectra of all synthesized compounds, and crystallographic data for compounds **2**, **3**, **9a**, and **9b**.

## Data Availability

All data that supports the findings of this study is available in the published article and/or the supporting information of this article.
