# Supplementary material for: Highly electrophilic, gem- and spiro-activated trichloromethylnitrocyclopropanes: synthesis and structure
Source: Beilstein J Org Chem. 2026 Jan 14;22:123–30. doi: 10.3762/bjoc.22.5 (PMC12816983; doi:10.3762/bjoc.22.5)
Supplement: File 1 — General synthetic procedures, characterization data and copies of IR spectra, 1H-13C{1H}, 1H-1H dqfCOSY, 1H-1H NOESY, 1H-13C HMQC, 1H-13C HMBC NMR spectra of all synthesized compounds, and crystallographic data for compounds 2, 3, 9a, and 9b. [file Beilstein_J_Org_Chem-22-123-s001.pdf]

BEILSTEIN JOURNAL OF ORGANIC CHEMISTRY

Supporting Information

for

Highly electrophilic, *gem-* and *spiro*-activated trichloromethylnitrocyclopropanes: synthesis and structure

Ilia A. Pilipenko, Mikhail V. Grigoriev, Olga Yu. Ozerova, Igor A. Litvinov, Darya V. Spiridonova, Aleksander V. Vasilyev and Sergey V. Makarenko

Beilstein J. Org. Chem. **2026,** *22,* 123–130. doi:10.3762/bjoc.22.5

General synthetic procedures, characterization data and copies of IR spectra, ^{1}H-$^{13}C\{^{1}H\}$, ^{1}H-^{1}H dqfCOSY, ^{1}H-^{1}H NOESY, ^{1}H-^{13}C HMQC, ^{1}H-^{13}C HMBC NMR spectra of all synthesized compounds, and crystallographic data for compounds 2, 3, 9a, and 9b

License and Terms: This is a supporting information file under the terms of the Creative Commons Attribution License (https://creativecommons.org/licenses/by/4.0). Please note that the reuse, redistribution and reproduction in particular requires that the author(s) and source are credited and that individual graphics may be subject to special legal provisions.
The license is subject to the *Beilstein Journal of Organic Chemistry* terms and conditions: (https://www.beilstein-journals.org/bjoc/terms)

Table of contents

Experimental part………………………..…………………………………………… S4

Figure S1. ^{1}H NMR spectrum of 2-nitro-3-(trichloromethyl)cyclopropane-1,1-dicarbonitrile (**2**) in $CDCl_3$…………………………………………………………… S8

Figure S2. ^{13}C{^{1}H} NMR spectrum of 2-nitro-3-(trichloromethyl)cyclopropane-1,1-dicarbonitrile **2** in $CDCl_3$…………………………………………………………… S8

Figure S3. ^{1}H-^{1}H dqfCOSY NMR spectrum of 2-nitro-3-(trichloromethyl)cyclopropane-1,1-dicarbonitrile (**2**) in $CDCl_3$…………………….… S9

Figure S4. ^{1}H-^{13}C HMQC NMR spectrum of 2-nitro-3-(trichloromethyl)cyclopropane-1,1-dicarbonitrile (**2**) in $CDCl_3$…………………….… S9

Figure S5. ^{1}H-^{13}C HMBC NMR spectrum of 2-nitro-3-(trichloromethyl)cyclopropane-1,1-dicarbonitrile (**2**) in $CDCl_3$…………………….… S10

Figure S6. IR spectrum of 2-nitro-3-(trichloromethyl)cyclopropane-1,1-dicarbonitrile (**2**) in KBr…………………………………………………………..… S10

Figure S7. ^{1}H NMR spectrum of methyl 1-cyano-2-nitro-3-(trichloromethyl)cyclopropanecarboxylate (**3**) in $CDCl_3$…………………………… S11

Figure S8. ^{13}C{^{1}H} NMR spectrum of methyl 1-cyano-2-nitro-3-(trichloromethyl)cyclopropanecarboxylate (**3**) in $CDCl_3$…………………………… S11

Figure S9. ^{1}H-^{13}C HMQC NMR spectrum of methyl 1-cyano-2-nitro-3-(trichloromethyl)cyclopropanecarboxylate (**3**) in $CDCl_3$…………………………… S12

Figure S10. ^{1}H-^{13}C HMBC NMR spectrum of methyl 1-cyano-2-nitro-3-(trichloromethyl)cyclopropanecarboxylate (**3**) in $CDCl_3$…………………………… S12

Figure S11. IR spectrum of methyl 1-cyano-2-nitro-3-(trichloromethyl)cyclopropanecarboxylate (**3**) in $CHCl_3$………………………….. S13

Figure S12. ^{1}H NMR spectrum of ethyl 1-cyano-2-nitro-3-(trichloromethyl)cyclopropanecarboxylate (**4**) in $CDCl_3$…………………………… S13

Figure S13. ^{13}C{^{1}H} NMR spectrum of ethyl 1-cyano-2-nitro-3-(trichloromethyl)cyclopropanecarboxylate (**4**) in $CDCl_3$…………………………… S14

Figure S14. ^{1}H-^{13}C HMQC NMR spectrum of ethyl 1-cyano-2-nitro-3-(trichloromethyl)cyclopropanecarboxylate (**4**) in $CDCl_3$…………………………… S14

Figure S15. ^{1}H-^{13}C HMBC NMR spectrum of ethyl 1-cyano-2-nitro-3-(trichloromethyl)cyclopropanecarboxylate (**4**) in $CDCl_3$…………………………… S15

Figure S16. ^{1}H-^{1}H NOESY NMR spectrum of ethyl 1-cyano-2-nitro-3-(trichloromethyl)cyclopropanecarboxylate (**4**) in $CDCl_3$…………………………….. S15

Figure S17. IR spectrum of ethyl 1-cyano-2-nitro-3-(trichloromethyl)cyclopropanecarboxylate (**4**) in $CHCl_3$………………………….. S16

Figure S18. ^{1}H NMR spectrum of 1-benzoyl-2-nitro-3-(trichloromethyl)cyclopropane-1-carbonitrile (**5**) in $CDCl_3$…..…………………… S16

Figure S19. ^{13}C{^{1}H} NMR spectrum of 1-benzoyl-2-nitro-3-(trichloromethyl)cyclopropane-1-carbonitrile (**5**) in $CDCl_3$………………………… S17

Figure S20. ^{1}H NMR spectrum of 6,6-dimethyl-1-nitro-2-(trichloromethyl)-5,7-dioxaspiro[2.5]octane-4,8-dione (**6**) in $CDCl_3$………………………………………. S17

Figure S21. ^{13}C{^{1}H} NMR spectrum of 6,6-dimethyl-1-nitro-2-(trichloromethyl)-5,7-dioxaspiro[2.5]octane-4,8-dione (**6**) in $CDCl_3$…...…………………………… S18

Figure S22. ^{1}H-^{13}C HMQC NMR spectrum of 6,6-dimethyl-1-nitro-2-(trichloromethyl)-5,7-dioxaspiro[2.5]octane-4,8-dione (**6**) in $CDCl_3$……..……… S18

Figure S23. ^{1}H-^{13}C HMBC NMR spectrum of 6,6-dimethyl-1-nitro-2-(trichloromethyl)-5,7-dioxaspiro[2.5]octane-4,8-dione (**6**) in $CDCl_3$……..……… S19

Figure S24. IR spectrum of 6,6-dimethyl-1-nitro-2-(trichloromethyl)-5,7-dioxaspiro[2.5]octane-4,8-dione (**6**) in KBr ……………..…………………… S19

Figure S25. ^{1}H NMR spectrum of 5,7-dimethyl-1-nitro-2-(trichloromethyl)-5,7-diazaspiro[2.5]octane-4,6,8-trione (**7**) in $CDCl_3$…………………………………… S20

Figure S26. $^{13}C\{^1H\}$ NMR spectrum of 5,7-dimethyl-1-nitro-2-(trichloromethyl)-5,7-diazaspiro[2.5]octane-4,6,8-trione (**7**) in $CDCl_3$…………………………….……. S20
Figure S27. ^1H-^{13}C HMQC NMR spectrum of 5,7-dimethyl-1-nitro-2-(trichloromethyl)-5,7-diazaspiro[2.5]octane-4,6,8-trione (**7**) in $CDCl_3$…..…..….… S21
Figure S28. ^1H-^{13}C HMBC NMR spectrum of 5,7-dimethyl-1-nitro-2-(trichloromethyl)-5,7-diazaspiro[2.5]octane-4,6,8-trione (**7**) in $CDCl_3$…………… S21
Figure S29. IR spectrum of 5,7-dimethyl-1-nitro-2-(trichloromethyl)-5,7-diazaspiro[2.5]octane-4,6,8-trione (**7**) in KBr ………………………………………. S22
Figure S30. ^1H NMR spectrum of 2-nitro-3-(trichloromethyl)spiro[cyclopropane-1,2'-indene]-1',3'-dione (**8**) in $CDCl_3$……………………….……………………… S22
Figure S31. $^{13}C\{^1H\}$ NMR spectrum of 2-nitro-3-(trichloromethyl)spiro[cyclopropane-1,2'-indene]-1',3'-dione (**8**) in $CDCl_3$……… S23
Figure S32. ^1H-^{13}C HMQC NMR spectrum of 2-nitro-3-(trichloromethyl)spiro[cyclopropane-1,2'-indene]-1',3'-dione (**8**) in $CDCl_3$…..…… S23
Figure S33. ^1H-^{13}C HMBC NMR spectrum of 2-nitro-3-(trichloromethyl)spiro[cyclopropane-1,2'-indene]-1',3'-dione (**8**) in $CDCl_3$………. S24
Figure S34. IR spectrum of 2-nitro-3-(trichloromethyl)spiro[cyclopropane-1,2'-indene]-1',3'-dione (**8**) in KBr……………………………………………………..……… S24
Figure S35. ^1H NMR spectrum of 7-methyl-1-nitro-5-phenyl-2-(trichloromethyl)-5,6-diazaspiro[2.4]hept-6-en-4-one (**9a**) in $CDCl_3$………………………………… S25
Figure S36. $^{13}C\{^1H\}$ NMR spectrum of 7-methyl-1-nitro-5-phenyl-2-(trichloromethyl)-5,6-diazaspiro[2.4]hept-6-en-4-one (**9a**) in $CDCl_3$……………… S25
Figure S37. ^1H-^{13}C HMQC NMR spectrum of 7-methyl-1-nitro-5-phenyl-2-(trichloromethyl)-5,6-diazaspiro[2.4]hept-6-en-4-one (**9a**) in $CDCl_3$…………….. S26
Figure S38. ^1H-^{13}C HMBC NMR spectrum of 7-methyl-1-nitro-5-phenyl-2-(trichloromethyl)-5,6-diazaspiro[2.4]hept-6-en-4-one (**9a**) in $CDCl_3$……………… S26
Figure S39. ^1H-^1H NOESY NMR spectrum of 7-methyl-1-nitro-5-phenyl-2-(trichloromethyl)-5,6-diazaspiro[2.4]hept-6-en-4-one (**9a**) in $CDCl_3$……………… S27
Figure S40. IR spectrum of 7-methyl-1-nitro-5-phenyl-2-(trichloromethyl)-5,6-diazaspiro[2.4]hept-6-en-4-one (**9a**) in KBr ………………………………………… S27
Figure S41. ^1H NMR spectrum of 7-methyl-1-nitro-5-phenyl-2-(trichloromethyl)-5,6-diazaspiro[2.4]hept-6-en-4-one (**9b**) in $CDCl_3$………………………………….. S28
Figure S42. $^{13}C\{^1H\}$ NMR spectrum of 7-methyl-1-nitro-5-phenyl-2-(trichloromethyl)-5,6-diazaspiro[2.4]hept-6-en-4-one (**9b**) in $CDCl_3$……………… S28
Figure S43. ^1H-^{13}C HMQC NMR spectrum of 7-methyl-1-nitro-5-phenyl-2-(trichloromethyl)-5,6-diazaspiro[2.4]hept-6-en-4-one (**9b**) in $CDCl_3$……………… S29
Figure S44. ^1H-^{13}C HMBC NMR spectrum of 7-methyl-1-nitro-5-phenyl-2-(trichloromethyl)-5,6-diazaspiro[2.4]hept-6-en-4-one (**9b**) in $CDCl_3$……………… S29
Figure S45. ^1H-^1H NOESY NMR spectrum of 7-methyl-1-nitro-5-phenyl-2-(trichloromethyl)-5,6-diazaspiro[2.4]hept-6-en-4-one (**9b**) in $CDCl_3$……………… S30
Figure S46. IR spectrum of 7-methyl-1-nitro-5-phenyl-2-(trichloromethyl)-5,6-diazaspiro[2.4]hept-6-en-4-one (**9b**) in KBr ………………………………………… S30
Table S1. Principal crystallographic parameters of compound **2**, **3**, **9a**, **9b** based on X-ray diffraction data……………………………………………………………… S31
Table S2. Bond lengths for **2**…………………………………………………………… S32
Table S3. Bond angles for **2**…………………………………………………………… S32
Table S4. Torsion angles for **2**………………………………………………………… S33
Table S5. Bond lengths for **3**…………………………………………………………… S33
Table S6. Bond angles for **3**…………………………………………………………… S34
Table S7. Torsion angles for **3**………………………………………………………… S35
Table S8. Bond lengths for **9a**………………………………………………………… S35
Table S9. Bond angles for **9a**………………………………………………………… S35
Table S10. Torsion angles for **9a**……………………………………………………… S36
Table S11. Bond lengths for **9b**………………………………………………………… S38

Table S12. Bond angles for **9b**……………..…….…………………….…….……….… S38
Table S13. Torsion angles for **9b**…………………..……………….………..……… S39
Figure S47. Geometry of 2 independent molecules (A and B) **2** in crystal S41
Figure S48. System of short contacts in crystal **2**………………...…………… S41
Figure S49. Crystal packaging of **2**……………..….………………………… S42
Figure S50. Geometry of 2 independent molecules (A and B) **3** in crystal S42
Figure S51. Crystal packaging of **3**……………..….………………………… S43
Figure S52. Geometry of 2 independent molecules **9a** in crystal …………… S43
Figure S53. System of short contacts in crystal **9a** ….………………………... S43
Figure S54. Fragment of **9a** crystal packaging ……………………………….… S44
Figure S55. Geometry of 2 independent molecules **9b** in crystal ………..…… S44
Figure S56. System of short contacts in crystal **9b** ….……………...……… S45
Figure S57. Fragment of **9b** crystal packaging……………………………….… S45
References………………………………………………….……………….… S46

Experimental part

1-Bromo-1-nitro-3,3,3-trichloropropene (**1**) was obtained by the literature method [5].

*2-Nitro-3-(trichloromethyl)cyclopropane-1,1-dicarbonitrile (**2**)*

Method **A**.

A solution of 1-bromo-1-nitro-3,3,3-trichloropropene (**1**, 190 mg, 0.71 mmol) in anhydrous MeOH (5 ml) was added to a solution of malononitrile (46 mg, 0.71 mmol) and AcOK (70 mg, 0.71 mmol) in anhydrous MeOH (15 ml). The mixture was stirred at room temperature for 15 min, poured into the crushed ice, extracted with chloroform (3 × 20 ml), the organic phase was dried over $MgSO_4$. After removal of chloroform the tarred residue was crystallized with EtOH treatment. The yield of compound **2** is 32 mg (18%), a light yellow solid, m.p. 98–99°C (EtOH).

Method **C**.

A solution of 1-bromo-1-nitro-3,3,3-trichloropropene (**1**, 190 mg, 0.71 mmol) in anhydrous THF (5 ml) was added to a solution of malononitrile (46 mg, 0.71 mmol) and AcOK (70 mg, 0.71 mmol) in anhydrous THF (15 ml). The mixture was stirred at room temperature for 2 h and poured into the crushed ice, extracted with chloroform (3 × 20 ml), the organic phase was dried over $MgSO_4$. After removal of chloroform the tarred residue was crystallized with EtOH treatment. The yield of compound **2** is 36 mg (20%), a light yellow solid, m.p. 97–99°C (EtOH).

Method **E**.

A solution of 1-bromo-1-nitro-3,3,3-trichloropropene (**1**, 190 mg, 0.71 mmol) in anhydrous THF (5 ml) was added to a solution of malononitrile (78 mg, 1.18 mmol) and triethylamine (79 mg, 0.78 mmol) in anhydrous THF (15 ml). The mixture was stirred at room temperature for 1 h, the precipitate (triethylammonium bromide) was filtered out, the filtrate was poured into the crushed ice, extracted with chloroform (3 × 20 ml), the organic phase was dried over $MgSO_4$. After removal of chloroform the tarred residue was crystallized with EtOH treatment. The yield of compound **2** is 113 mg (64%), a light yellow solid, m.p. 98–100°C (EtOH).

A test of mixing samples obtained by methods **A**, **C** and **E** did not result in melting point depression.

^{1}H NMR (400 MHz, $CDCl_3$) δ, ppm: 4.25 d (1H, ^3J 6.2 Hz, C^3H), 5.42 d (1H, ^3J 6.2 Hz, C^2H).

^{13}C NMR (100 MHz, $CDCl_3$) δ, ppm: 14.9 (C^1), 48.7 (C^3), 65.4 (C^2), 88.4 (CCl_3), 106.6 (CN), 107.4 (CN).

IR (KBr) ν, cm^{-1}: 582, 593, 616, 651, 732, 761, 796, 807 (νCCl_3), 828, 969, 1006, 1016, 1061, 1106, 1195, 1220, 1370 ($\nu_s NO_2$), 1406 (δCH), 1569, 1577 ($\nu_{as} NO_2$), 2262 (νCN), 2850, 2925, 3011, 3091 (νCH).

HRMS (ESI): m/z calc. $C_6H_3Cl_3N_3O_2$ [M+H] 253.9285, found 253.9291.

X-ray: CCDC – 2237758.

*Methyl 1-cyano-2-nitro-3-(trichloromethyl)cyclopropanecarboxylate (**3**)*

Method **E**.

A solution of 1-bromo-1-nitro-3,3,3-trichloropropene (**1**, 184 mg, 0.68 mmol) in anhydrous THF (5 ml) was added to a solution of methyl cyanoacetate (113 mg, 1.14 mmol) and triethylamine (77 mg, 0.76 mmol) in anhydrous THF (15 ml). The mixture was stirred at room temperature for 2 h, the precipitate (triethylammonium bromide) was filtered out, the filtrate was poured into the crushed ice, extracted with chloroform (3 × 20 ml), the organic phase was dried over $MgSO_4$. After removal of chloroform the tarred residue was crystallized with EtOH treatment. The yield of compound **3** is 122 mg (62%), a light yellow solid, m.p. 106–108°C (EtOH).

^{1}H NMR (400 MHz, $CDCl_3$) δ, ppm: 3.96 s (3H, CH_3), 4.26 d (1H, ^3J 6.4 Hz, C^3H), 5.31 d (1H, ^3J 6.4 Hz, C^2H).

^{13}C NMR (100 MHz, $CDCl_3$) δ, ppm: 28.9 (C^1), 48.4 (C^3), 55.8 (CH_3), 67.1 (C^2), 90.2 (CCl_3), 110.0 (CN), 160.6 (C=O).
IR ($CHCl_3$) ν, cm^{-1}: 716, 751, 799, 811 (νCCl_3), 933, 1163, 1214, 1220, 1308, 1357 ($\nu_s NO_2$), 1437 (δCH), 1572 ($\nu_{as} NO_2$), 1759 (C=O) 2255 (νCN), 3029 (νCH).
HRMS (ESI): m/z calc. $C_7H_5Cl_3N_2O_4$ [M-H] 284.9237, found 284.9242.
X-ray: CCDC – 2481941.

*Ethyl 1-cyano-2-nitro-3-(trichloromethyl)cyclopropanecarboxylate (**4**)*

Method **E**.

A solution of 1-bromo-1-nitro-3,3,3-trichloropropene (**1**, 219 mg, 0.81 mmol) in anhydrous THF (5 ml) was added to a solution of ethyl cyanoacetate (123 mg, 1.08 mmol) and triethylamine (91 mg, 0.90 mmol) in anhydrous THF (15 ml). The mixture was stirred at room temperature for 2 h, the precipitate (triethylammonium bromide) was filtered out, the filtrate was poured into the crushed ice, extracted with chloroform (3 × 20 ml), the organic phase was dried over $MgSO_4$. After removal of chloroform the tarred residue was kept in a vacuum desiccator for 24 hours. The yield of compound **4** is 174 mg (72%), an yellow oil (Rf 0.60, hexane/ethyl acetate 3:2).
^1H NMR (400 MHz, $CDCl_3$) δ, ppm: 1.38 t (3H, ^3J 7.2 Hz, CH_3), 4.25 d (1H, ^3J 6.4 Hz, C^3H), 4.37 dq (1H, ^2J 10.7, ^3J 7.2 Hz, CH'), 4.41 dq (1H, ^2J 10.7, ^3J 7.2 Hz, CH''), 5.30 d (1H, *J* 6.4 Hz, C^2H).
^{13}C NMR (100 MHz, $CDCl_3$) δ, ppm: 13.9 (CH_3), 29.1 (C^1), 48.2 (C^3), 65.8 (CH_2), 67.1 (C^2), 90.2 (CCl_3), 110.2 (CN), 159.9 (C=O).
IR ($CHCl_3$) ν, cm^{-1}: 675, 715, 732, 754, 800, 810 (νCCl_3), 853, 989, 1006, 1098, 1159, 1180, 1221, 1261, 1306, 1357, 1371 ($\nu_s NO_2$), 1446, 1465 (δCH), 1571 ($\nu_{as} NO_2$), 1754 (C=O) 2255 (νCN), 2987, 3028 (νCH).
HRMS (ESI): m/z calc. $C_8H_7Cl_3N_2O_4$ [M-H] 298.9393, found 298.9399.

*1-Benzoyl-2-nitro-3-(trichloromethyl)cyclopropane-1-carbonitrile (**5**)*

Method **E**.

A solution of 1-bromo-1-nitro-3,3,3-trichloropropene (**1**, 92 mg, 0.34 mmol) in anhydrous THF (2 ml) was added to a solution of 3-oxo-3-phenylpropannitrile (47 mg, 0.32 mmol) and triethylamine (38 mg, 0.38 mmol) in anhydrous THF (6 ml). The mixture was stirred at room temperature for 1 h. Then it was poured into water (20 ml), extracted with dichloromethane (3 × 20 ml), the organic phase was dried over Na_2SO_4. After removal of chloroform the tarred residue was purified by preparative TLC (EtOAc/petroleum ether, 1/9). The yield of compound **5** is 40 mg (37%), a light yellow oil (R_f 0.57, hexane/ethyl acetate 3:2).
^1H NMR (400 MHz, $CDCl_3$) δ, ppm: 4.42 d (1H, ^3J 6.2 Hz, C^3H), 5.61 d (1H, ^3J 6.2 Hz, C^2H), 7.59 m (2H, H-*o*), 7.73 m (1H, H-*p*), 8.04 m (2H, H-*m*).
^{13}C NMR (100 MHz, $CDCl_3$) δ, ppm: 33.3 (C^1), 46.3 (C^3), 67.4 (C^2), 90.7 (CCl_3), 111.3 (CN), 129.4, 129.5, 131.9, 135.9 (Ph), 180.3 (C=O).
HRMS (ESI): m/z calc. $C_{12}H_8Cl_3N_3O_2$ [M+H] 332.9595, found 332.9598.

*6,6-dimethyl-1-nitro-2-(trichloromethyl)-5,7-dioxaspiro[2.5]octane-4,8-dione (**6**)*

Method **A**.

A solution of fused potassium acetate (106 mg, 1.079 mmol) in anhydrous MeOH (5 ml) was added to a suspension of Meldrum's acid (104 mg, 0.719 mmol) in anhydrous MeOH (5 ml), followed by a solution of 1-bromo-1-nitro-3,3,3-trichloropropene (**1**, 194 mg, 0.719 mmol) in anhydrous methanol (5 ml). The mixture was stirred at room temperature for 24 h. Then it was poured into crushed ice, extracted with chloroform (3 × 20 ml), the organic phase was dried with $MgSO_4$. The solvent was evaporated on a rotary evaporator. After removal of chloroform the tarred residue was crystallized with EtOH treatment. The yield of compound **6** is 44 mg (19%), a colorless solid, m.p. 123–125°C (EtOH).

^1H NMR (400 MHz, $CDCl_3$) δ, ppm: 1.89 s (3H, CH_3), 1.96 s (3H, CH_3), 4.57 d (1H, *J* 7.4 Hz, C^3H), 5.82 d (1H, *J* 7.4 Hz, C^2H).
^{13}C NMR (100 MHz, $CDCl_3$) δ, ppm: 28.0 (CH_3), 28.6 (CH_3), 39.8 (C^1), 56.0 (C^3), 70.6 (C^2), 90.8 (CCl_3), 107.5 (C^6), 158.3 (C=O), 161.6 (C=O).
IR (KBr) ν, cm^{-1}: 689, 702, 780, 794, 808, 849 (νCCl_3), 914, 952, 1003, 1056, 1069, 1083, 1166, 1208, 1249, 1302, 1328, 1364 ($ν_sNO_2$), 1387, 1398, 1423, 1443 (δCH), 1573 ($ν_{as}NO_2$), 1751, 1779 (νC=O), 2851, 2924, 2956, 3029, 3048 (νCH).
HRMS (ESI): m/z calc. $C_9H_8Cl_3NO_6$ [M-H] 329.9339, found 329.9344.

*5,7-Dimethyl-1-nitro-2-(trichloromethyl)-5,7-diazaspiro[2.5]octane-4,6,8-trione (**7**)*

Method **A**.

A solution of fused potassium acetate (109 mg, 1.113 mmol) in anhydrous MeOH (3 ml) was added to a suspension of *N,N'*-dimethylbarbituric acid (116 mg, 0.742 mmol) in anhydrous methanol (4 ml), followed by a solution of 1-bromo-1-nitro-3,3,3-trichloropropene (**1**, 200 mg, 0.742 mmol) in anhydrous methanol (3 ml). The mixture was stirred at room temperature for 3 h. Then it was poured into crushed ice, the brine was added and the precipitate was filtered out. The yield of compound **7** is 118 mg (47%), a white solid, m.p. 147–149 °C (EtOH).
^1H NMR (400 MHz, $CDCl_3$) δ, ppm: 3.36 s (3H, CH_3), 3.39 s (3H, CH_3), 4.46 d (1H, ^3J 7.2 Hz, C^3H), 5.92 d (1H, ^3J 7.2 Hz, C^2H).
^{13}C NMR (100 MHz, $CDCl_3$) δ, ppm: 29.7 (CH_3), 29.8 (CH_3), 42.4 (C^1), 58.2 (C^3), 70.9 (C^2), 91.0 (CCl_3), 150.1 (C=O), 160.2 (C=O), 162.5 (C=O).
IR (KBr) ν, cm^{-1}: 751, 779, 801, 816 (νCCl_3), 840, 965, 1009, 1026, 1037, 1079, 1089, 1140, 1198, 1258, 1282, 1315, 1367, 1375 ($ν_sNO_2$), 1419, 1430, 1448, 1459 (δCH), 1564 ($ν_{as}NO_2$), 1681, 1684, 1751 (νC=O), 2967, 3023, 3045 (νCH).
HRMS (ESI): m/z calc. $C_9H_8Cl_3N_3O_5$ [M-H] 341.9451, found 341.9457.

*2-Nitro-3-(trichloromethyl)spiro[cyclopropane-1,2'-indene]-1',3'-dione (**8**)*

Method **A**.

A solution of fused potassium acetate (164 mg, 1.67 mmol) in anhydrous MeOH (4 ml) was added dropwise to a mixture of 1,3-indanedione (163 mg, 1.113 mmol) and 1-bromo-1-nitro-3,3,3-trichloropropene (**1**, 300 mg, 1.113 mmol) in anhydrous MeOH (8 ml). The mixture was stirred at room temperature for 3 h. Then it was poured into crushed ice, the brine was added and the precipitate was filtered out. The yield of compound **8** is 250 mg (67%), a pale brown solid, m.p. 160–162 °C (EtOH).
^1H NMR (400 MHz, $CDCl_3$) δ, ppm: 4.50 d (1H, ^3J 6.4 Hz, C^3H), 5.74 d (1H, *J* 6.4 Hz, C^2H), 7.94 m (2H, $C^{8,9}H$), 8.07 m (2H, $C^{7,10}H$).
^{13}C NMR (100 MHz, $CDCl_3$) δ, ppm: 46.3 (C^1), 55.1 (C^3), 70.3 (C^2), 91.3 (CCl_3), 124.1, 124.3 ($C^{7,10}$), 136.5, 136.7 ($C^{8,9}$), 141.1, 142.7 ($C^{6,11}$), 188.5 (C=O), 189.5 (C=O).
IR (KBr) ν, cm^{-1}: 587, 640, 689, 724, 750, 769, 784, 793, 809 (νCCl_3), 853, 956, 985, 1031, 1081, 1112, 1150, 1157, 1182, 1210, 1259, 1289, 1317, 1326, 1354, 1367 ($ν_sNO_2$), 1432, 1568 ($ν_{as}NO_2$), 1592 (νAr), 1716, 1744, 1759 (νC=O), 2918, 3020, 3071 (νCH).
HRMS (ESI): m/z calc. $C_{12}H_6Cl_3NO_4$ [M+H] 333.9441, found 333.9435.

7-Methyl-1-nitro-5-phenyl-2-(trichloromethyl)-5,6-diazaspiro[2.4]hept-6-en-4-one
***9a** (rel-1S,2R,3S), **9b** (rel-1S,2R,3R)*

Method **A**.

A solution of fused potassium acetate (109 mg, 1.113 mmol) in anhydrous methanol (3 ml) was added to a suspension of 3-methyl-1-phenyl-5-pyrazolone (129 mg, 0.742 mmol), followed by a solution of 1-bromo-1-nitro-3,3,3-trichloropropene (**1**, 200 mg, 0.742 mmol) in anhydrous methanol (3 ml). The mixture was stirred at room temperature for 3 h. Then it was

poured into crushed ice, extracted with chloroform (3 × 20 ml), the organic phase was dried with $MgSO_4$. After removal of chloroform the tarred residue was chromatographed on a silica gel column with (hexane/EtOAc, 3/1). The yield of diastereomer **9a** was 60 mg (23%), a colorless solid, m.p. 110–112 °C (EtOH), diastereomer **9b** – 50 mg (19%), a colorless solid, m.p. 154-157 °C (EtOH).

9a ^{1}H NMR (400 MHz, $CDCl_3$) δ, ppm: 2.15 s (3H, CH_3), 4.44 d (1H, ^3J 6.7 Hz, C^3H), 5.75 d (1H, J 6.7 Hz, C^2H), 7.25 m (1H, H-*p*), 7.42 m (2H, H-*m*), 7.85 m (2H, H-*o*).

^{13}C NMR (100 MHz, $CDCl_3$) δ, ppm: 14.7 (CH_3), 47.8 (C^1), 52.9 (C^3), 73.4 (C^2), 90.8 (CCl_3), 119.1 ($C^{8,12}$), 126.1 (C^{10}), 129.1 ($C^{9,11}$), 137.5 (C^7), 151.1 (C=O), 163.5 (C=O).

IR (KBr) ν, cm^{-1}: 692, 724, 740, 761, 786, 792, 799 (νCCl_3), 829, 847, 958, 1004, 1031, 1058, 1067, 1083, 1094, 1125, 1159, 1168, 1177, 1237, 1314, 1371 ($\nu_s NO_2$), 1402, 1427, 1434, 1460, 1491 (δCH), 1502 (δPhN), 1565, 1573 ($\nu_{as} NO_2$), 1596 (νAr), 1712 (νC=O), 3033, 3064 (νCH).

HRMS (ESI): m/z calc. $C_{13}H_{10}Cl_3N_3O_3$ [M+H] 361.9866, found 361.9861.

X-ray: CCDC – 2450586.

9b ^{1}H NMR (400 MHz, $CDCl_3$) δ, ppm: 2.38 s (3H, CH_3), 4.36 d (1H, ^3J 6.6 Hz, C^3H), 5.54 d (1H, ^3J 6.6 Hz, C^2H), 7.25 m (1H, H-*p*), 7.42 m (2H, H-*m*), 7.88 m (2H, H-*o*).

^{13}C NMR (100 MHz, $CDCl_3$) δ, ppm: 18.0 (CH_3), 45.3 (C^1), 56.0 (C^3), 69.6 (C^2), 91.5 (CCl_3), 118.9 ($C^{8,12}$), 126.1 (C^{10}), 129.2 ($C^{9,11}$), 137.4 (C^7), 151.1 (C=O), 164.4 (C=O).

IR (KBr) ν, cm^{-1}: 686, 691, 724, 735, 761, 786, 791, 799 (νCCl_3), 958, 1058, 1094, 1125, 1168, 1177, 1314, 1335, 1371 ($\nu_s NO_2$), 1402, 1434, 1460, 1491 (δCH), 1501 (δPhN), 1564, 1573 ($\nu_{as} NO_2$), 1597 (νAr), 1712 (νC=O), 2849, 2920, 2955, 3033, 3065 (νCH).

HRMS (ESI): m/z calc. $C_{13}H_{10}Cl_3N_3O_3$ [M-H] 359.9715, found 359.9709.

X-ray: CCDC – 2450587.

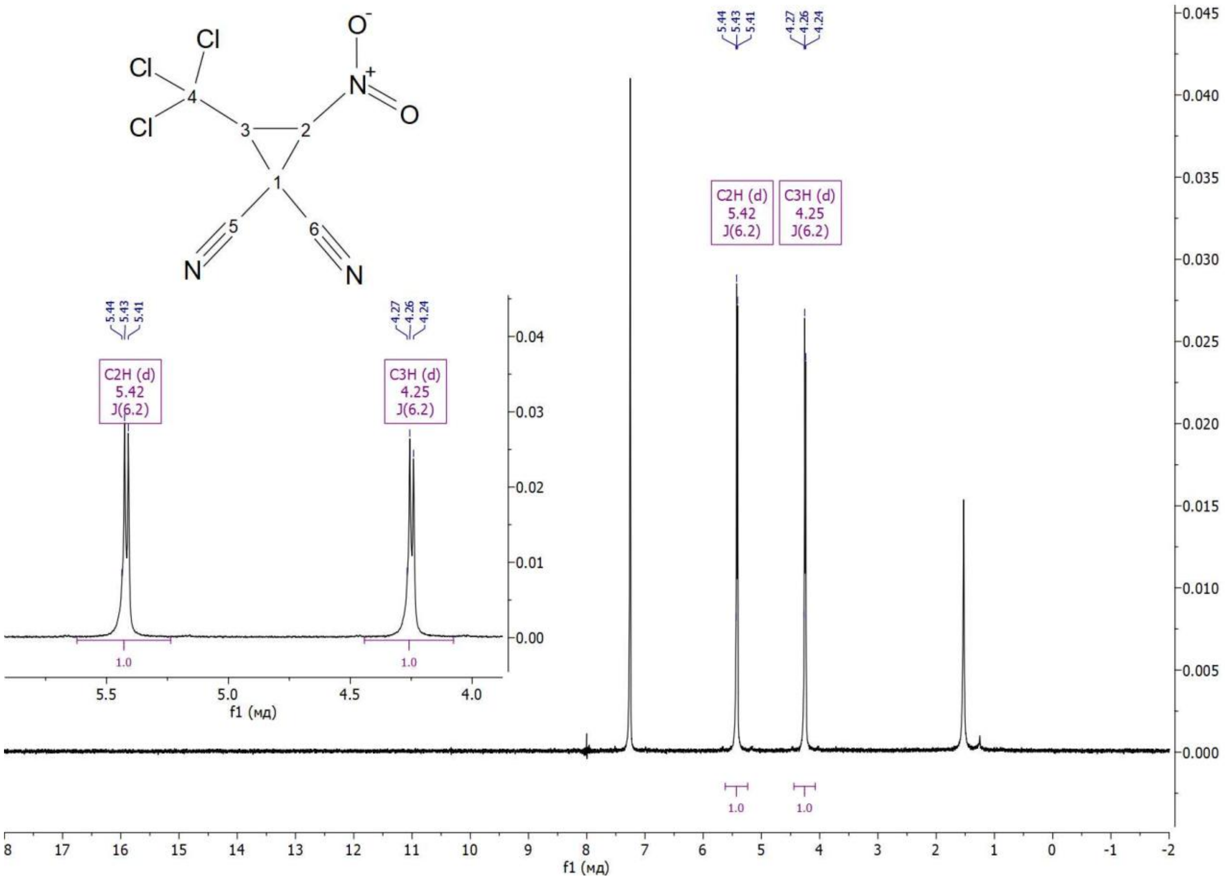

Figure S1. ^{1}H NMR spectrum of 2-nitro-3-(trichloromethyl)cyclopropane-1,1-dicarbonitrile (**2**) in $CDCl_3$.

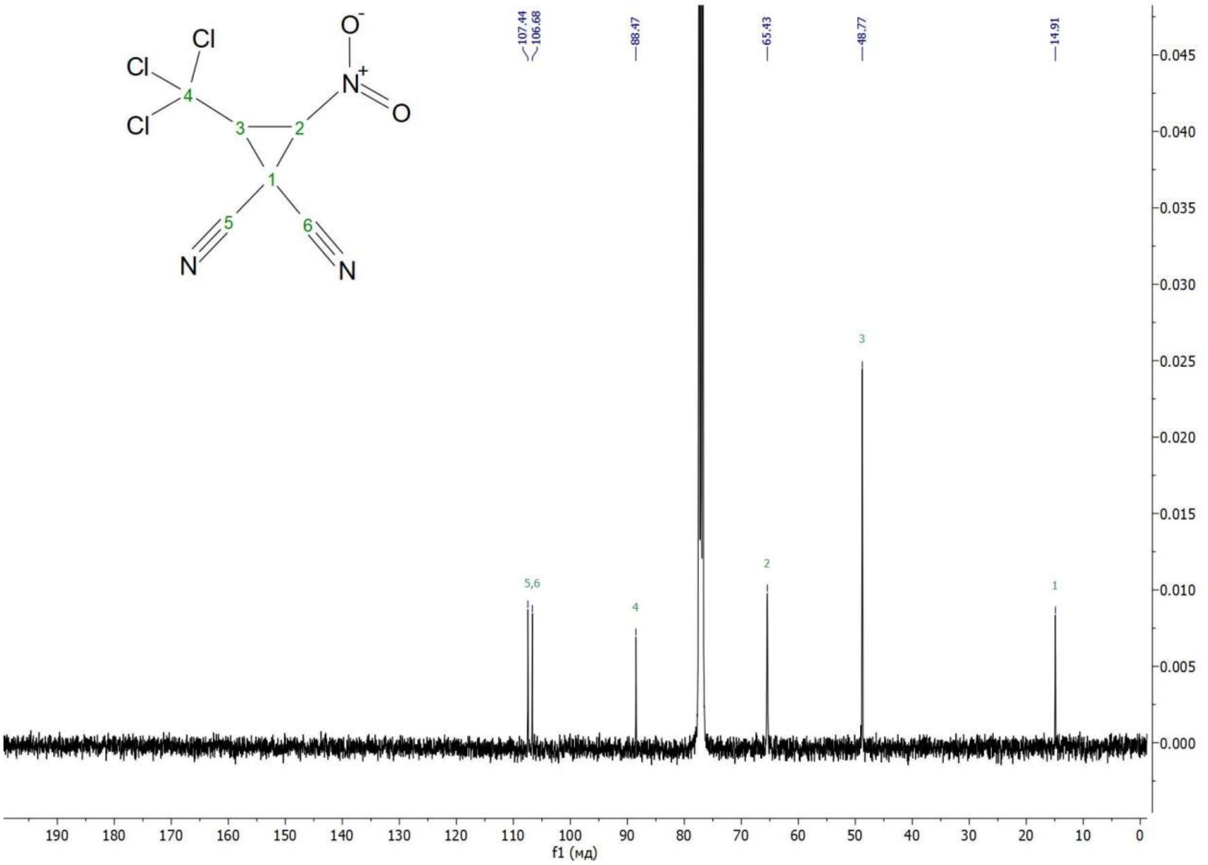

Figure S2. $^{13}C\{^{1}H\}$ NMR spectrum of 2-nitro-3-(trichloromethyl)cyclopropane-1,1-dicarbonitrile (**2**) in $CDCl_3$.

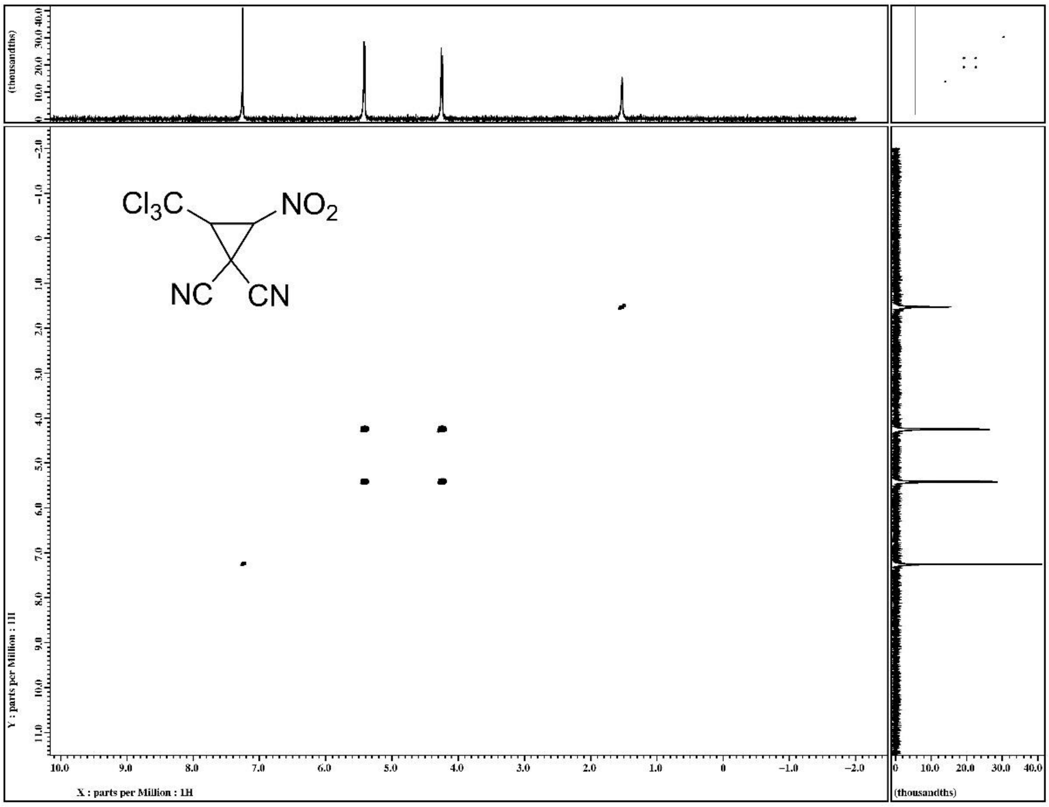

Figure S3. ^{1}H-^{1}H dqfCOSY NMR spectrum of 2-nitro-3-(trichloromethyl)cyclopropane-1,1-dicarbonitrile (**2**) in $CDCl_3$.

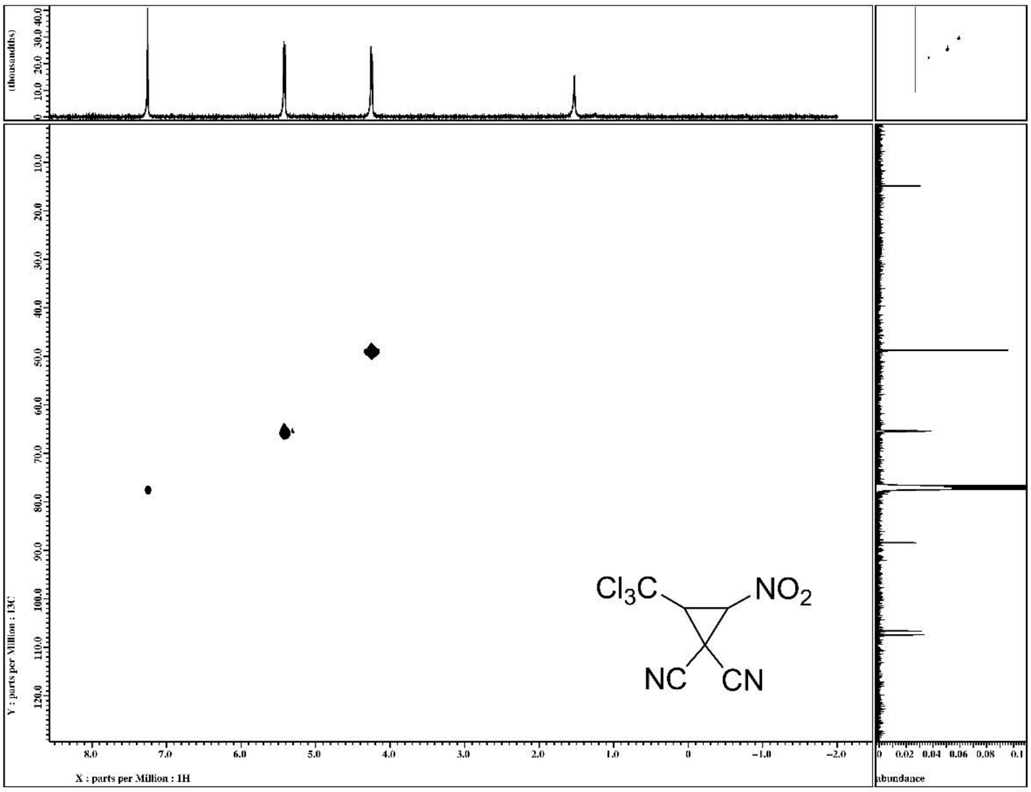

Figure S4. ^{1}H-^{13}C HMQC NMR spectrum of 2-nitro-3-(trichloromethyl)cyclopropane-1,1-dicarbonitrile (**2**) in $CDCl_3$.

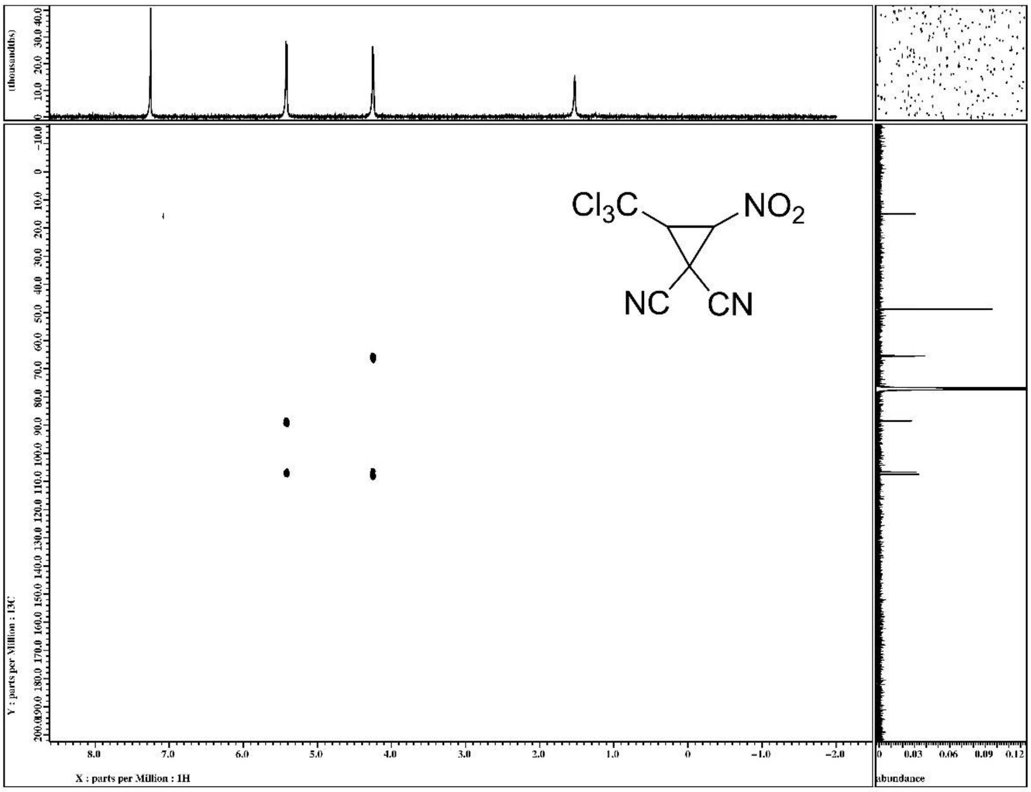

Figure S5. ^{1}H-^{13}C HMBC NMR spectrum of 2-nitro-3-(trichloromethyl)cyclopropane-1,1-dicarbonitrile (**2**) in $CDCl_3$.

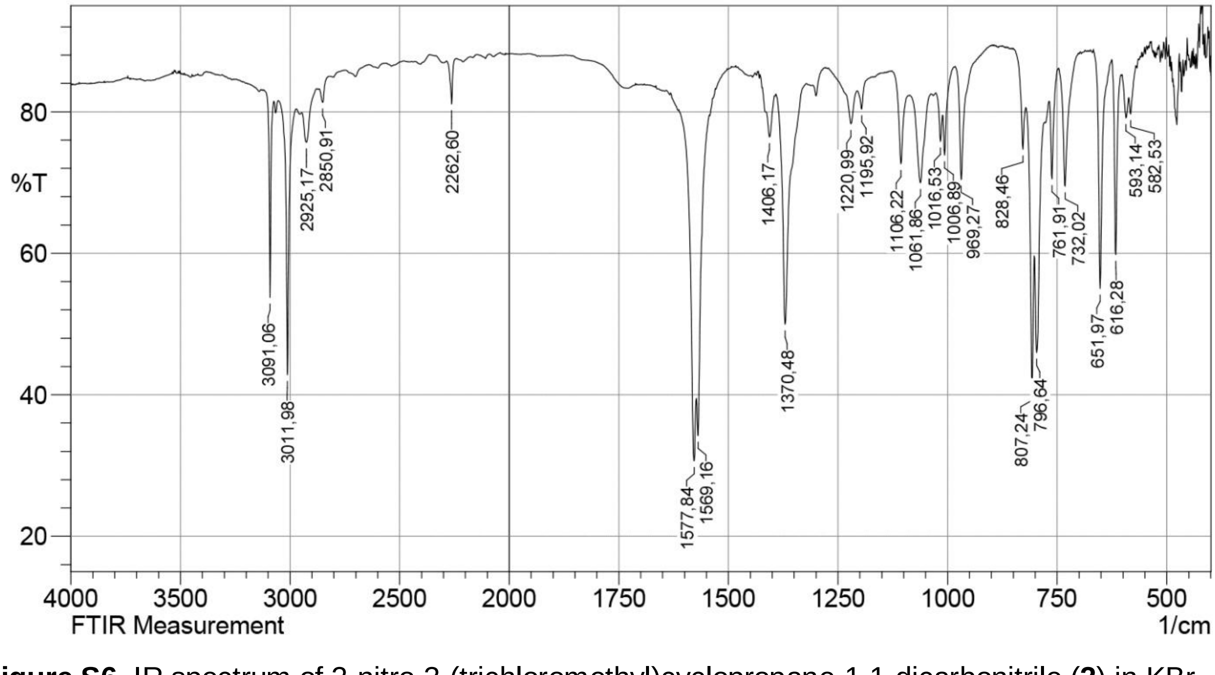

Figure S6. IR spectrum of 2-nitro-3-(trichloromethyl)cyclopropane-1,1-dicarbonitrile (**2**) in KBr.

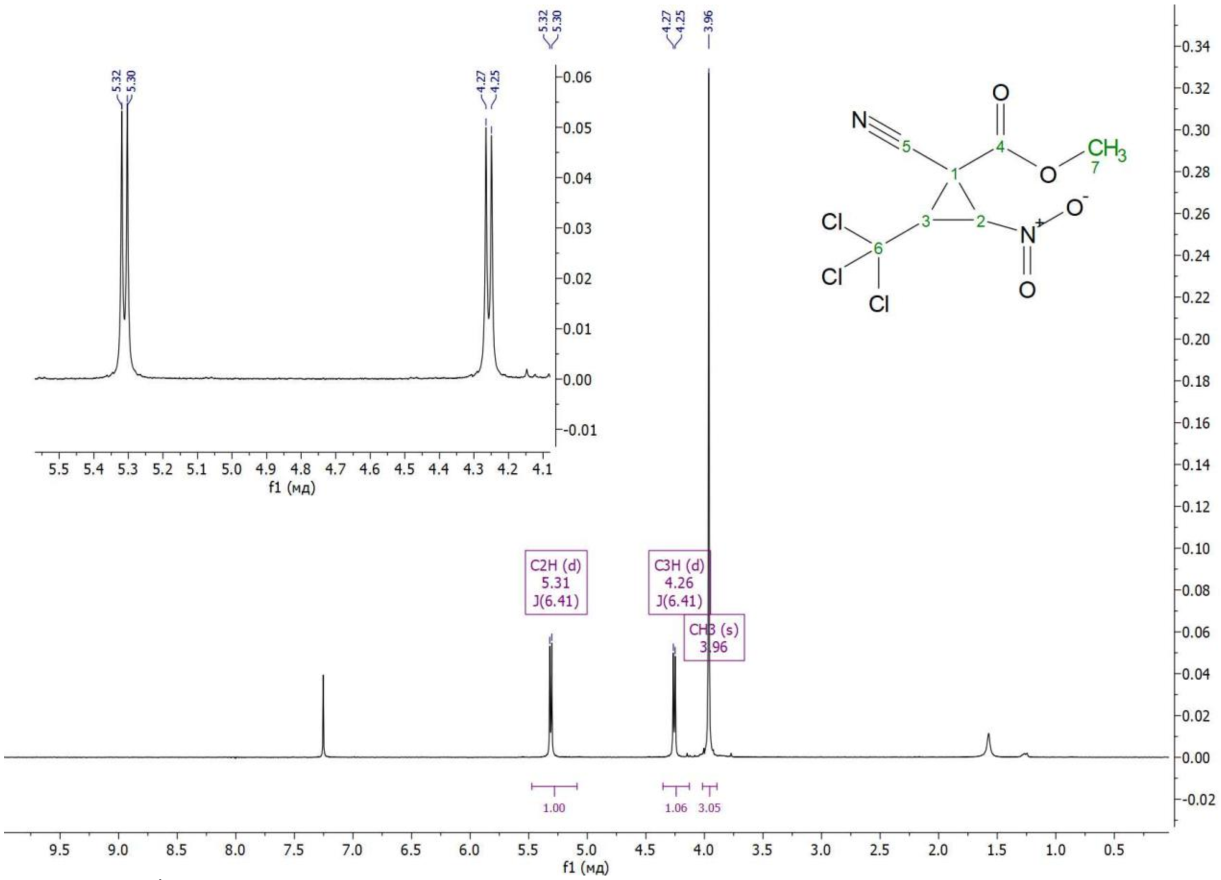

Figure S7. ^{1}H NMR spectrum of methyl 1-cyano-2-nitro-3-(trichloromethyl)cyclopropanecarboxylate (**3**) in $CDCl_3$.

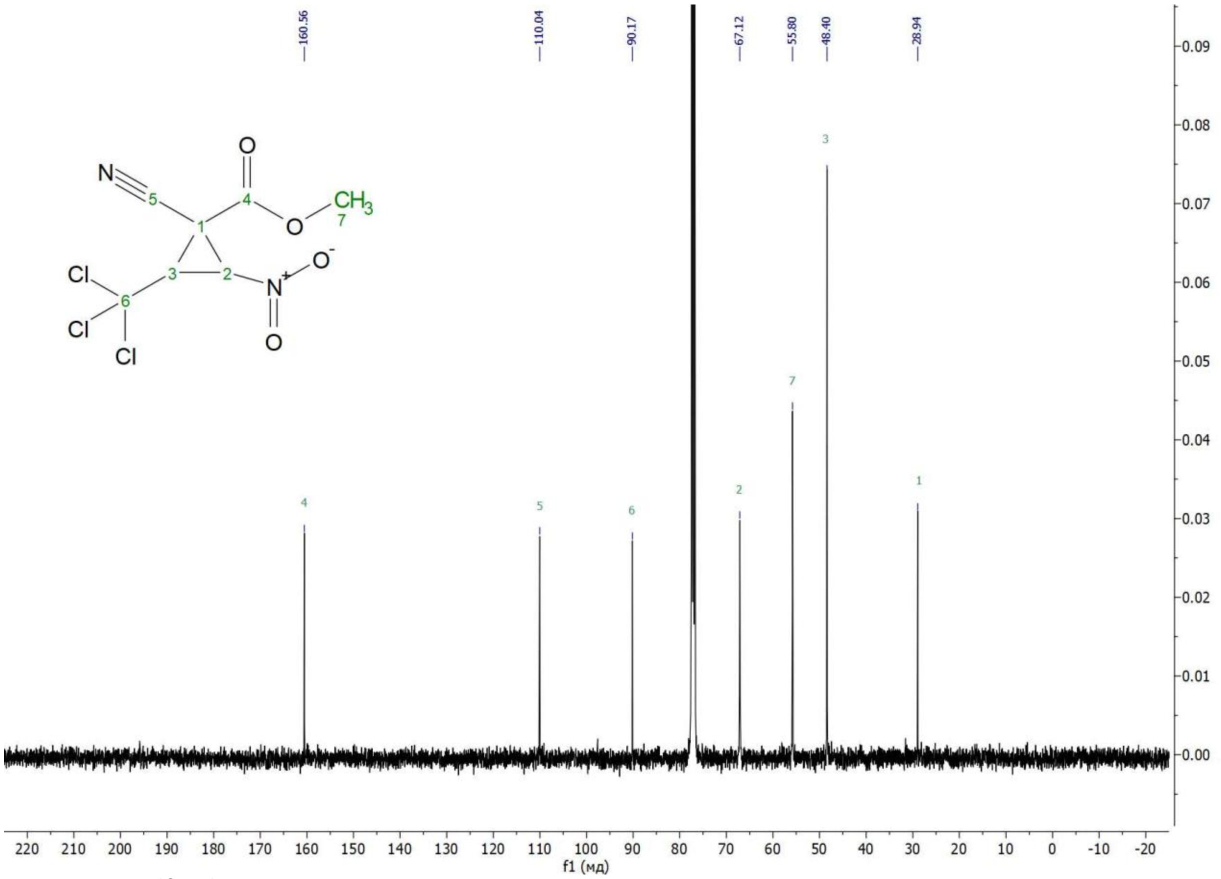

Figure S8. $^{13}C\{^{1}H\}$ NMR spectrum of methyl 1-cyano-2-nitro-3-(trichloromethyl)cyclopropanecarboxylate (**3**) in $CDCl_3$.

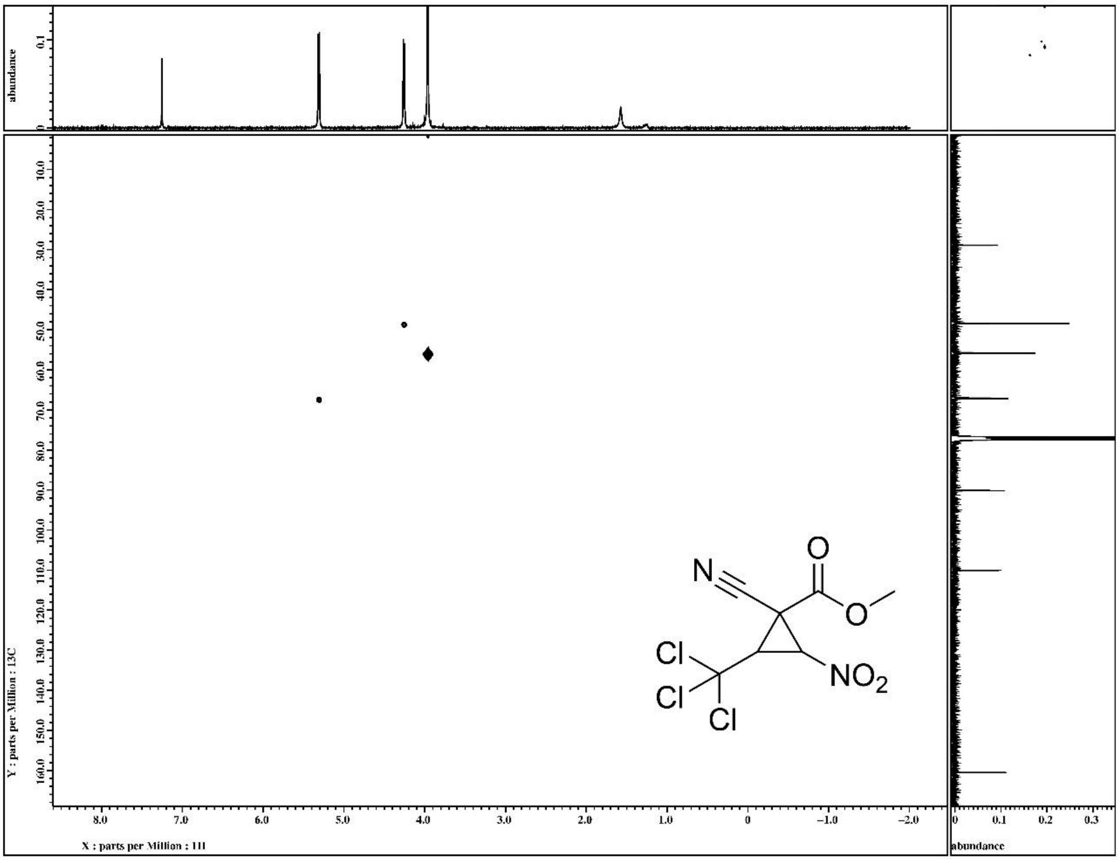

Figure S9. ^{1}H-^{13}C HMQC NMR spectrum of methyl 1-cyano-2-nitro-3-(trichloromethyl)cyclopropanecarboxylate (**3**) in $CDCl_3$.

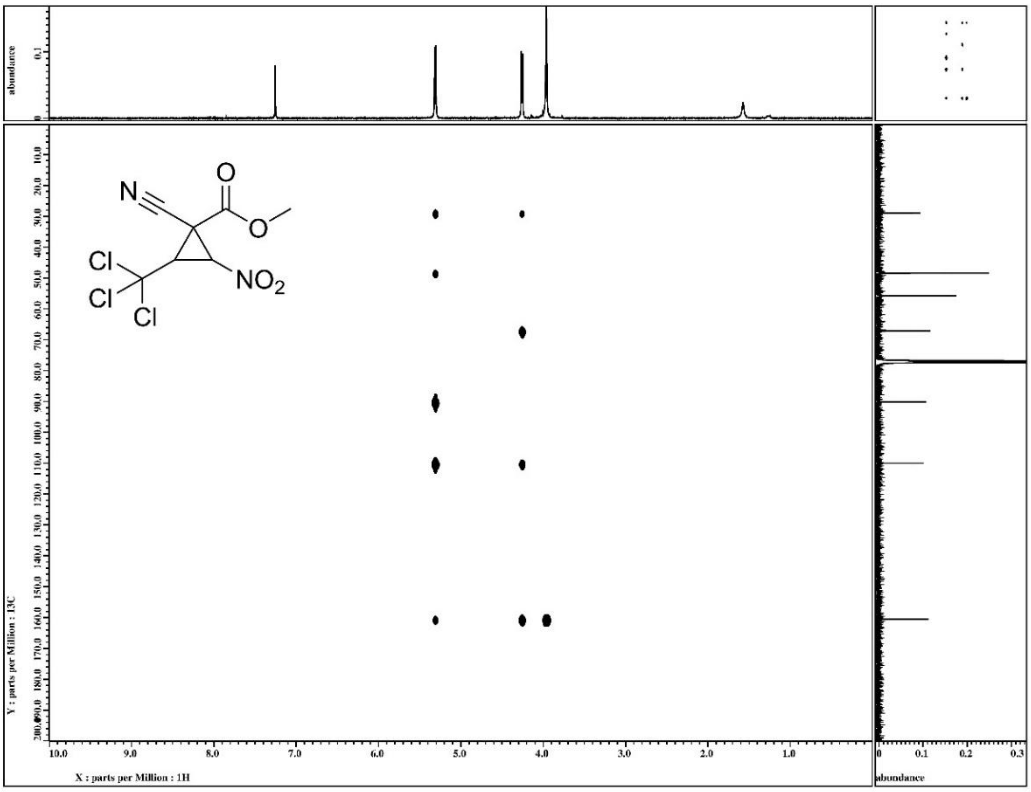

Figure S10. ^{1}H-^{13}C HMBC NMR spectrum of methyl 1-cyano-2-nitro-3-(trichloromethyl)cyclopropanecarboxylate (**3**) in $CDCl_3$.

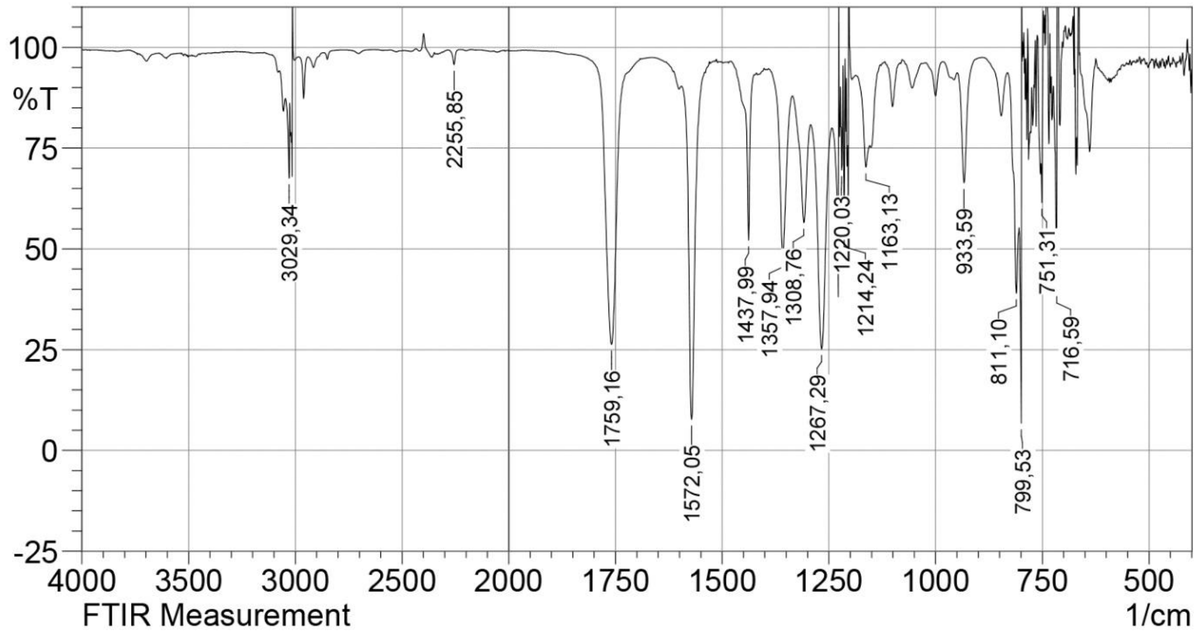

Figure S11. IR spectrum of methyl 1-cyano-2-nitro-3-(trichloromethyl)cyclopropanecarboxylate (**3**) in $CHCl_3$.

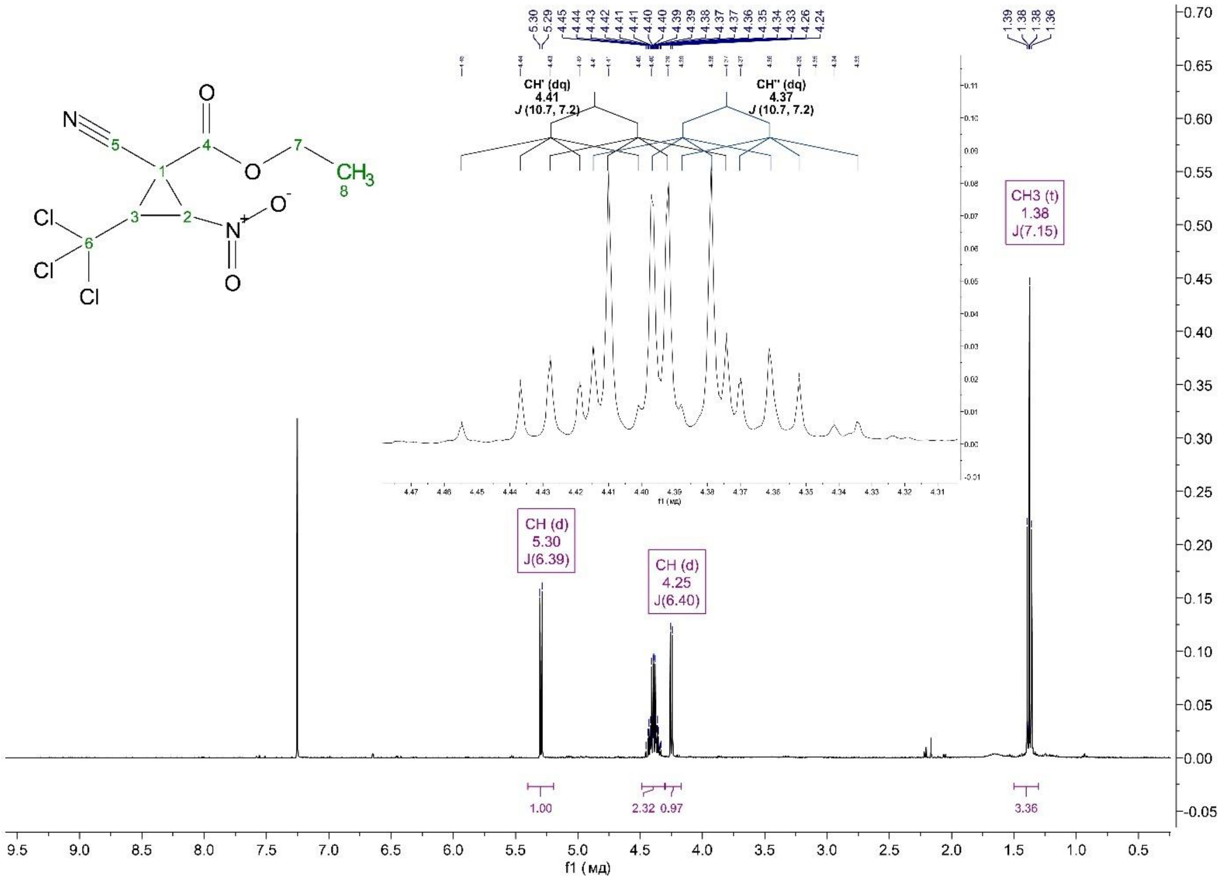

Figure S12. ^{1}H NMR spectrum of ethyl 1-cyano-2-nitro-3-(trichloromethyl)cyclopropanecarboxylate (**4**) in $CDCl_3$.

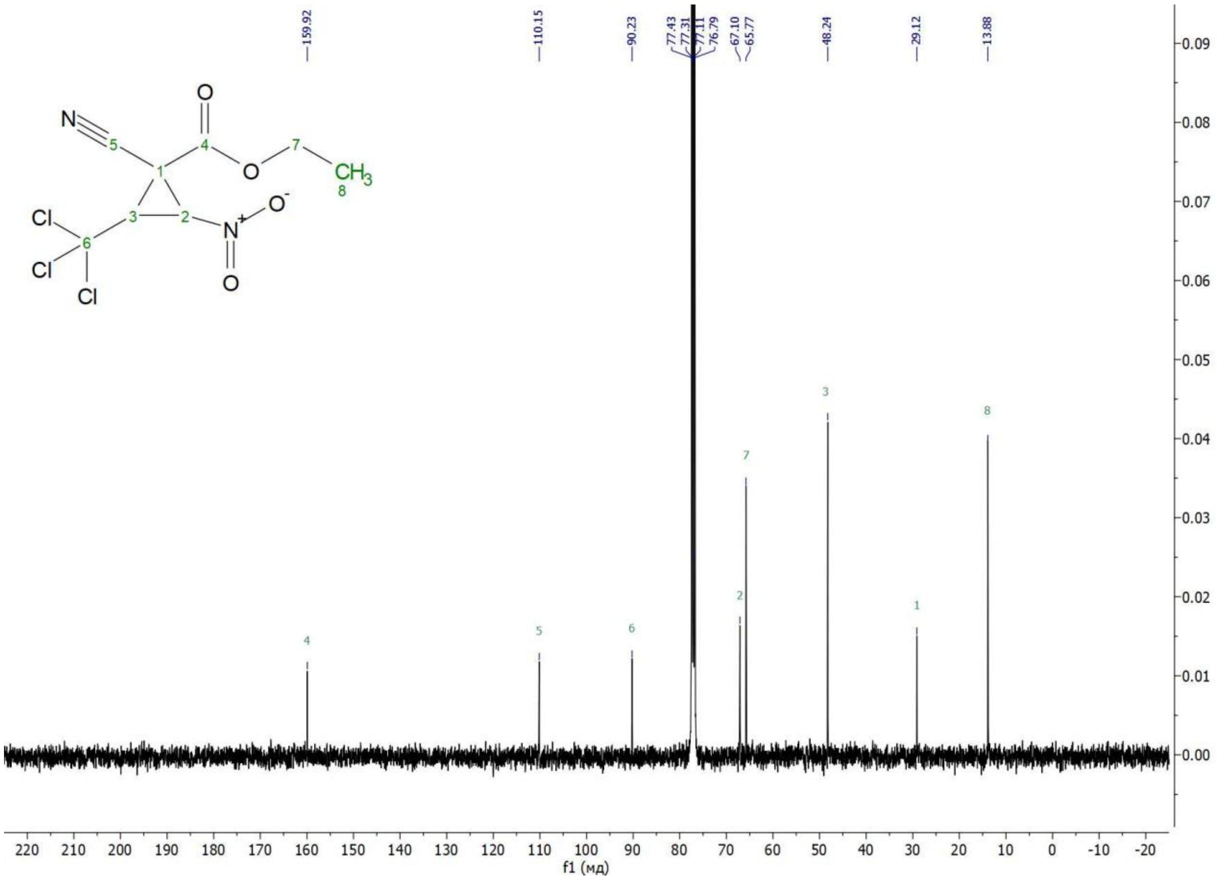

Figure S13. $^{13}C\{^{1}H\}$ NMR spectrum of ethyl 1-cyano-2-nitro-3-(trichloromethyl)cyclopropanecarboxylate (**4**) in $CDCl_3$.

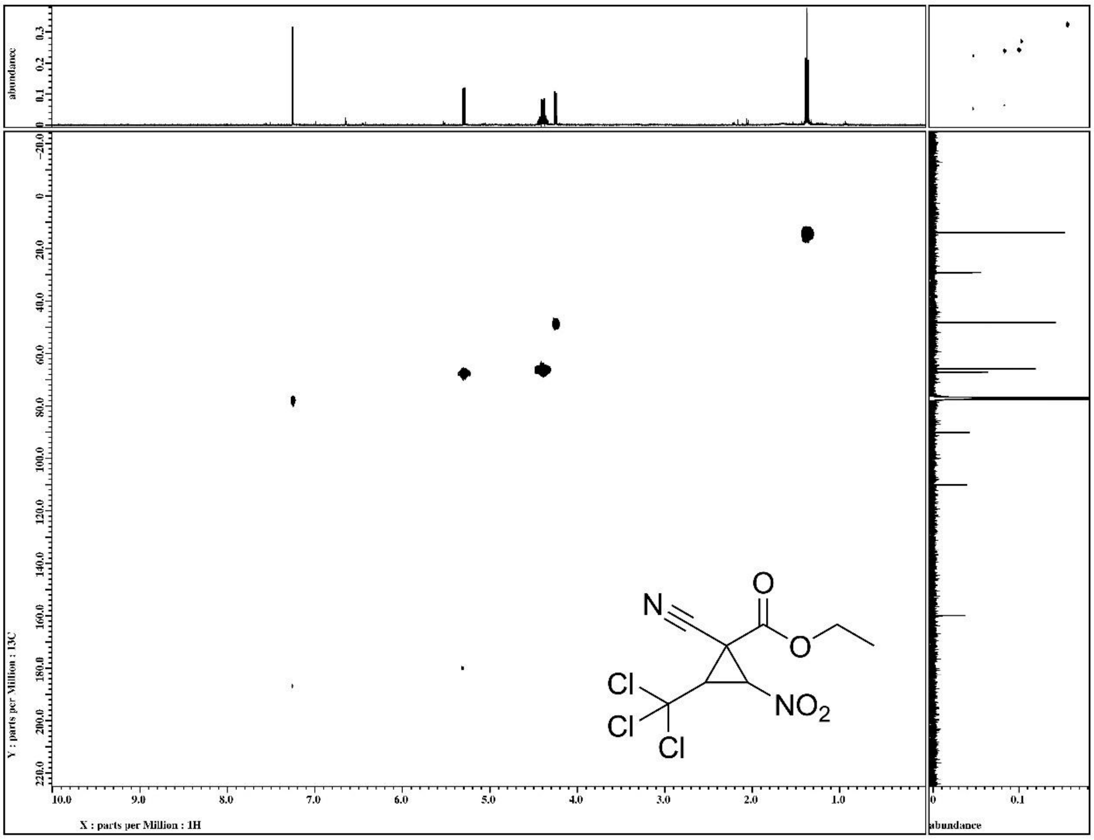

Figure S14. ^{1}H-^{13}C HMQC NMR spectrum of ethyl 1-cyano-2-nitro-3-(trichloromethyl)cyclopropanecarboxylate (**4**) in $CDCl_3$.

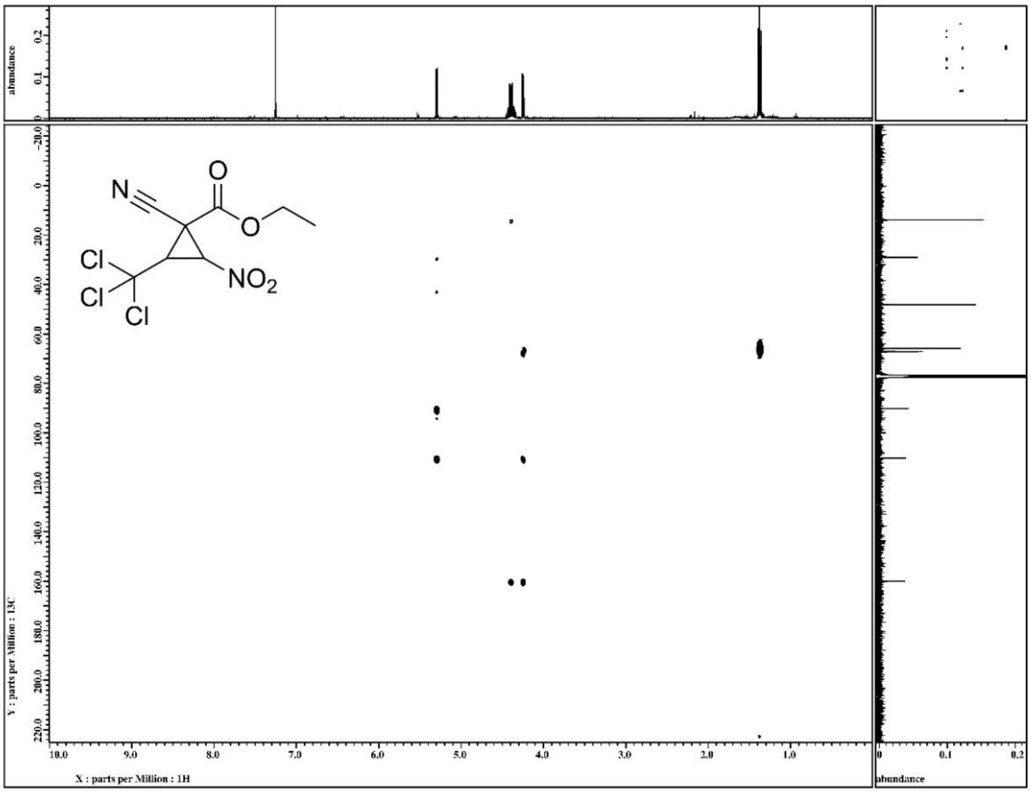

Figure S15. ^{1}H-^{13}C HMBC NMR spectrum of ethyl 1-cyano-2-nitro-3-(trichloromethyl)cyclopropanecarboxylate (**4**) in $CDCl_3$.

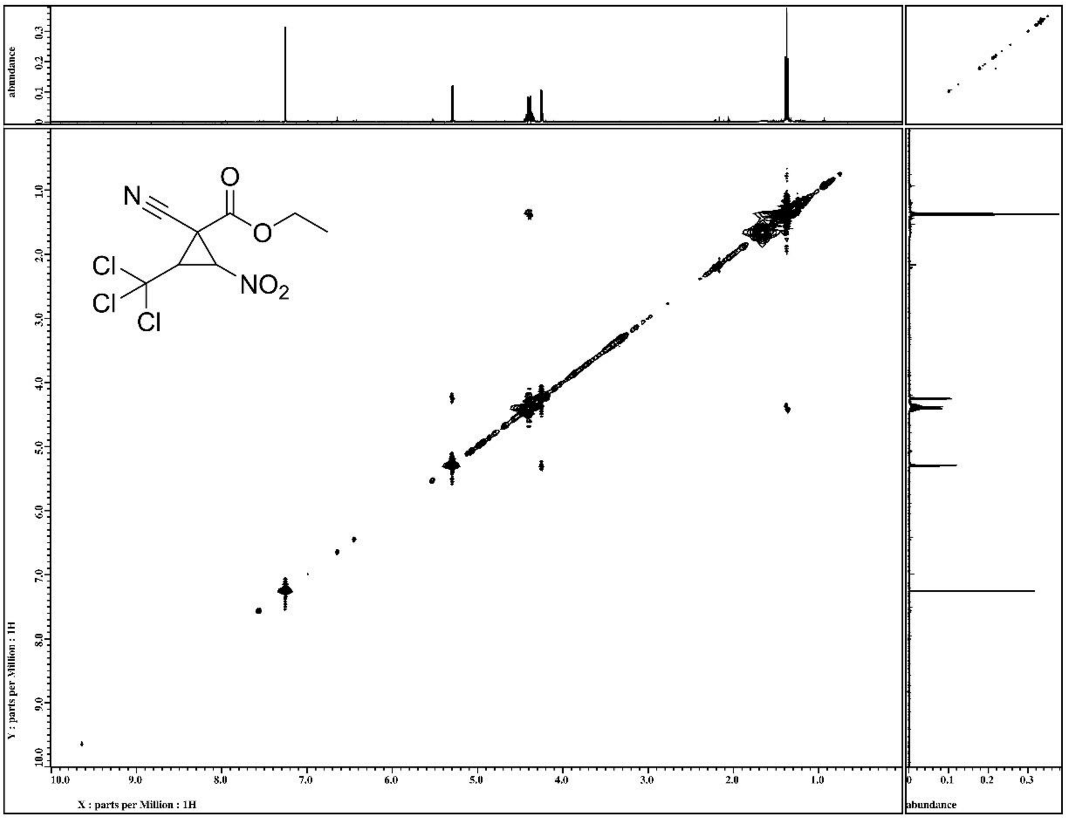

Figure S16. ^{1}H-^{1}H NOESY NMR spectrum of ethyl 1-cyano-2-nitro-3-(trichloromethyl)cyclopropanecarboxylate (**4**) in $CDCl_3$.

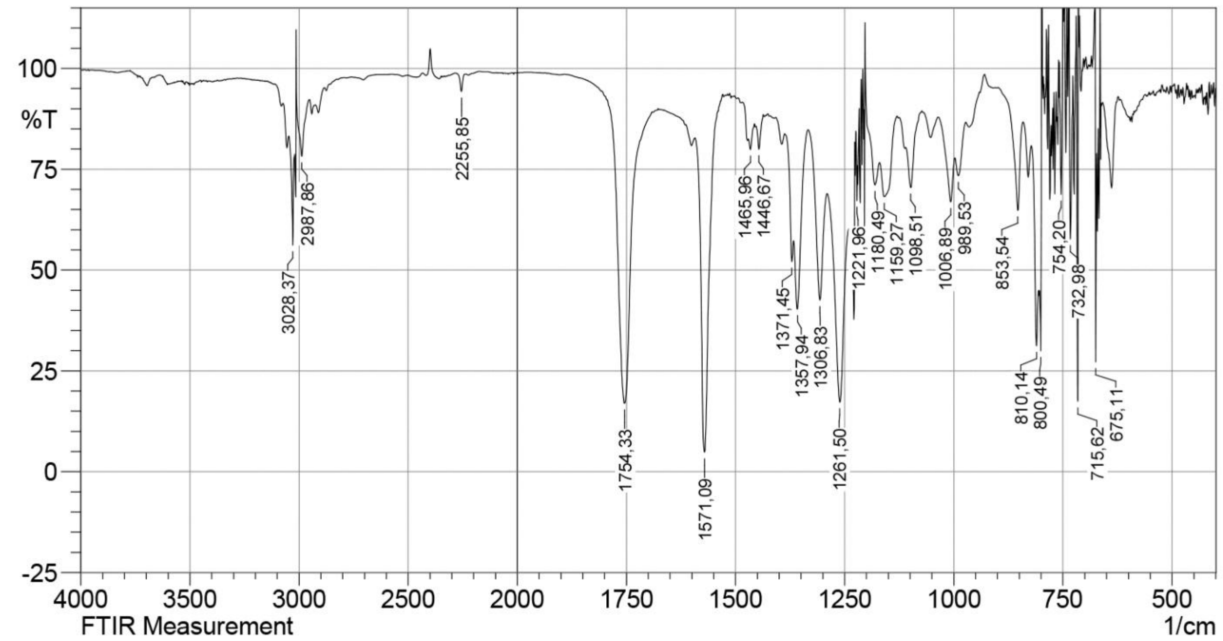

Figure S17. IR spectrum of ethyl 1-cyano-2-nitro-3-(trichloromethyl)cyclopropanecarboxylate (**4**) in $CHCl_3$.

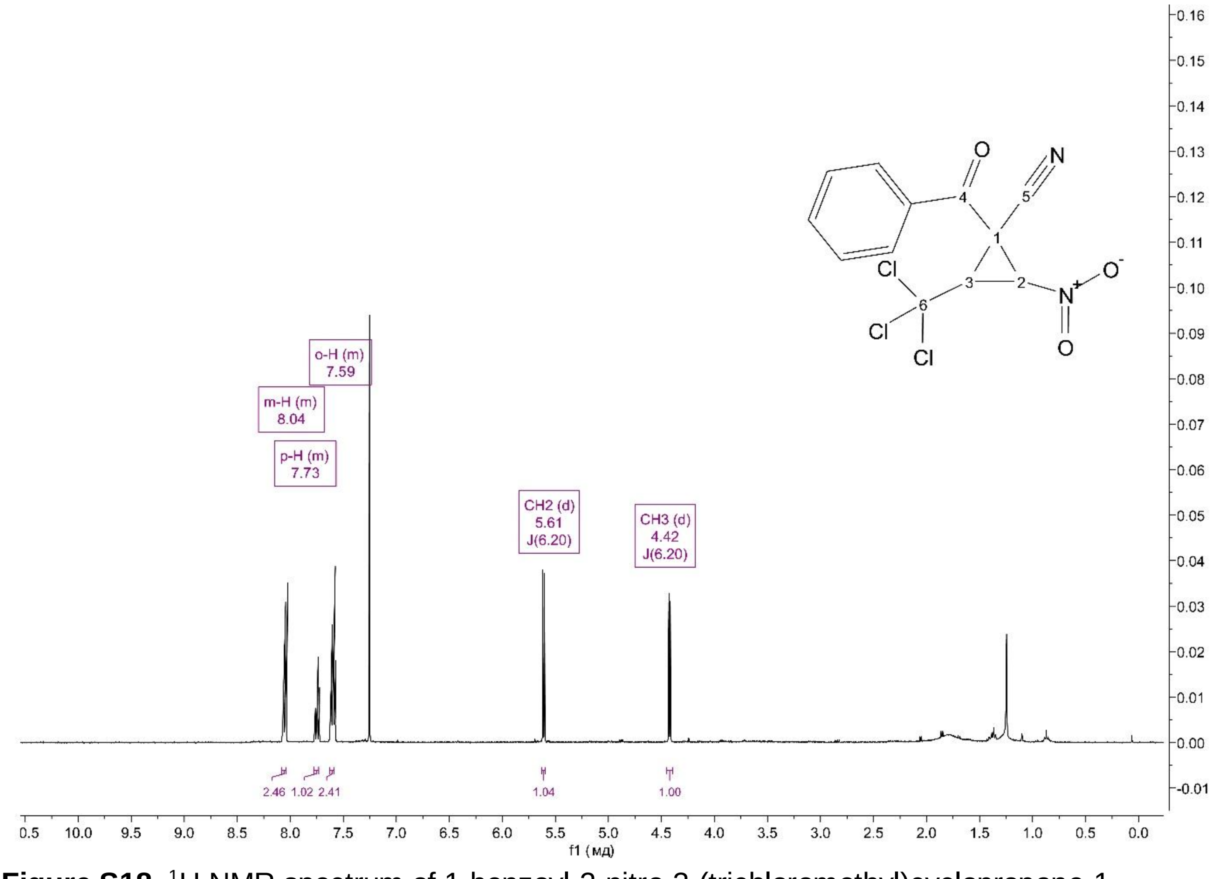

Figure S18. ^{1}H NMR spectrum of 1-benzoyl-2-nitro-3-(trichloromethyl)cyclopropane-1-carbonitrile (**5**) in $CDCl_3$.

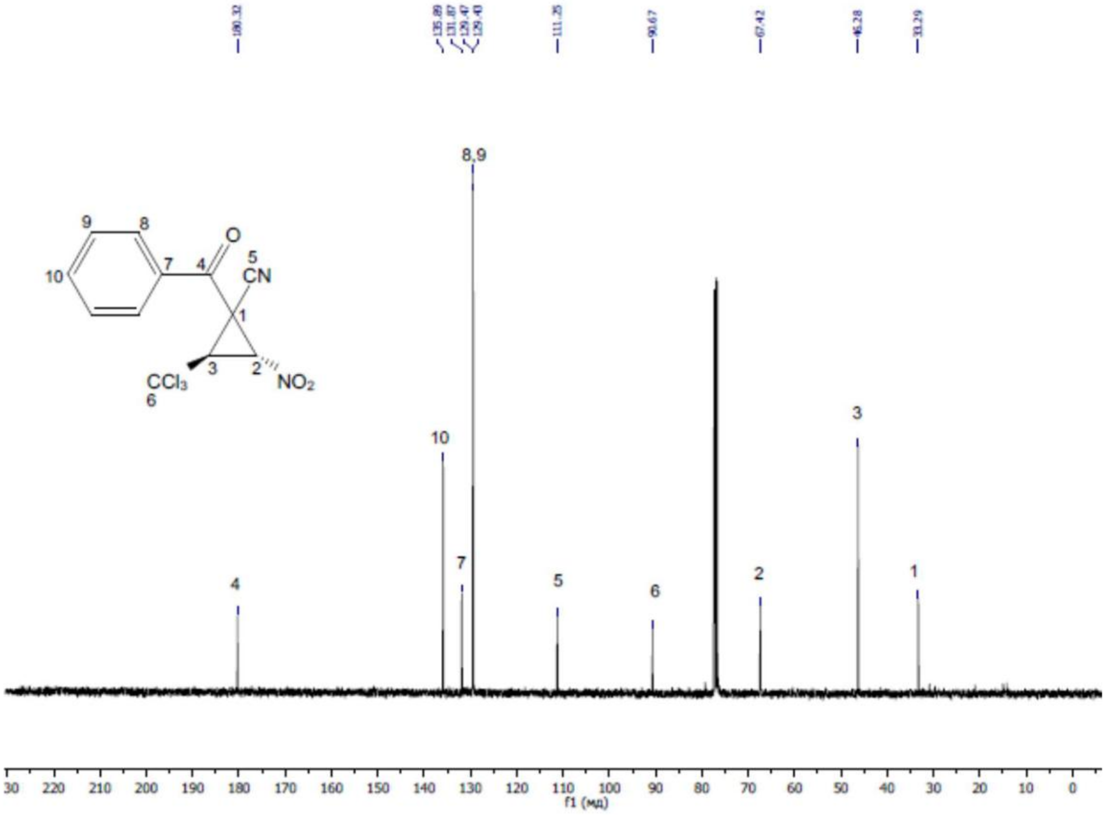

Figure S19. $^{13}C\{^1H\}$ NMR spectrum of 1-benzoyl-2-nitro-3-(trichloromethyl)cyclopropane-1-carbonitrile (**5**) in $CDCl_3$.

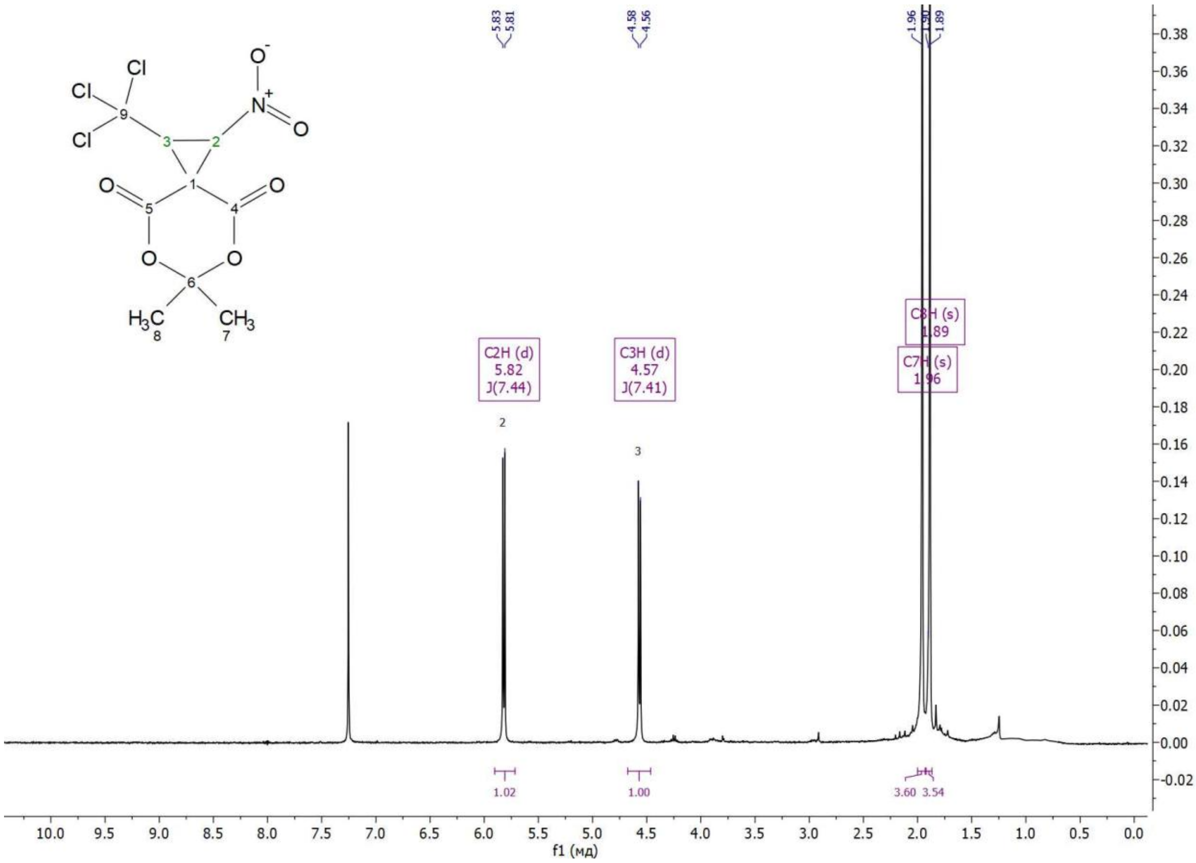

Figure S20. ^1H NMR spectrum of 6,6-dimethyl-1-nitro-2-(trichloromethyl)-5,7-dioxaspiro[2.5]octane-4,8-dione (**6**) in $CDCl_3$.

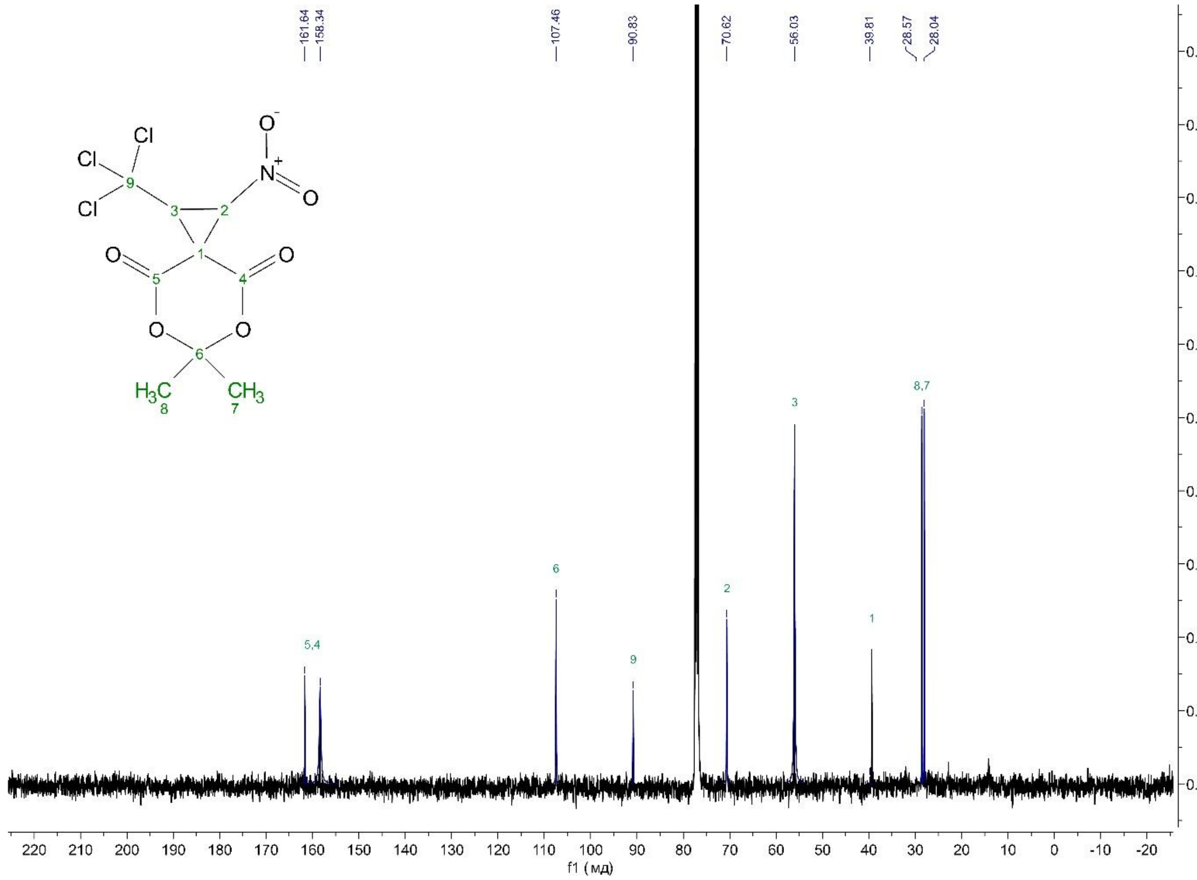

Figure S21. $^{13}C\{^{1}H\}$ NMR spectrum of 6,6-dimethyl-1-nitro-2-(trichloromethyl)-5,7-dioxaspiro[2.5]octane-4,8-dione (**6**) in $CDCl_3$.

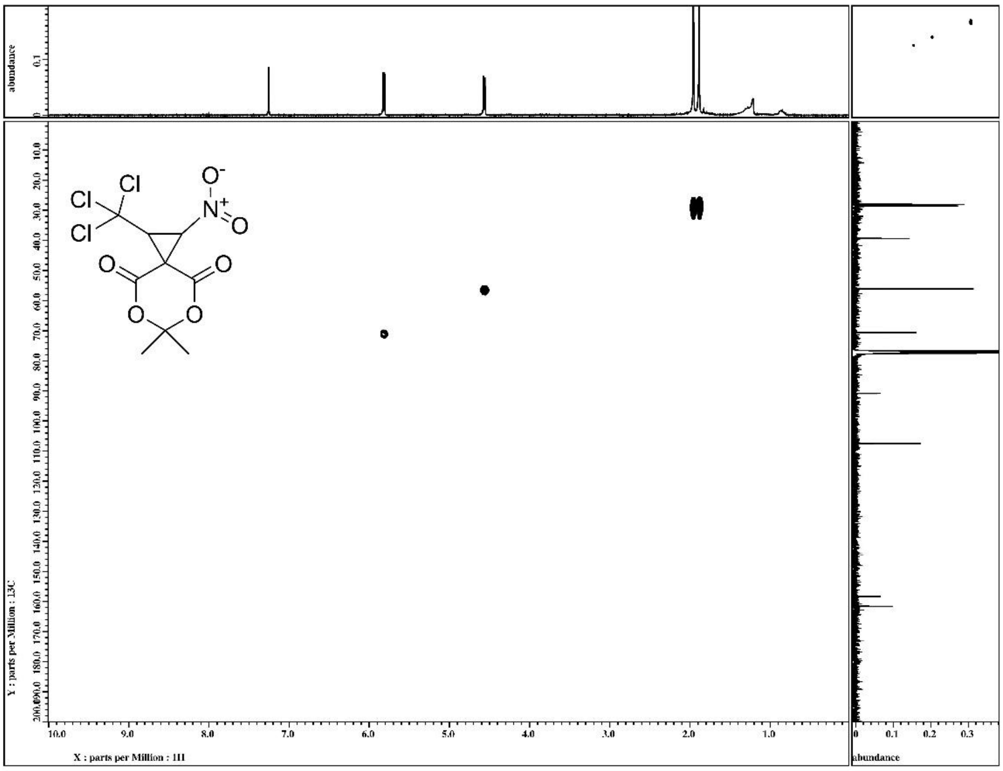

Figure S22. ^{1}H-^{13}C HMQC NMR spectrum of 6,6-dimethyl-1-nitro-2-(trichloromethyl)-5,7-dioxaspiro[2.5]octane-4,8-dione (**6**) in $CDCl_3$.

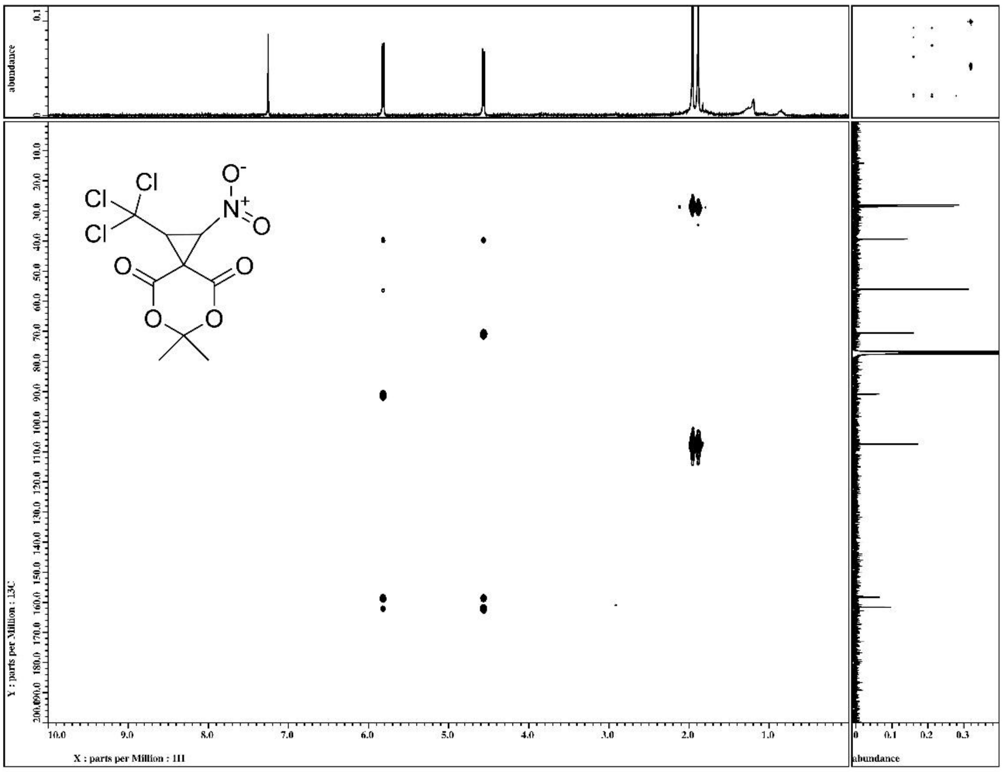

Figure S23. ^1H-^{13}C HMBC NMR spectrum of 6,6-dimethyl-1-nitro-2-(trichloromethyl)-5,7-dioxaspiro[2.5]octane-4,8-dione (**6**) in $CDCl_3$.

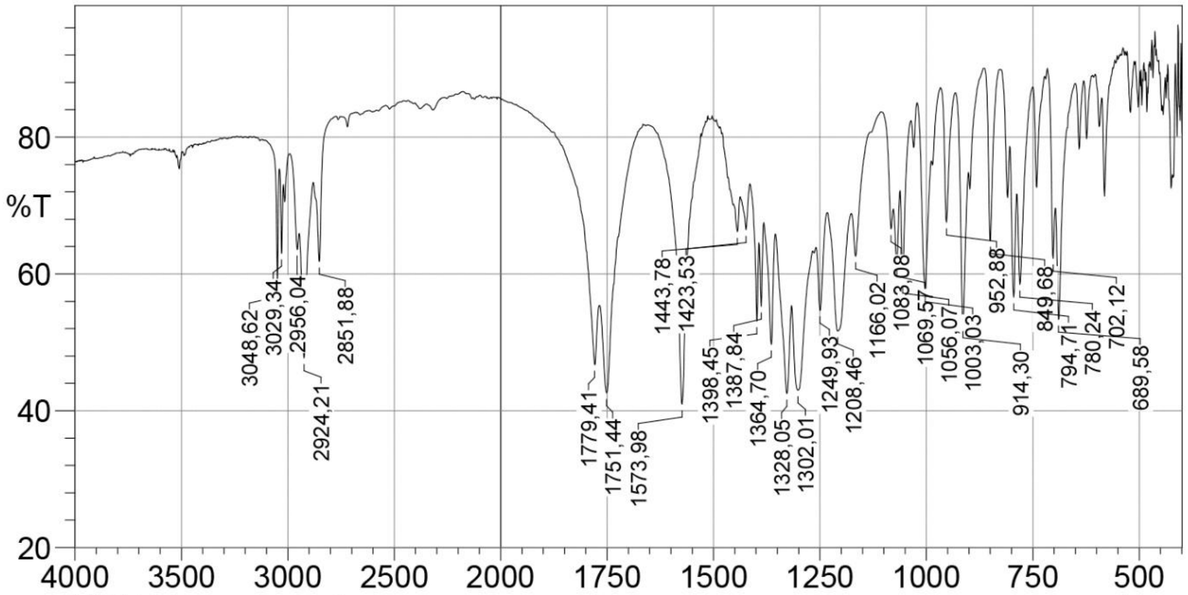

Figure S24. IR spectrum of 6,6-dimethyl-1-nitro-2-(trichloromethyl)-5,7-dioxaspiro[2.5]octane-4,8-dione (**6**) in KBr.

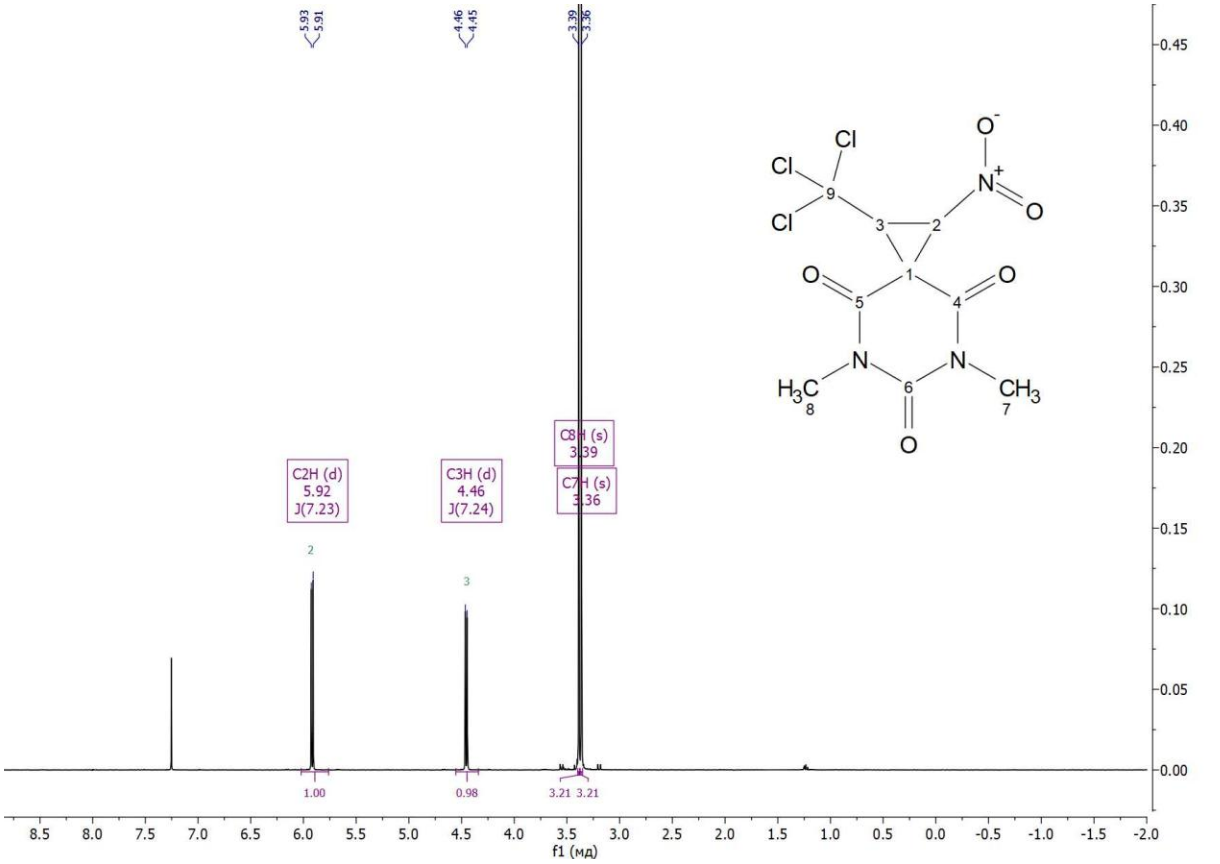

Figure S25. ^{1}H NMR spectrum of 5,7-dimethyl-1-nitro-2-(trichloromethyl)-5,7-diazaspiro[2.5]octane-4,6,8-trione (**7**) in $CDCl_3$.

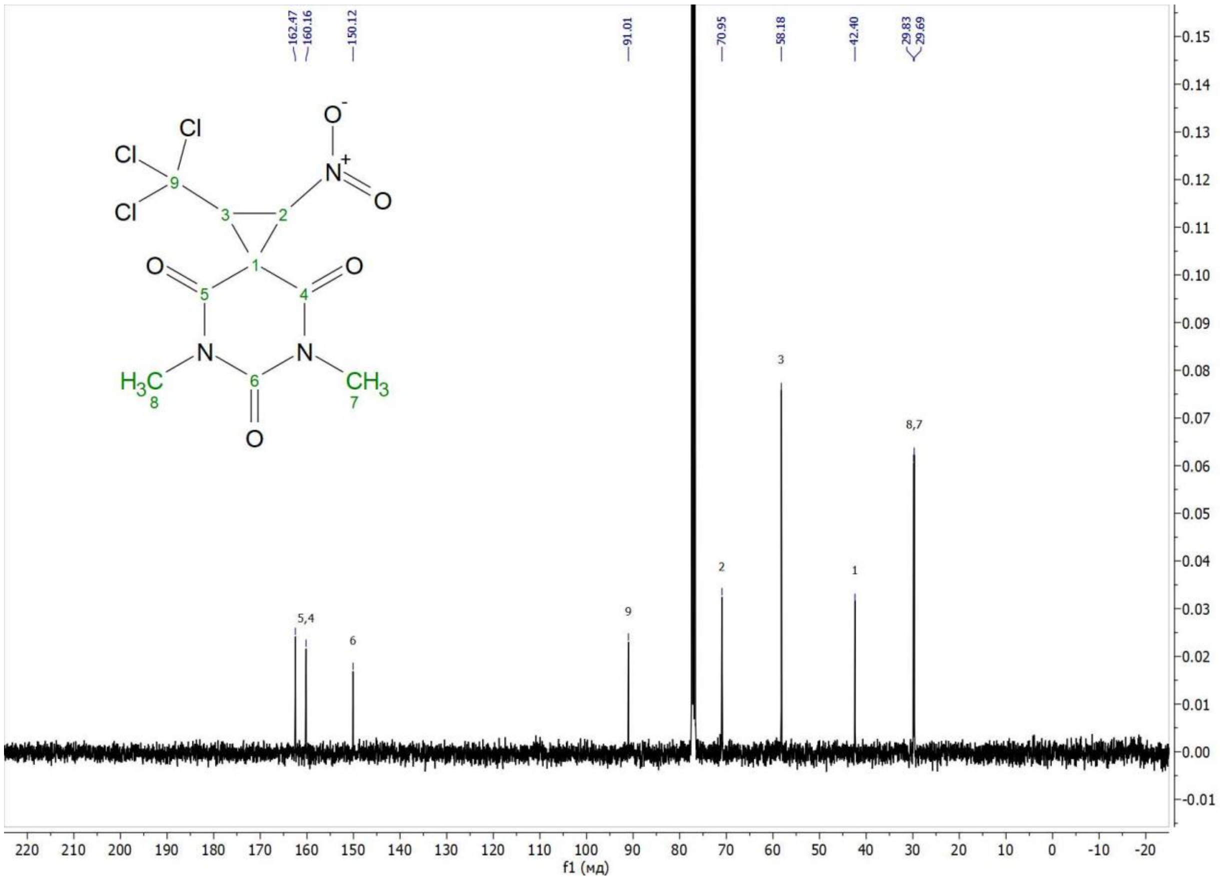

Figure S26. $^{13}C\{^{1}H\}$ NMR spectrum of 5,7-dimethyl-1-nitro-2-(trichloromethyl)-5,7-diazaspiro[2.5]octane-4,6,8-trione (**7**) in $CDCl_3$.

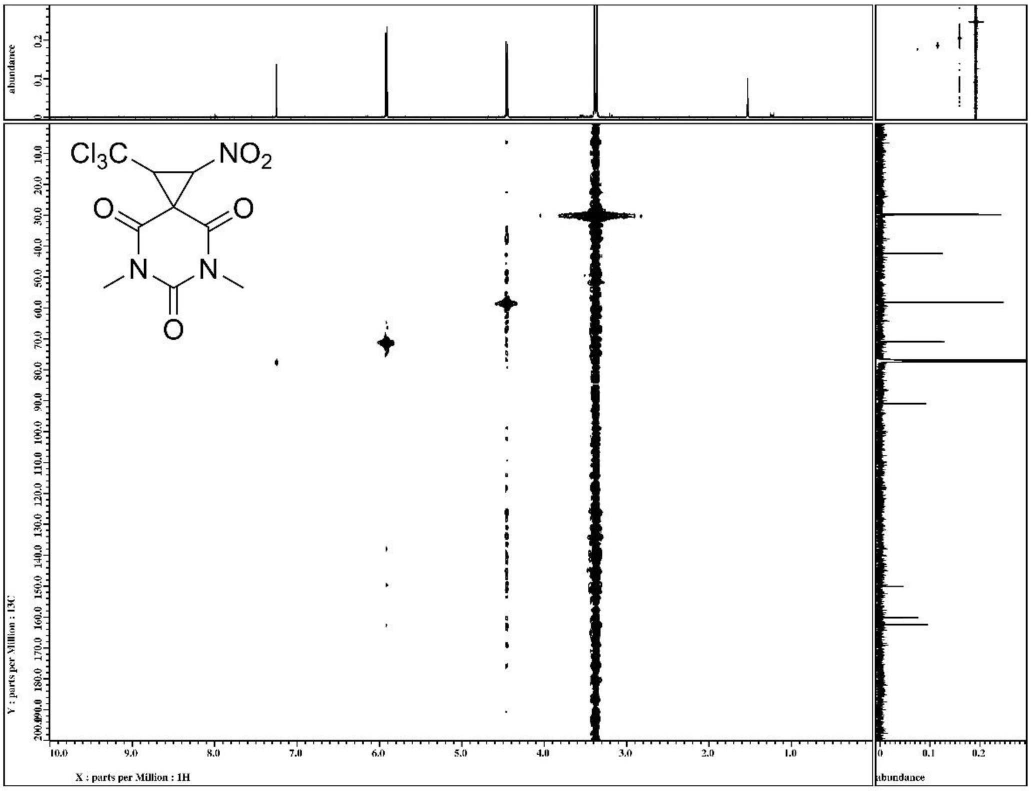

Figure S27. ^{1}H-^{13}C HMQC NMR spectrum of 5,7-dimethyl-1-nitro-2-(trichloromethyl)-5,7-diazaspiro[2.5]octane-4,6,8-trione (**7**) in $CDCl_3$.

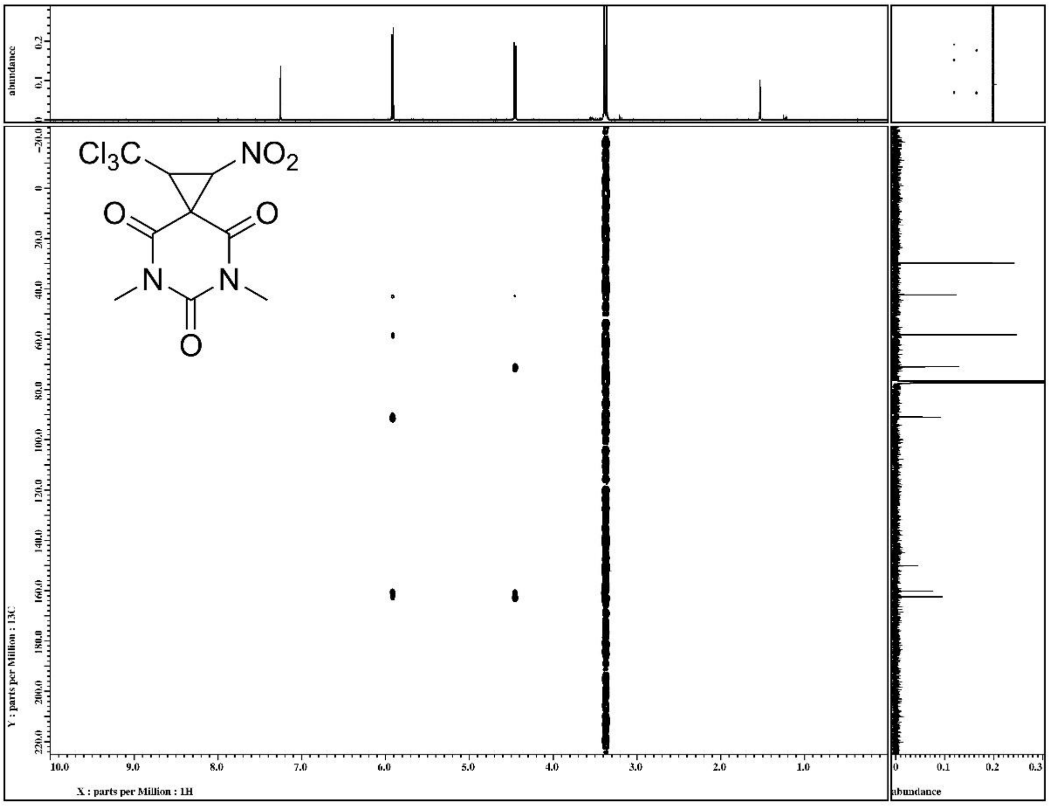

Figure S28. ^{1}H-^{13}C HMBC NMR spectrum of 5,7-dimethyl-1-nitro-2-(trichloromethyl)-5,7-diazaspiro[2.5]octane-4,6,8-trione (**7**) in $CDCl_3$.

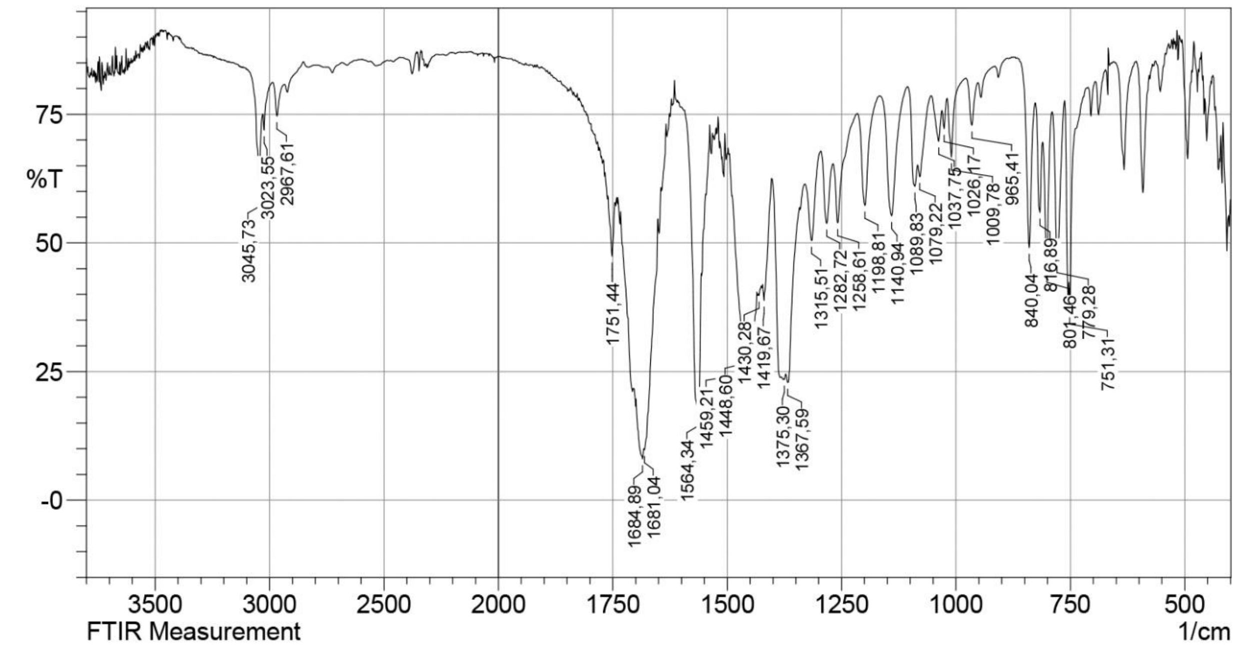

Figure S29. IR spectrum of 5,7-dimethyl-1-nitro-2-(trichloromethyl)-5,7-diazaspiro[2.5]octane-4,6,8-trione (**7**) in KBr.

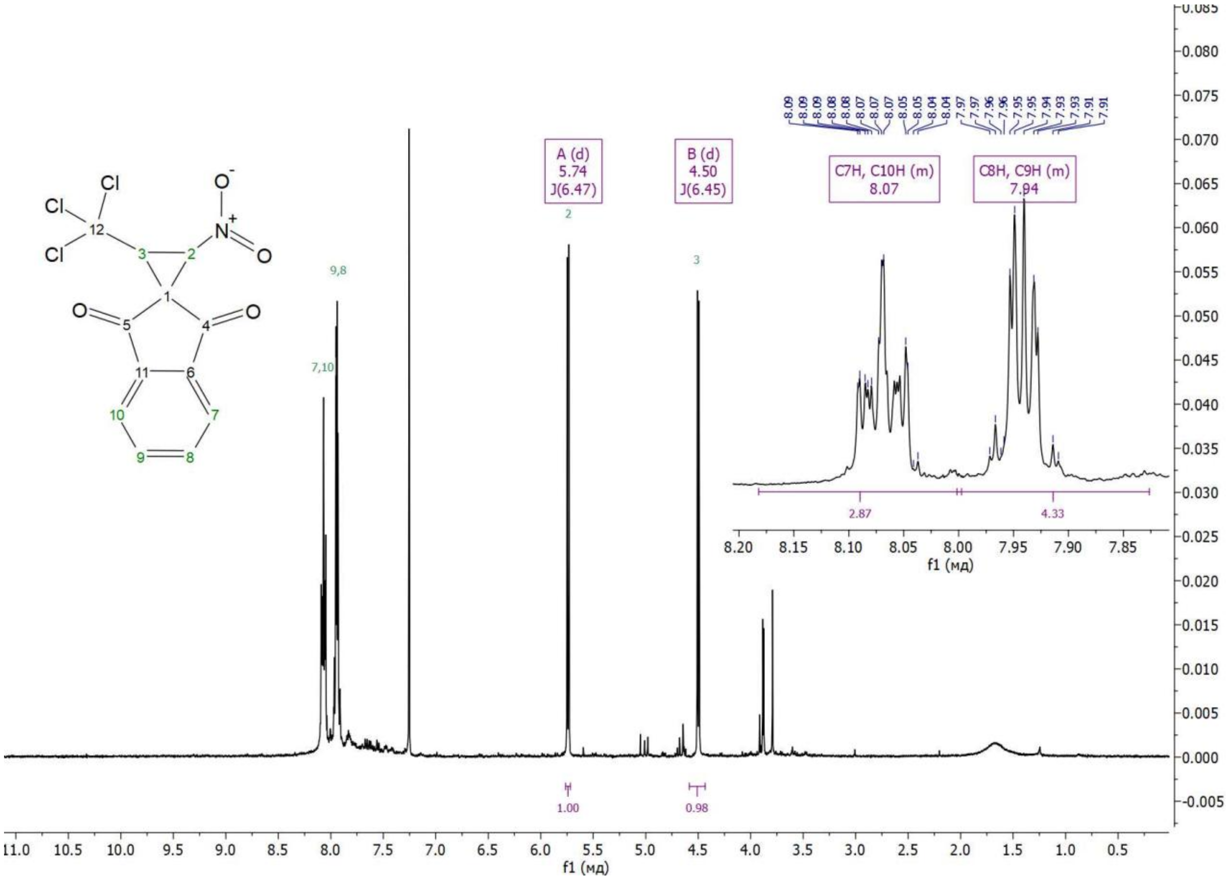

Figure S30. ^{1}H NMR spectrum of 2-nitro-3-(trichloromethyl)spiro[cyclopropane-1,2'-indene]-1',3'-dione (**8**) in $CDCl_3$.

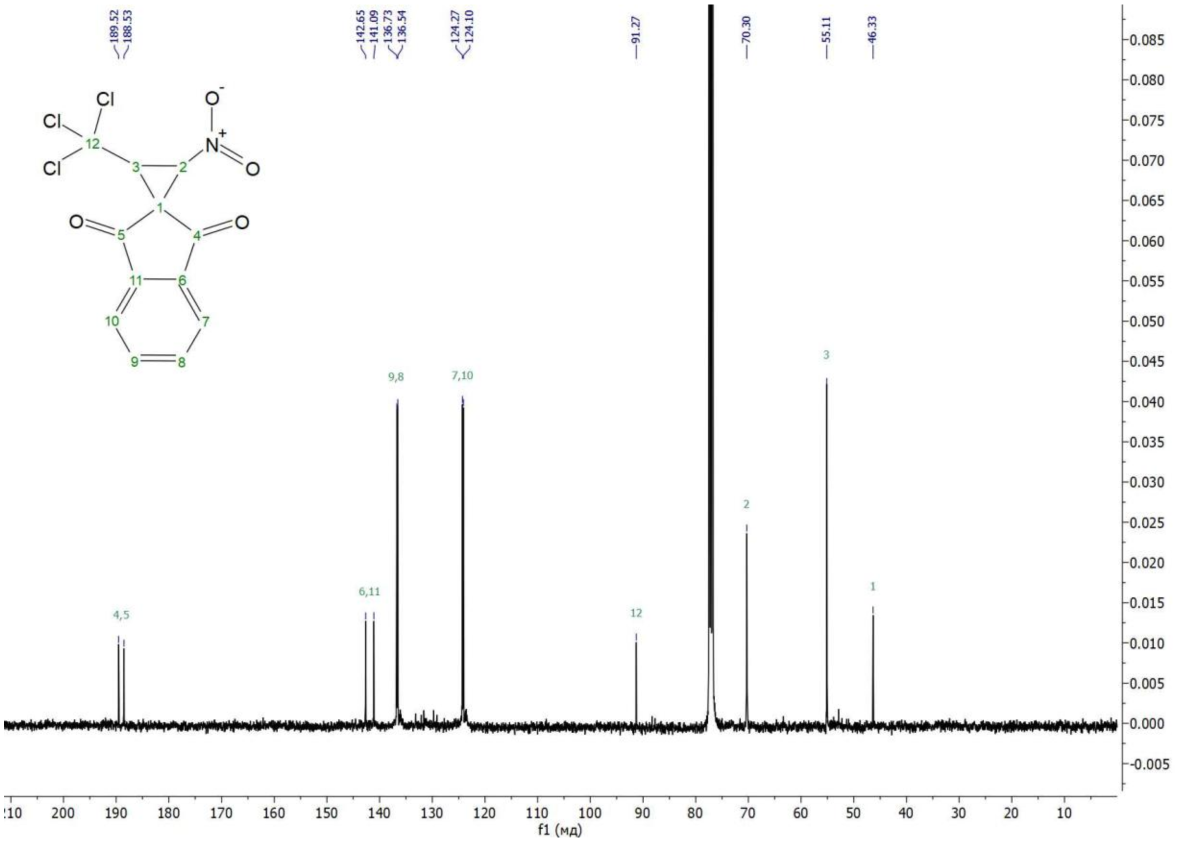

Figure S31. ^{1}H-^{13}C NMR spectrum of 2-nitro-3-(trichloromethyl)spiro[cyclopropane-1,2'-indene]-1',3'-dione (**8**) in $CDCl_3$.

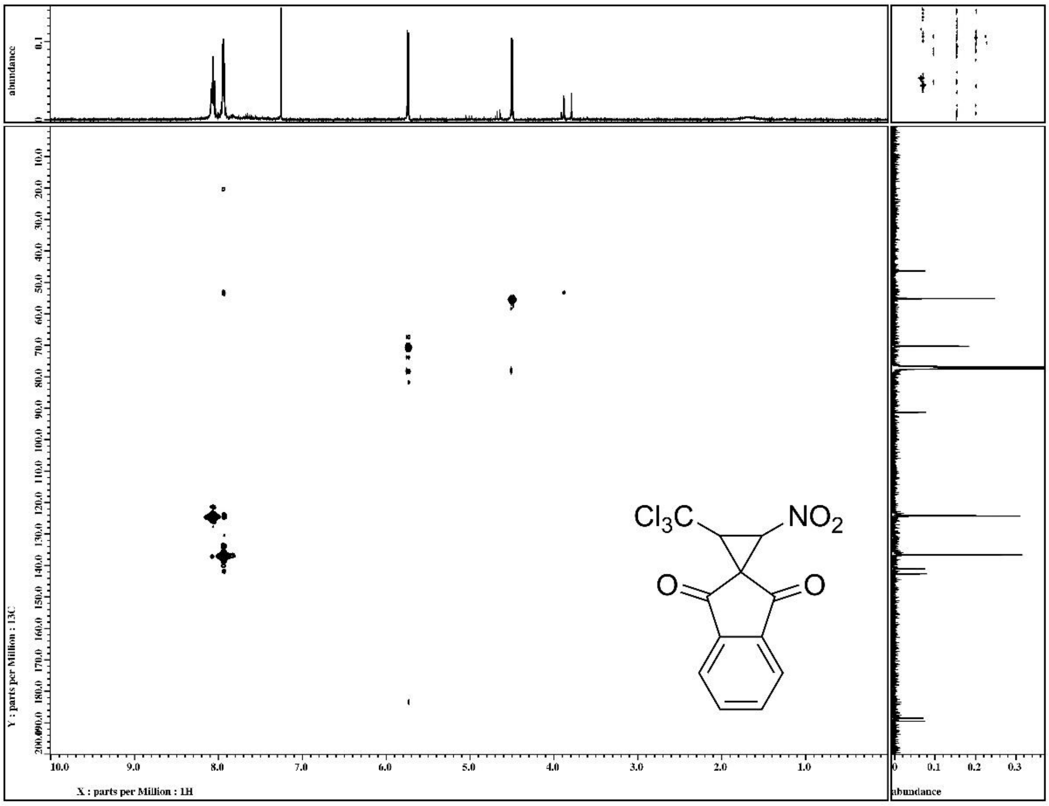

Figure S32. ^{1}H-^{13}C HMQC NMR spectrum of 2-nitro-3-(trichloromethyl)spiro[cyclopropane-1,2'-indene]-1',3'-dione (**8**) in $CDCl_3$.

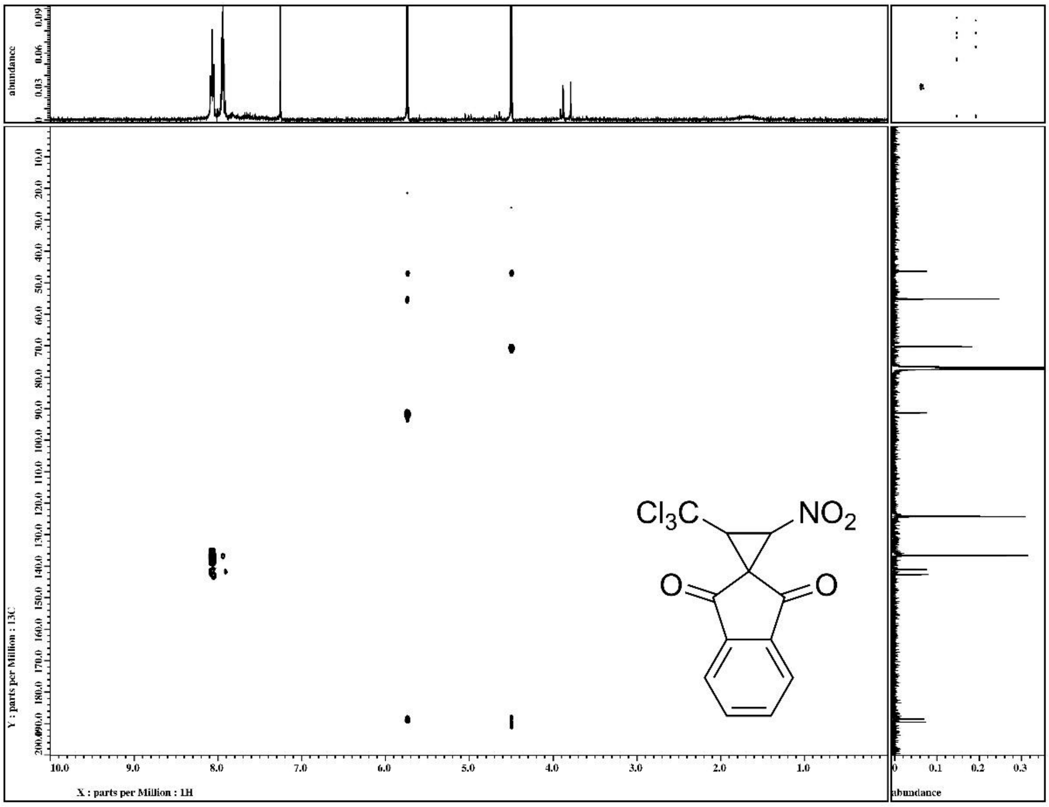

Figure S33. ^{1}H-^{13}C HMBC NMR spectrum of 2-nitro-3-(trichloromethyl)spiro[cyclopropane-1,2'-indene]-1',3'-dione (**8**) in $CDCl_3$.

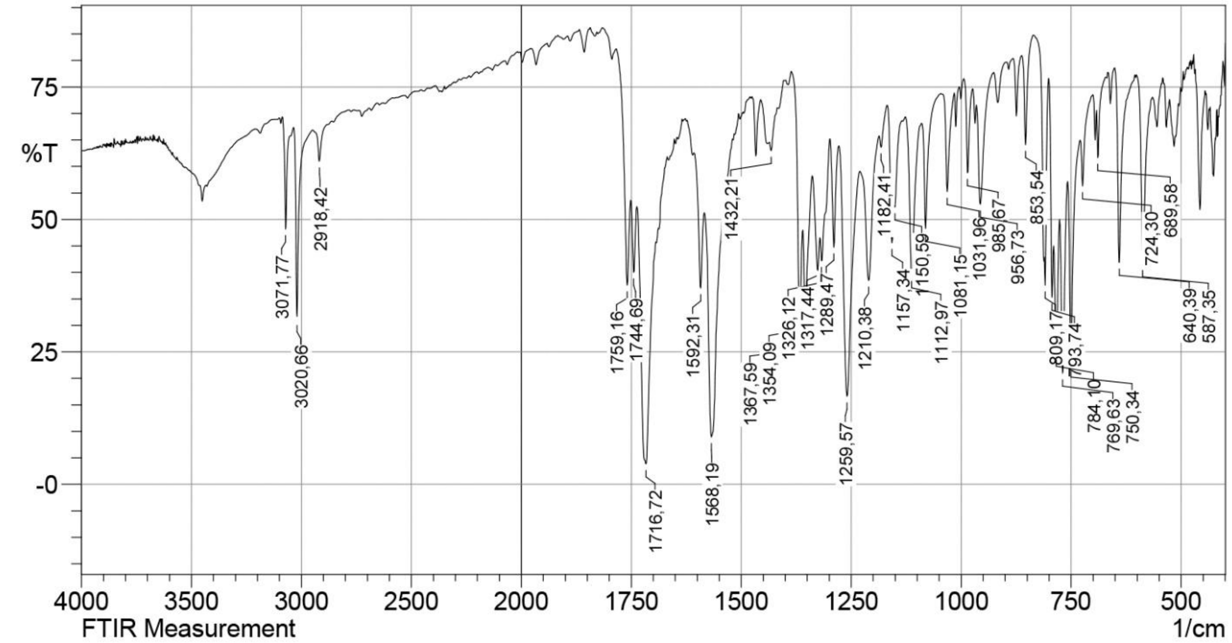

Figure S34. IR spectrum of 2-nitro-3-(trichloromethyl)spiro[cyclopropane-1,2'-indene]-1',3'-dione (**8**) in KBr.

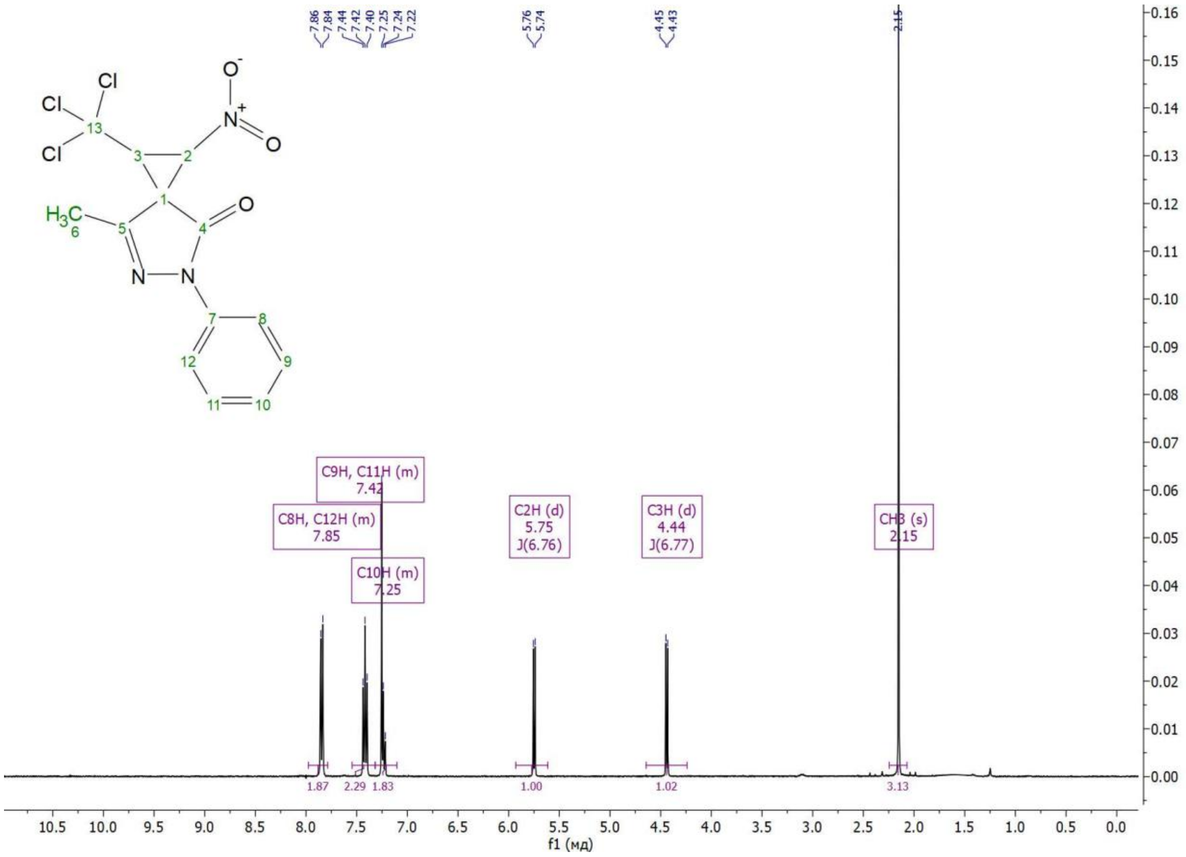

Figure S35. ^{1}H NMR spectrum of 7-methyl-1-nitro-5-phenyl-2-(trichloromethyl)-5,6-diazaspiro[2.4]hept-6-en-4-one (**9a**) in $CDCl_3$.

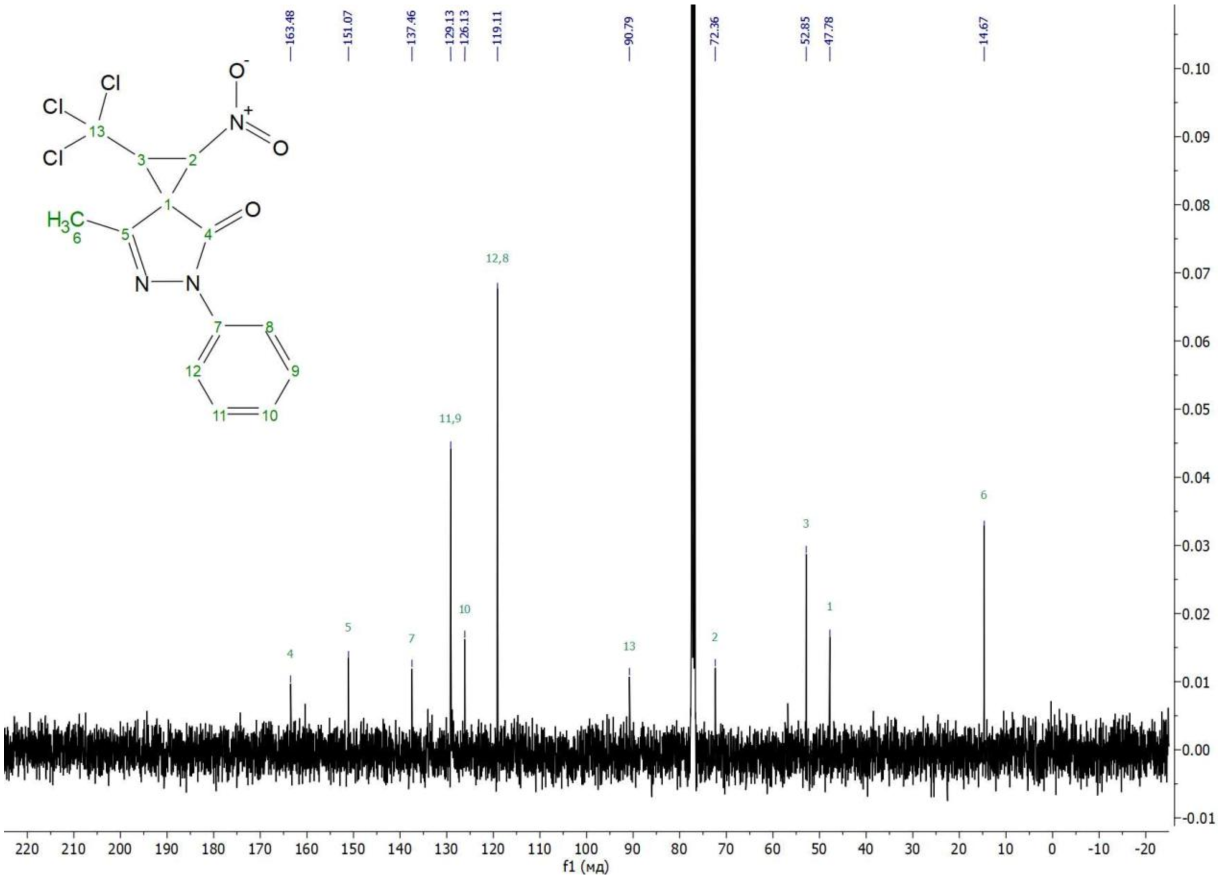

Figure S36. $^{13}C\{^{1}H\}$ NMR spectrum of 7-methyl-1-nitro-5-phenyl-2-(trichloromethyl)-5,6-diazaspiro[2.4]hept-6-en-4-one (**9a**) in $CDCl_3$.

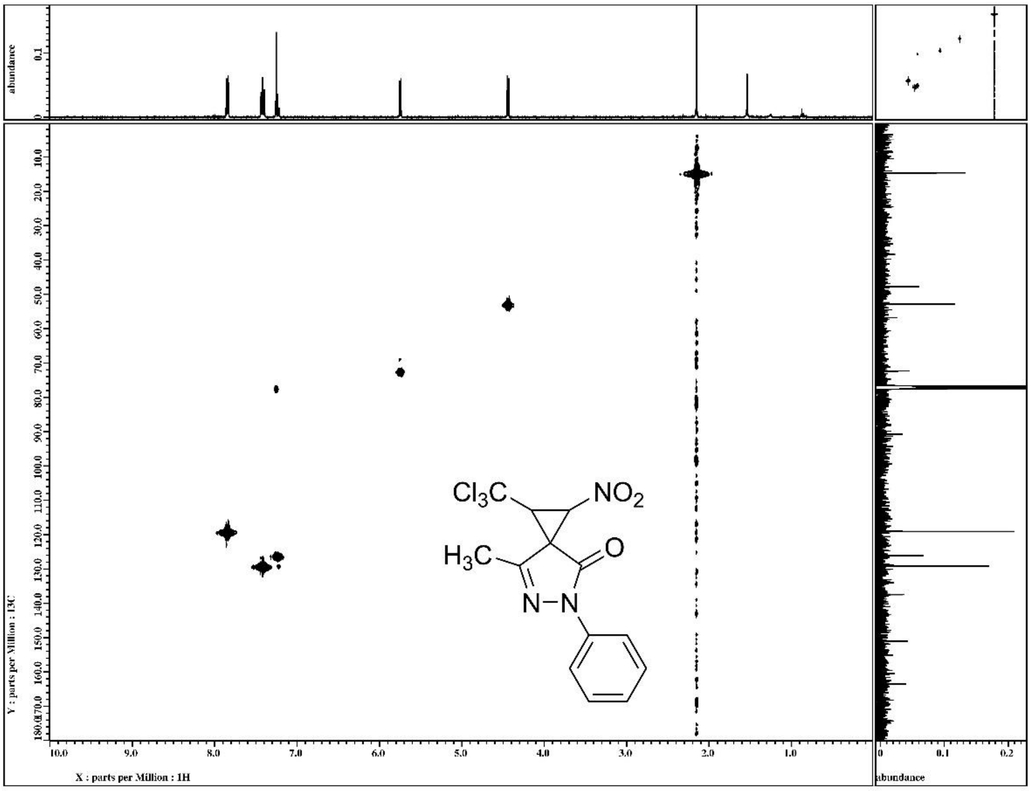

Figure S37. ^{1}H-^{13}C HMQC NMR spectrum of 7-methyl-1-nitro-5-phenyl-2-(trichloromethyl)-5,6-diazaspiro[2.4]hept-6-en-4-one (**9a**) in $CDCl_3$.

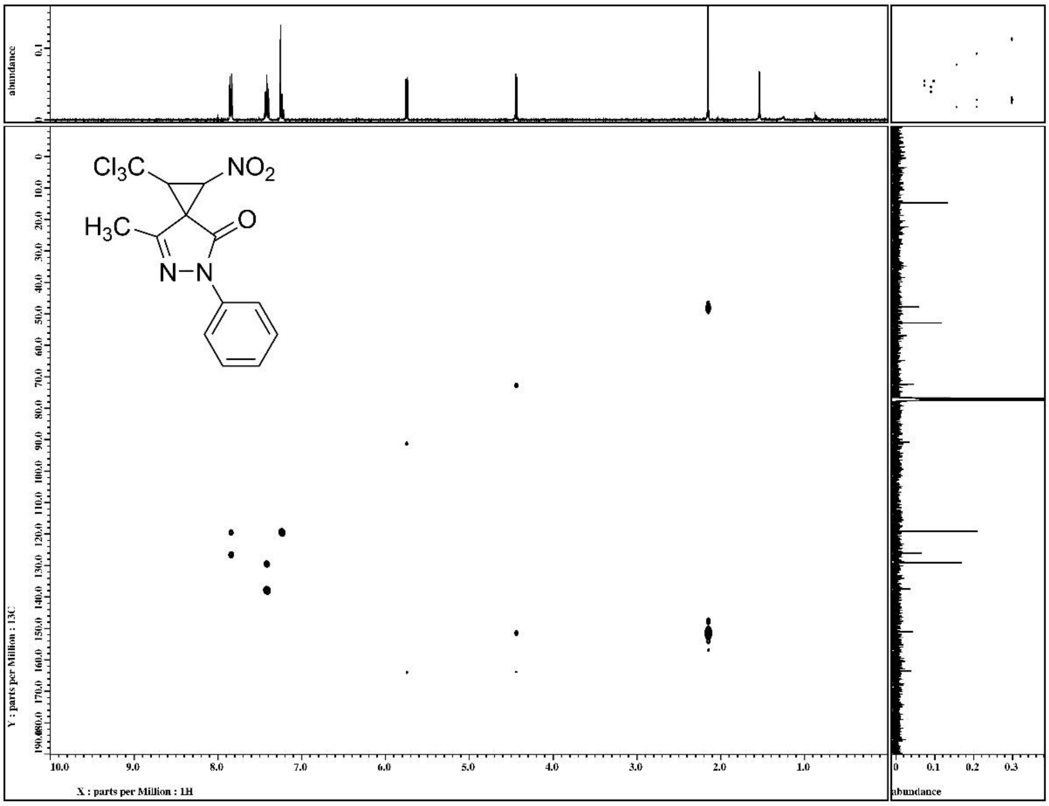

Figure S38. ^{1}H-^{13}C HMBC NMR spectrum of 7-methyl-1-nitro-5-phenyl-2-(trichloromethyl)-5,6-diazaspiro[2.4]hept-6-en-4-one (**9a**) in $CDCl_3$.

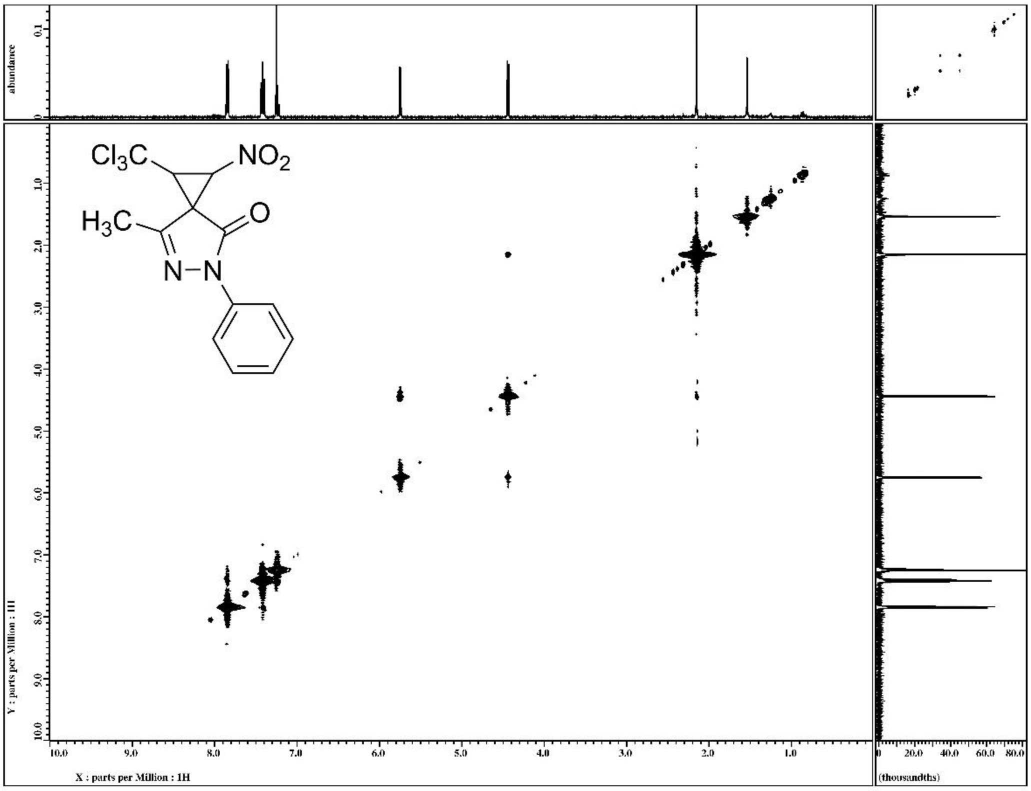

Figure S39. ${}^{1}H$-${}^{1}H$ NOESY NMR spectrum of 7-methyl-1-nitro-5-phenyl-2-(trichloromethyl)-5,6-diazaspiro[2.4]hept-6-en-4-one (**9a**) in $CDCl_3$.

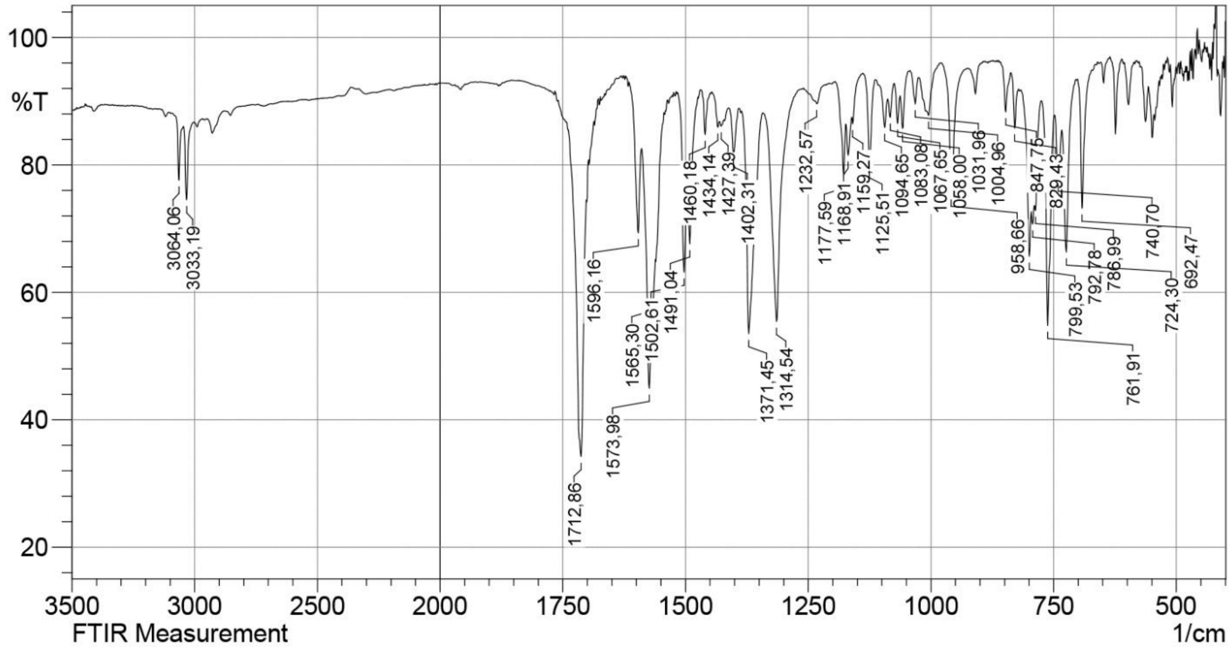

Figure S40. IR spectrum of 7-methyl-1-nitro-5-phenyl-2-(trichloromethyl)-5,6-diazaspiro[2.4]hept-6-en-4-one (**9a**) in KBr.

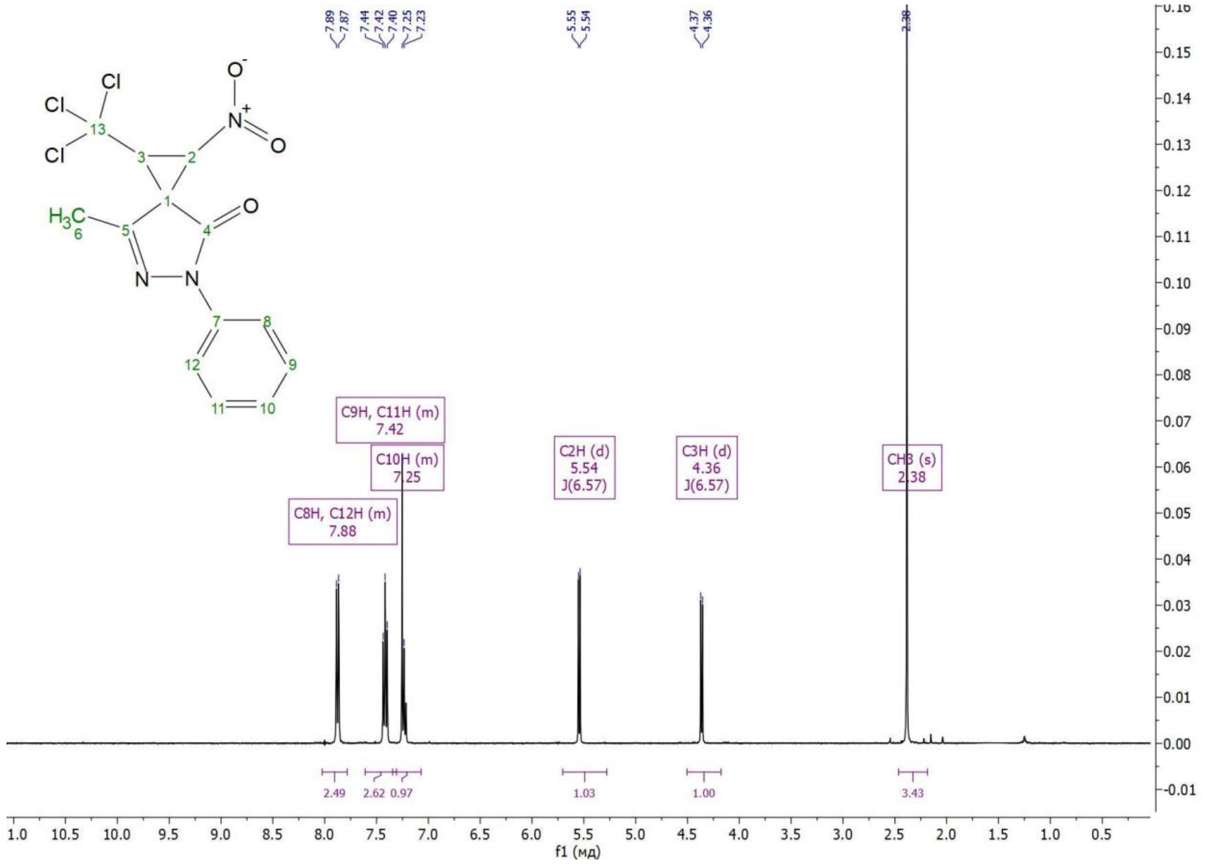

Figure S41. ^{1}H NMR spectrum of 7-methyl-1-nitro-5-phenyl-2-(trichloromethyl)-5,6-diazaspiro[2.4]hept-6-en-4-one (**9b**) in $CDCl_3$.

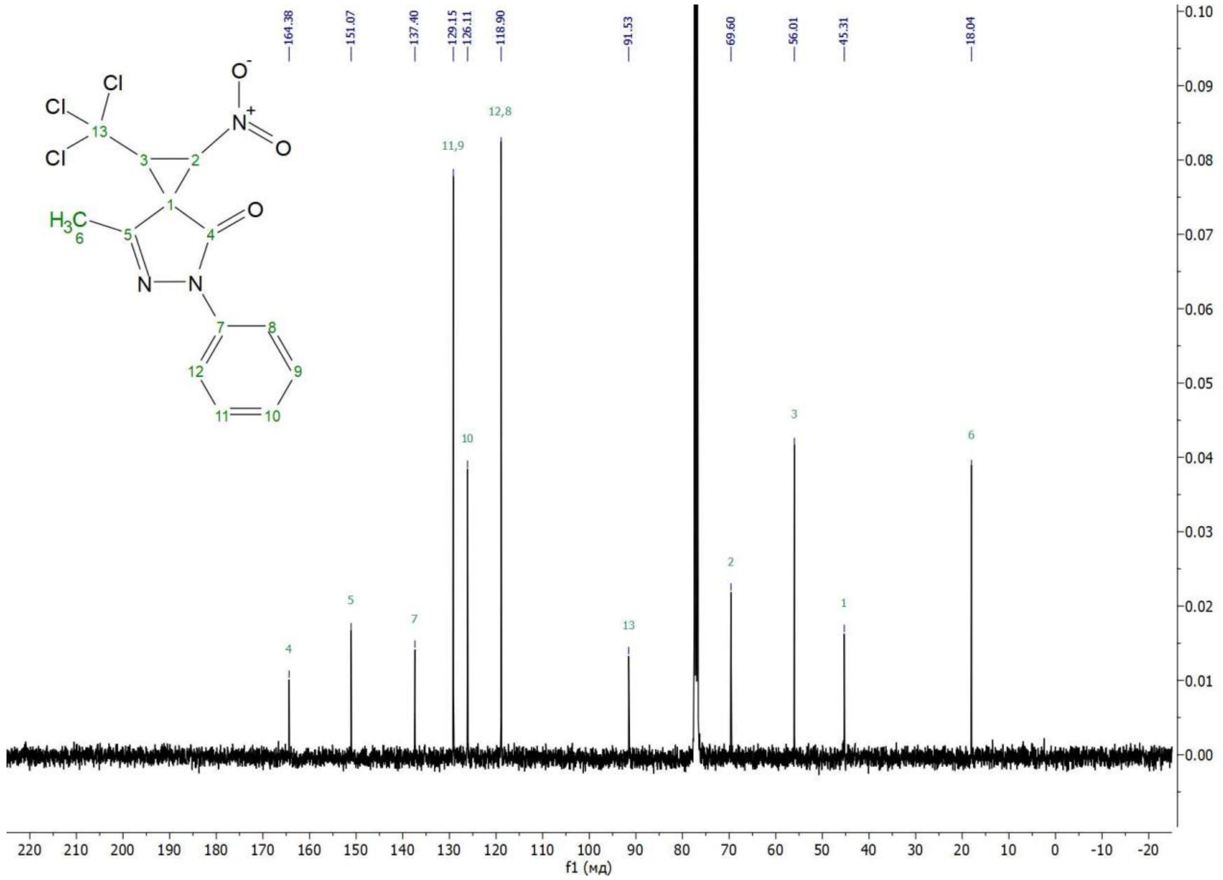

Figure S42. $^{13}C\{^{1}H\}$ NMR spectrum of 7-methyl-1-nitro-5-phenyl-2-(trichloromethyl)-5,6-diazaspiro[2.4]hept-6-en-4-one (**9b**) in $CDCl_3$.

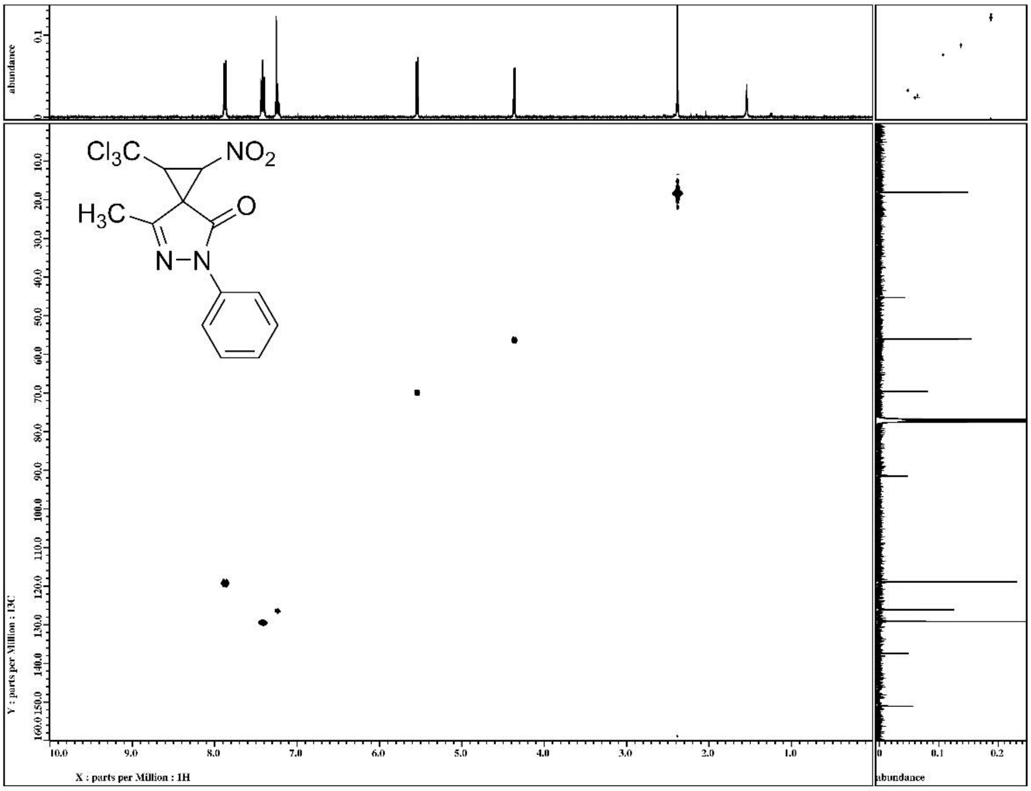

Figure S43. ^{1}H-^{13}C HMQC NMR spectrum of 7-methyl-1-nitro-5-phenyl-2-(trichloromethyl)-5,6-diazaspiro[2.4]hept-6-en-4-one (**9b**) in $CDCl_3$.

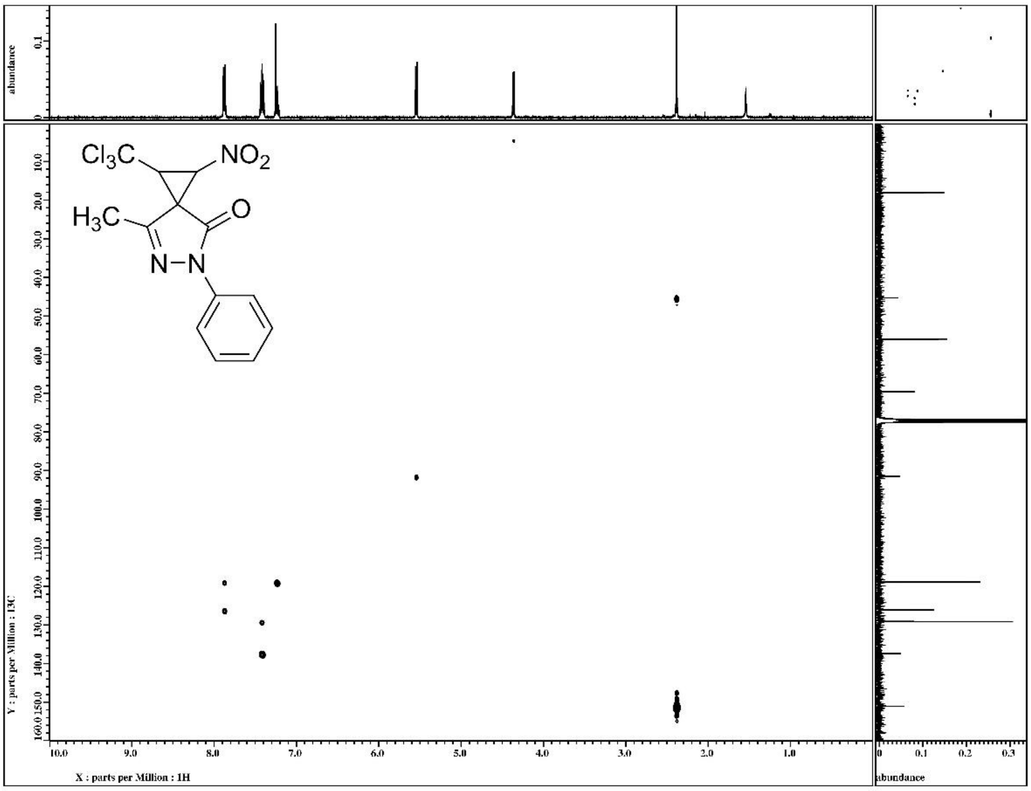

Figure S44. ^{1}H-^{13}C HMBC NMR spectrum of 7-methyl-1-nitro-5-phenyl-2-(trichloromethyl)-5,6-diazaspiro[2.4]hept-6-en-4-one (**9b**) in $CDCl_3$.

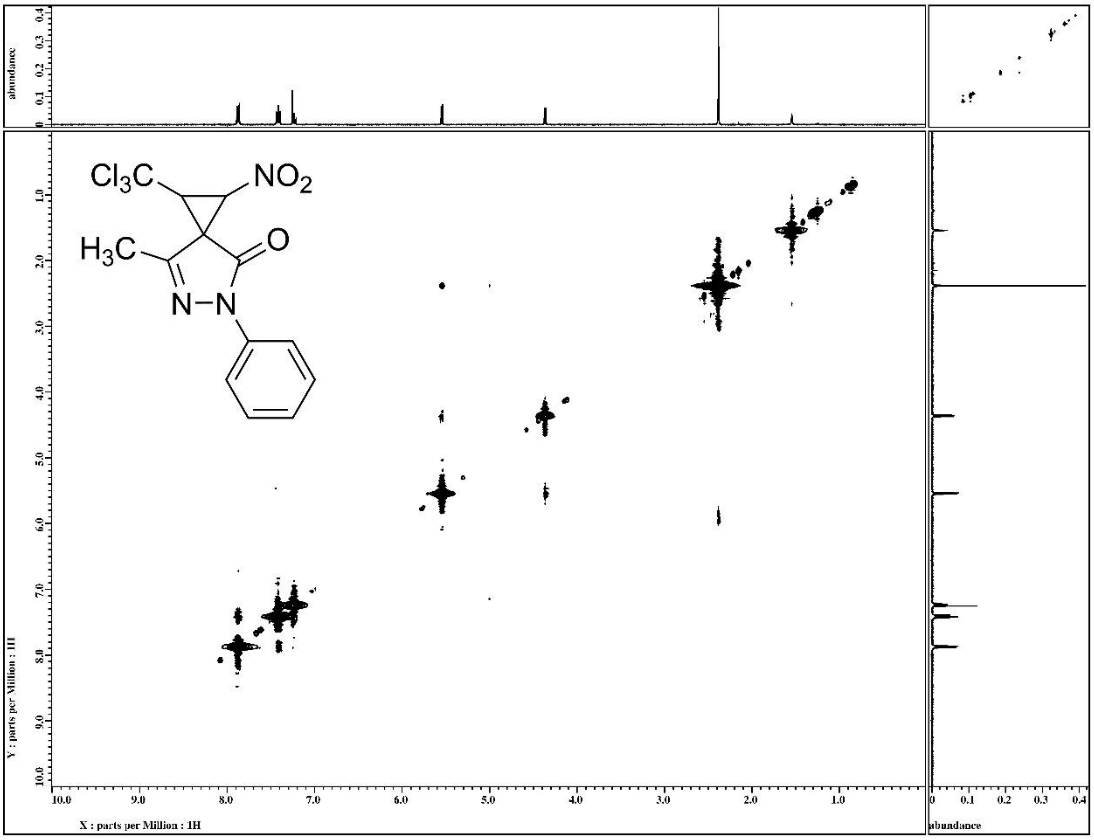

Figure S45. ^1H-^1H NOESY NMR spectrum of 7-methyl-1-nitro-5-phenyl-2-(trichloromethyl)-5,6-diazaspiro[2.4]hept-6-en-4-one (**9b**) in $CDCl_3$.

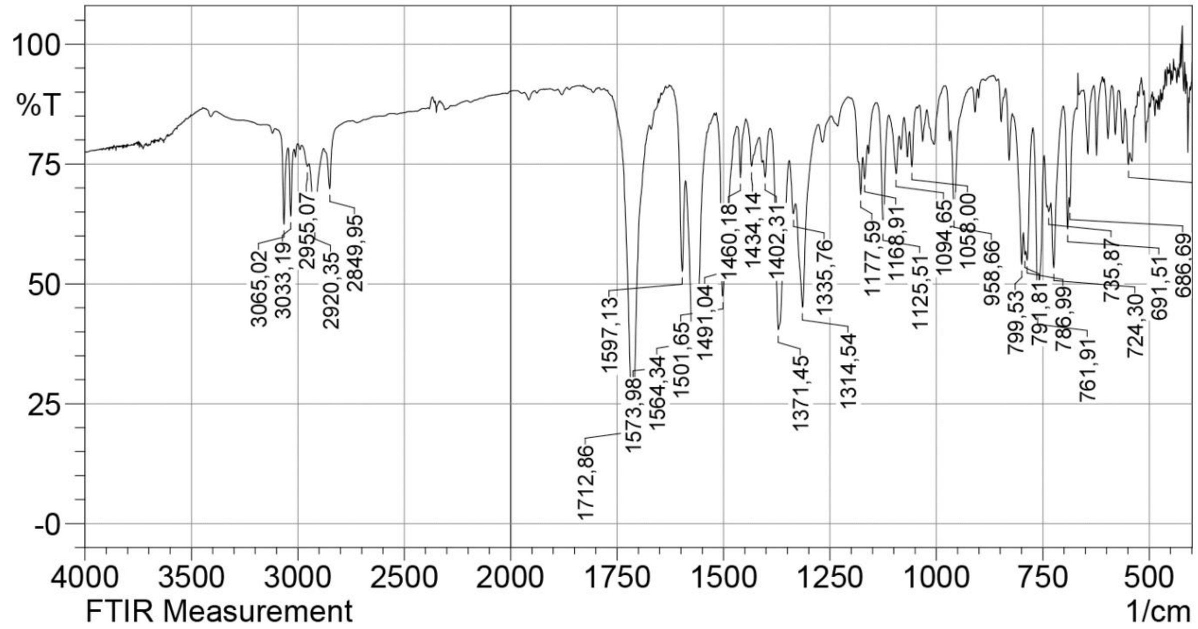

Figure S46. IR spectrum of 7-methyl-1-nitro-5-phenyl-2-(trichloromethyl)-5,6-diazaspiro[2.4]hept-6-en-4-one (**9b**) in KBr.

Table S1. Principal crystallographic parameters of compound **2**, **3**, **7a**, **7b** based on X-ray diffraction data

**Parameter**	**2**	**3**	**9a**	**9b**
Molecular formula	$C_6H_2Cl_3N_3O_2$	$C_7H_5Cl_3N_2O_4$	$C_{13}H_{10}Cl_3N_3O_3$	$C_{13}H_{10}Cl_3N_3O_3$
Molecular weight	254.46	287.48	362.59	362.59
Crystal system	monoclinic	monoclinic	monoclinic	monoclinic
Space group	$P2_1$	$P2_1/n$	$P2_1/n$	$P2_1/n$
Z	4	4	8	8
a/Å	11.6233(2)	8.3468(3)	11.4834(6)	16.6097(2)
b/Å	6.39530(10)	6.1819(2)	11.0564(5)	9.76570(10)
c/Å	13.1586(2)	21.2056(6)	23.7834(10)	19.4325(2)
α/deg	90	90	90	90
β/deg	96.5730(10)	93.234(3)	91.057(4)	102.7570(10)
γ/deg	90	90	90	90
V/Å^3	971.71(3)	1092.45(6)	3019.1(2)	3074.25(6)
d_{calc}/g cm^{-3}	1.739	1.748	1.595	1.567
μ(CuKα)/mm^{-1}	8.393	7.658	5.651	5.550
F(000)	504	576.0	1472	1472
Reflections measured	9591	3763	21046	26692
Independent reflections	3718	2056	6151	6279
R_{int}	0.0391	0.0223	0.0984	0.0567
R_{sigma}	0.0425	0.0260	0.0556	0.0359
GOOF	1.0374	1.087	1.085	1.055
R_1(I≥2σ(I))	0.0262	0.0321	0.0732	0.0395
wR_2(I≥2σ(I))	0.0656	0.0832	0.2101	0.1022
R_1(all data)	0.0267	0.0340	0.0811	0.0414
wR_2	0.0661	0.0844	0.2192	0.0979

* refined as racemic twin, BASF 0.29936, flack parameter 0.299(14)

Table S2. Bond lengths for **2**.

**Atom**	**Atom**	**Length/Å**	**Atom**	**Atom**	**Length/Å**	**Atom**	**Atom**	**Length/Å**
C1A	C2A	1.490(4)	C3A	C4A	1.457(4)	C11A	Cl2A	1.773(3)
C1A	C3A	1.542(4)	C3A	C6A	1.452(5)	C11A	Cl3A	1.763(3)
C1A	N8A	1.475(4)	C3B	C4B	1.449(5)	C11B	Cl1B	1.769(3)
C1B	C2B	1.486(4)	C3B	C6B	1.452(4)	C11B	Cl2B	1.769(3)
C1B	C3B	1.537(4)	C4A	N5A	1.140(5)	C11B	Cl3B	1.774(3)
C1B	N8B	1.474(4)	C4B	N5B	1.152(5)	N8A	O9A	1.220(4)
C2A	C3A	1.534(4)	C6A	N7A	1.143(5)	N8A	O10A	1.225(4)
C2A	C11A	1.517(4)	C6B	N7B	1.145(4)	N8B	O9B	1.223(4)
C2B	C3B	1.535(4)	C11A	Cl1A	1.779(3)	N8B	O10B	1.223(4)
C2B	C11B	1.527(4)						

Table S3. Bond angles for **2**.

**Atom**	**Atom**	**Atom**	**Angle/˚**	**Atom**	**Atom**	**Atom**	**Angle/˚**	**Atom**	**Atom**	**Atom**	**Angle/˚**
C2A	C1A	C3A	60.75(19)	C6B	C3B	C2B	115.4(3)	Cl2A	C11A	Cl1A	109.89(17)
N8A	C1A	C2A	118.6(3)	N5A	C4A	C3A	177.3(3)	Cl3A	C11A	Cl1A	109.44(17)
N8A	C1A	C3A	117.4(3)	N5B	C4B	C3B	179.2(4)	Cl3A	C11A	Cl2A	108.59(16)
C2B	C1B	C3B	61.0(2)	N7A	C6A	C3A	177.2(3)	C2B	C11B	Cl1B	107.8(2)
N8B	C1B	C2B	118.7(3)	N7B	C6B	C3B	178.2(3)	C2B	C11B	Cl2B	109.0(2)
N8B	C1B	C3B	118.2(2)	C2A	C11A	Cl1A	107.9(2)	C2B	C11B	Cl3B	112.4(2)
C1A	C2A	C3A	61.29(19)	C2A	C11A	Cl2A	107.7(2)	Cl1B	C11B	Cl3B	109.69(18)
C1A	C2A	C11A	122.6(3)	C2A	C11A	Cl3A	113.3(2)	Cl2B	C11B	Cl1B	109.07(16)
C11A	C2A	C3A	122.1(3)	C6A	C3A	C2A	122.7(3)	Cl2B	C11B	Cl3B	108.82(18)
C1B	C2B	C3B	61.15(19)	C6A	C3A	C4A	113.1(3)	O9A	N8A	C1A	116.1(3)
C1B	C2B	C11B	123.2(3)	C2B	C3B	C1B	57.86(18)	O9A	N8A	O10A	126.0(3)
C11B	C2B	C3B	121.8(3)	C4B	C3B	C1B	115.5(3)	O10A	N8A	C1A	118.0(3)
C2A	C3A	C1A	57.96(19)	C4B	C3B	C2B	121.0(3)	O9B	N8B	C1B	116.6(3)
C4A	C3A	C1A	120.1(3)	C4B	C3B	C6B	115.3(3)	O10B	N8B	C1B	118.2(2)
C4A	C3A	C2A	115.4(3)	C6B	C3B	C1B	120.0(3)	O10B	N8B	O9B	125.2(3)
C6A	C3A	C1A	117.1(3)								

Table S4. Torsion angles for **2**.

A	B	C	D	Angle/˚	A	B	C	D	Angle/˚
C1A	C2A	C3A	C4A	-111.0(3)	C3A	C2A	C11A	Cl3A	54.0(3)
C1A	C2A	C3A	C6A	103.6(3)	C3B	C1B	C2B	C11B	111.0(3)
C1A	C2A	C11A	Cl1A	-141.6(2)	C3B	C1B	N8B	O9B	-130.9(3)
C1A	C2A	C11A	Cl2A	99.9(3)	C3B	C1B	N8B	O10B	49.4(4)
C1A	C2A	C11A	Cl3A	-20.3(4)	C3B	C2B	C11B	Cl1B	-59.3(3)
C1B	C2B	C3B	C4B	102.4(3)	C3B	C2B	C11B	Cl2B	-177.5(2)
C1B	C2B	C3B	C6B	-110.8(3)	C3B	C2B	C11B	Cl3B	61.8(3)
C1B	C2B	C11B	Cl1B	-133.4(3)	C11A	C2A	C3A	C1A	-112.5(3)
C1B	C2B	C11B	Cl2B	108.3(3)	C11A	C2A	C3A	C4A	136.5(3)
C1B	C2B	C11B	Cl3B	-12.4(4)	C11A	C2A	C3A	C6A	-8.8(4)
C2A	C1A	C3A	C4A	102.9(3)	C11B	C2B	C3B	C1B	-113.2(3)
C2A	C1A	C3A	C6A	-113.4(3)	C11B	C2B	C3B	C4B	-10.8(4)
C2A	C1A	N8A	O9A	148.3(3)	C11B	C2B	C3B	C6B	136.0(3)
C2A	C1A	N8A	O10A	-32.6(4)	N8A	C1A	C2A	C3A	107.2(3)
C2B	C1B	C3B	C4B	-112.0(3)	N8A	C1A	C2A	C11A	-141.2(3)
C2B	C1B	C3B	C6B	102.8(3)	N8A	C1A	C3A	C2A	-109.2(3)
C2B	C1B	N8B	O9B	158.6(3)	N8A	C1A	C3A	C4A	-6.3(4)
C2B	C1B	N8B	O10B	-21.1(4)	N8A	C1A	C3A	C6A	137.5(3)
C3A	C1A	C2A	C11A	111.6(3)	N8B	C1B	C2B	C3B	108.3(3)
C3A	C1A	N8A	O9A	-141.8(3)	N8B	C1B	C2B	C11B	-140.7(3)
C3A	C1A	N8A	O10A	37.3(4)	N8B	C1B	C3B	C2B	-108.9(3)
C3A	C2A	C11A	Cl1A	-67.4(3)	N8B	C1B	C3B	C4B	139.1(3)
C3A	C2A	C11A	Cl2A	174.1(2)	N8B	C1B	C3B	C6B	-6.1(4)

Table S5. Bond lengths for **3**.

Atom	Atom	Length/Å	Atom	Atom	Length/Å	Atom	Atom	Length/Å
C1	C2	1.479(3)	C6	O6	1.202(3)	C11	Cl1	1.766(2)
C1	C3	1.526(3)	C6	O7	1.314(2)	C11	Cl2	1.7780(19)
C1	N8	1.486(2)	C7	O7	1.454(2)	C11	Cl3	1.775(2)
C2	C3	1.533(3)	C3	C6	1.519(3)	N8	O9	1.217(2)
C2	C11	1.512(3)	C4	N5	1.146(3)	N8	O10	1.218(2)
C3	C4	1.448(3)						

Table S6. Bond angles for **3**.

**Atom**	**Atom**	**Atom**	**Angle/˚**	**Atom**	**Atom**	**Atom**	**Angle/˚**	**Atom**	**Atom**	**Atom**	**Angle/˚**
C2	C1	C3	61.34(12)	O6	C6	C3	122.07(17)	C2	C11	Cl3	109.18(14)
C2	C1	N8	119.27(16)	O6	C6	O7	126.69(18)	Cl1	C11	Cl2	109.90(11)
N8	C1	C3	117.22(16)	O7	C6	C3	111.14(16)	Cl1	C11	Cl3	108.71(11)
C1	C2	C3	60.84(12)	C2	C11	Cl1	113.04(14)	Cl3	C11	Cl2	108.53(10)
C1	C2	C11	121.63(17)	C2	C11	Cl2	107.39(13)	O9	N8	C1	116.42(16)
C11	C2	C3	125.05(16)	C6	C3	C1	115.79(16)	O9	N8	O10	125.95(18)
C1	C3	C2	57.82(12)	C6	C3	C2	113.13(16)	O10	N8	C1	117.56(17)
C4	C3	C1	117.71(16)	N5	C4	C3	177.1(2)	C6	O7	C7	114.56(15)
C4	C3	C2	123.80(17)	C4	C3	C6	115.99(16)				

Table S7. Torsion angles for **3**.

**A**	**B**	**C**	**D**	**Angle/˚**	**A**	**B**	**C**	**D**	**Angle/˚**	**A**	**B**	**C**	**D**	**Angle/˚**
C1	C2	C3	C4	-103.9(2)	C3	C2	C11	Cl1	-47.3(2)	C4	C3	C6	O7	23.5(2)
C1	C2	C3	C6	106.87(17)	C3	C2	C11	Cl2	-168.73(15)	C11	C2	C3	C1	110.0(2)
C1	C2	C11	Cl1	27.2(2)	C3	C2	C11	Cl3	73.8(2)	C11	C2	C3	C4	6.1(3)
C1	C2	C11	Cl2	-94.22(18)	C3	C6	O7	C7	177.32(15)	C11	C2	C3	C6	-143.12(18)
C1	C2	C11	Cl3	148.30(15)	C4	C3	C6	O6	-159.86(18)	N8	C1	C2	C3	-106.93(19)
C1	C3	C6	O6	56.0(2)	C2	C3	C6	O6	-8.1(3)	N8	C1	C2	C11	137.68(18)
C1	C3	C6	O7	-120.65(18)	C2	C3	C6	O7	175.25(16)	N8	C1	C3	C2	110.21(19)
C2	C1	C3	C4	114.32(19)	C3	C1	C2	C11	-115.4(2)	N8	C1	C3	C4	-135.46(18)
C2	C1	C3	C6	-102.20(18)	C3	C1	N8	O9	62.9(2)	N8	C1	C3	C6	8.0(2)
C2	C1	N8	O9	133.6(2)	C3	C1	N8	O10	-119.9(2)	O6	C6	O7	C7	0.8(3)
C2	C1	N8	O10	-49.1(2)										

Table S8. Bond lengths for **9a**.

**Atom**	**Atom**	**Length/Å**	**Atom**	**Atom**	**Length/Å**	**Atom**	**Atom**	**Length/Å**
C1A	C2A	1.485(4)	C8A	N5A	1.416(4)	C11A	C12A	1.390(6)
C1A	C3A	1.524(4)	C8B	C9B	1.381(4)	C11B	C12B	1.396(5)
C1A	N16A	1.475(4)	C8B	C13B	1.396(5)	C12A	C13A	1.384(5)
C1B	C2B	1.487(4)	C8B	N5B	1.416(4)	C12B	C13B	1.380(5)
C1B	C3B	1.514(4)	C9A	C10A	1.391(5)	C19A	Cl1A	1.775(4)
C1B	N16B	1.471(4)	C9B	C10B	1.386(5)	C19A	Cl2A	1.785(4)
C2A	C3A	1.541(4)	C10A	C11A	1.381(5)	C19A	Cl3A	1.770(3)
C2A	C19A	1.520(5)	C10B	C11B	1.376(5)	C19B	Cl1B	1.770(3)
C2B	C3B	1.539(4)	C4B	N5B	1.373(4)	C19B	Cl2B	1.795(3)
C2B	C19B	1.518(5)	C4B	O15B	1.217(4)	C19B	Cl3B	1.778(3)
C3A	C4A	1.503(4)	C7A	C14A	1.493(5)	N5A	N6A	1.414(4)
C3A	C7A	1.491(5)	C7A	N6A	1.290(4)	N5B	N6B	1.413(4)
C3B	C4B	1.508(4)	C7B	C14B	1.488(5)	N16A	O17A	1.220(4)
C3B	C7B	1.501(4)	C7B	N6B	1.294(4)	N16A	O18A	1.228(4)
C4A	N5A	1.374(4)	C8A	C9A	1.394(5)	N16B	O17B	1.233(4)
C4A	O15A	1.216(4)	C8A	C13A	1.398(5)	N16B	O18B	1.223(4)

Table S9. Bond angles for **9a**.

**Atom**	**Atom**	**Atom**	**Angle/˚**	**Atom**	**Atom**	**Atom**	**Angle/˚**	**Atom**	**Atom**	**Atom**	**Angle/˚**
C2A	C1A	C3A	61.6(2)	C9B	C8B	C13B	119.9(3)	C2A	C19A	Cl1A	109.3(2)
N16A	C1A	C2A	119.0(3)	C9B	C8B	N5B	119.3(3)	C2A	C19A	Cl2A	107.6(2)
N16A	C1A	C3A	121.7(3)	C13B	C8B	N5B	120.7(3)	C2A	C19A	Cl3A	114.4(2)
C2B	C1B	C3B	61.7(2)	C10A	C9A	C8A	119.8(3)	Cl1A	C19A	Cl2A	108.84(19)
N16B	C1B	C2B	120.2(3)	C8B	C9B	C10B	119.6(3)	Cl3A	C19A	Cl1A	109.67(19)
N16B	C1B	C3B	121.1(3)	C11A	C10A	C9A	120.6(3)	Cl3A	C19A	Cl2A	106.82(18)
C1A	C2A	C3A	60.4(2)	C11B	C10B	C9B	121.4(3)	C2B	C19B	Cl1B	115.3(2)
C1A	C2A	C19A	123.4(3)	C10A	C11A	C12A	119.2(3)	C2B	C19B	Cl2B	108.7(2)
C19A	C2A	C3A	125.0(3)	C10B	C11B	C12B	118.4(3)	C2B	C19B	Cl3B	109.2(2)
C1B	C2B	C3B	60.0(2)	C13A	C12A	C11A	121.4(3)	Cl1B	C19B	Cl2B	106.33(17)
C1B	C2B	C19B	124.2(3)	C13B	C12B	C11B	121.1(3)	Cl1B	C19B	Cl3B	109.73(18)
C19B	C2B	C3B	123.0(3)	C12A	C13A	C8A	118.9(3)	Cl3B	C19B	Cl2B	107.18(17)
C1A	C3A	C2A	58.0(2)	C12B	C13B	C8B	119.4(3)	C4A	N5A	C8A	129.3(3)

Atom	Atom	Atom	Angle/˚	Atom	Atom	Atom	Angle/˚	Atom	Atom	Atom	Angle/˚
C4A	C3A	C1A	116.4(3)	O15A	C4A	N5A	127.6(3)	C4A	N5A	N6A	112.2(3)
C4A	C3A	C2A	121.2(3)	N5B	C4B	C3B	104.5(3)	N6A	N5A	C8A	118.4(3)
C7A	C3A	C1A	131.9(3)	O15B	C4B	C3B	127.6(3)	C4B	N5B	C8B	128.6(3)
C7A	C3A	C2A	120.9(3)	O15B	C4B	N5B	127.9(3)	C4B	N5B	N6B	112.2(3)
C7A	C3A	C4A	103.9(3)	C3A	C7A	C14A	129.3(3)	N6B	N5B	C8B	118.8(3)
C1B	C3B	C2B	58.3(2)	N6A	C7A	C3A	110.2(3)	C7A	N6A	N5A	109.2(3)
C4B	C3B	C1B	115.9(3)	N6A	C7A	C14A	120.4(3)	C7B	N6B	N5B	109.4(3)
C4B	C3B	C2B	120.5(3)	C14B	C7B	C3B	129.3(3)	O17A	N16A	C1A	120.1(3)
C7B	C3B	C1B	134.3(3)	N6B	C7B	C3B	110.0(3)	O17A	N16A	O18A	124.3(3)
C7B	C3B	C2B	119.6(3)	N6B	C7B	C14B	120.6(3)	O18A	N16A	C1A	115.5(3)
C7B	C3B	C4B	103.5(2)	C9A	C8A	C13A	120.1(3)	O17B	N16B	C1B	115.8(3)
N5A	C4A	C3A	104.2(3)	C9A	C8A	N5A	119.4(3)	O18B	N16B	C1B	119.8(3)
O15A	C4A	C3A	128.2(3)	C13A	C8A	N5A	120.6(3)	O18B	N16B	O17B	124.4(3)

Table S10. Torsion angles for **9a**.

A	B	C	D	Angle/˚	A	B	C	D	Angle/˚
C1A	C2A	C3A	C4A	103.5(3)	C4B	C3B	C7B	C14B	-178.4(3)
C1A	C2A	C3A	C7A	-123.0(3)	C4B	C3B	C7B	N6B	6.5(3)
C1A	C2A	C19A	Cl1A	-123.9(3)	C4B	N5B	N6B	C7B	-0.7(4)
C1A	C2A	C19A	Cl2A	118.1(3)	C7A	C3A	C4A	N5A	-5.5(3)
C1A	C2A	C19A	Cl3A	-0.5(4)	C7A	C3A	C4A	O15A	173.3(3)
C1A	C3A	C4A	N5A	-158.4(3)	C7B	C3B	C4B	N5B	-6.5(3)
C1A	C3A	C4A	O15A	20.3(5)	C7B	C3B	C4B	O15B	173.0(3)
C1A	C3A	C7A	C14A	-31.2(6)	C8A	C9A	C10A	C11A	-2.1(5)
C1A	C3A	C7A	N6A	152.0(3)	C8A	N5A	N6A	C7A	175.2(3)
C1B	C2B	C3B	C4B	103.4(3)	C8B	C9B	C10B	C11B	-2.1(6)
C1B	C2B	C3B	C7B	-126.4(3)	C8B	N5B	N6B	C7B	173.1(3)
C1B	C2B	C19B	Cl1B	1.6(4)	C9A	C8A	C13A	C12A	-1.6(5)
C1B	C2B	C19B	Cl2B	120.9(3)	C9A	C8A	N5A	C4A	-178.3(3)
C1B	C2B	C19B	Cl3B	-122.4(3)	C9A	C8A	N5A	N6A	6.1(4)
C1B	C3B	C4B	N5B	-162.8(3)	C9A	C10A	C11A	C12A	0.0(6)
C1B	C3B	C4B	O15B	16.7(5)	C9B	C8B	C13B	C12B	-2.8(5)
C1B	C3B	C7B	C14B	-28.8(6)	C9B	C8B	N5B	C4B	174.6(3)

A	B	C	D	Angle/˚	A	B	C	D	Angle/˚
C1B	C3B	C7B	N6B	156.1(3)	C9B	C8B	N5B	N6B	2.0(4)
C2A	C1A	C3A	C4A	-111.8(3)	C9B	C10B	C11B	C12B	-0.7(6)
C2A	C1A	C3A	C7A	104.6(4)	C10A	C11A	C12A	C13A	1.4(6)
C2A	C1A	N16A	O17A	-17.7(5)	C10B	C11B	C12B	C13B	1.8(6)
C2A	C1A	N16A	O18A	161.0(3)	C11A	C12A	C13A	C8A	-0.6(6)
C2A	C3A	C4A	N5A	134.6(3)	C11B	C12B	C13B	C8B	-0.1(6)
C2A	C3A	C4A	O15A	-46.6(5)	C13A	C8A	C9A	C10A	2.9(5)
C2A	C3A	C7A	C14A	41.7(5)	C13A	C8A	N5A	C4A	2.0(5)
C2A	C3A	C7A	N6A	-135.1(3)	C13A	C8A	N5A	N6A	-173.6(3)
C2B	C1B	C3B	C4B	-111.2(3)	C13B	C8B	C9B	C10B	3.9(5)
C2B	C1B	C3B	C7B	101.9(4)	C13B	C8B	N5B	C4B	-4.2(5)
C2B	C1B	N16B	O17B	154.1(3)	C13B	C8B	N5B	N6B	-176.8(3)
C2B	C1B	N16B	O18B	-25.9(5)	C14A	C7A	N6A	N5A	-179.8(3)
C2B	C3B	C4B	N5B	130.3(3)	C14B	C7B	N6B	N5B	-179.5(3)
C2B	C3B	C4B	O15B	-50.2(5)	C19A	C2A	C3A	C1A	-112.1(4)
C2B	C3B	C7B	C14B	44.3(5)	C19A	C2A	C3A	C4A	-8.5(5)
C2B	C3B	C7B	N6B	-130.8(3)	C19A	C2A	C3A	C7A	124.9(3)
C3A	C1A	C2A	C19A	114.6(3)	C19B	C2B	C3B	C1B	-113.6(3)
C3A	C1A	N16A	O17A	55.0(4)	C19B	C2B	C3B	C4B	-10.1(4)
C3A	C1A	N16A	O18A	-126.3(3)	C19B	C2B	C3B	C7B	120.0(3)
C3A	C2A	C19A	Cl1A	-48.9(4)	N5A	C8A	C9A	C10A	-176.8(3)
C3A	C2A	C19A	Cl2A	-166.9(2)	N5A	C8A	C13A	C12A	178.1(3)
C3A	C2A	C19A	Cl3A	74.5(4)	N5B	C8B	C9B	C10B	-174.9(3)
C3A	C4A	N5A	C8A	-171.6(3)	N5B	C8B	C13B	C12B	176.0(3)
C3A	C4A	N5A	N6A	4.3(3)	N16A	C1A	C2A	C3A	112.5(3)
C3A	C7A	N6A	N5A	-2.6(4)	N16A	C1A	C2A	C19A	-132.9(3)
C3B	C1B	C2B	C19B	111.6(3)	N16A	C1A	C3A	C2A	-108.3(3)
C3B	C1B	N16B	O17B	-132.7(3)	N16A	C1A	C3A	C4A	139.9(3)
C3B	C1B	N16B	O18B	47.3(4)	N16A	C1A	C3A	C7A	-3.8(5)
C3B	C2B	C19B	Cl1B	75.5(3)	N16B	C1B	C2B	C3B	111.4(3)
C3B	C2B	C19B	Cl2B	-165.3(2)	N16B	C1B	C2B	C19B	-136.9(3)
C3B	C2B	C19B	Cl3B	-48.6(4)	N16B	C1B	C3B	C2B	-110.1(3)
C3B	C4B	N5B	C8B	-168.2(3)	N16B	C1B	C3B	C4B	138.7(3)
C3B	C4B	N5B	N6B	4.8(3)	N16B	C1B	C3B	C7B	-8.1(5)

A	B	C	D	Angle/˚	A	B	C	D	Angle/˚
C3B	C7B	N6B	N5B	-3.9(4)	O15A	C4A	N5A	C8A	9.7(6)
C4A	C3A	C7A	C14A	-178.1(3)	O15A	C4A	N5A	N6A	-174.4(3)
C4A	C3A	C7A	N6A	5.1(4)	O15B	C4B	N5B	C8B	12.3(5)
C4A	N5A	N6A	C7A	-1.2(4)	O15B	C4B	N5B	N6B	-174.7(3)

Table S11. Bond lengths for **9b**.

Atom	Atom	Length/Å	Atom	Atom	Length/Å	Atom	Atom	Length/Å
C1A	C2A	1.468(2)	C8A	N5A	1.419(2)	C11A	C12A	1.390(3)
C1A	C3A	1.517(2)	C8B	C9B	1.391(3)	C11B	C12B	1.388(3)
C1A	N16A	1.491(2)	C8B	C13B	1.399(2)	C12A	C13A	1.389(3)
C1B	C2B	1.470(2)	C8B	N5B	1.421(2)	C12B	C13B	1.388(3)
C1B	C3B	1.525(2)	C9A	C10A	1.387(3)	C19	Cl1B	1.7718(18)
C1B	N16B	1.485(2)	C9B	C10B	1.389(3)	C19	Cl2B	1.7615(18)
C2A	C3A	1.538(2)	C10A	C11A	1.386(3)	C19	Cl3B	1.7850(18)
C2A	C19A	1.517(2)	C10B	C11B	1.390(3)	C19A	Cl1A	1.7841(19)
C2B	C3B	1.540(2)	C4B	N5B	1.368(2)	C19A	Cl2A	1.7653(19)
C2B	C19	1.512(2)	C4B	O15B	1.216(2)	C19A	Cl3A	1.7681(18)
C3A	C4A	1.503(2)	C7A	C14A	1.488(2)	N5A	N6A	1.407(2)
C3A	C7A	1.487(2)	C7A	N6A	1.287(2)	N5B	N6B	1.4085(19)
C3B	C4B	1.506(2)	C7B	C14B	1.487(2)	N16A	O17A	1.219(2)
C3B	C7B	1.487(2)	C7B	N6B	1.286(2)	N16A	O18A	1.222(2)
C4A	N5A	1.367(2)	C8A	C9A	1.394(3)	N16B	O17B	1.220(2)
C4A	O15A	1.215(2)	C8A	C13A	1.392(3)	N16B	O18B	1.219(2)

Table S12. Bond angles for **9b**.

Atom	Atom	Atom	Angle/˚	Atom	Atom	Atom	Angle/˚	Atom	Atom	Atom	Angle/˚
C2A	C1A	C3A	62.00(11)	C9B	C8B	C13B	120.46(16)	C2B	C19	Cl1B	113.93(13)
C2A	C1A	N16A	118.15(15)	C9B	C8B	N5B	120.67(15)	C2B	C19	Cl2B	110.64(12)
N16A	C1A	C3A	119.60(14)	C13B	C8B	N5B	118.88(15)	C2B	C19	Cl3B	106.23(12)
C2B	C1B	C3B	61.88(11)	C10A	C9A	C8A	118.84(19)	Cl1B	C19	Cl3B	107.19(9)
C2B	C1B	N16B	117.98(15)	C10B	C9B	C8B	119.25(17)	Cl2B	C19	Cl1B	108.55(10)
N16B	C1B	C3B	118.89(14)	C11A	C10A	C9A	120.92(19)	Cl2B	C19	Cl3B	110.21(10)

Atom	Atom	Atom	Angle/˚	Atom	Atom	Atom	Angle/˚	Atom	Atom	Atom	Angle/˚
C1A	C2A	C3A	60.55(11)	C9B	C10B	C11B	120.88(18)	C2A	C19A	Cl1A	106.72(12)
C1A	C2A	C19A	123.31(15)	C10A	C11A	C12A	119.54(18)	C2A	C19A	Cl2A	110.20(12)
C19A	C2A	C3A	127.45(15)	C12B	C11B	C10B	119.33(17)	C2A	C19A	Cl3A	113.97(13)
C1B	C2B	C3B	60.82(11)	C13A	C12A	C11A	120.6(2)	Cl2A	C19A	Cl1A	109.31(10)
C1B	C2B	C19	122.65(15)	C13B	C12B	C11B	120.74(17)	Cl2A	C19A	Cl3A	108.55(10)
C19	C2B	C3B	128.67(15)	C12A	C13A	C8A	119.02(18)	Cl3A	C19A	Cl1A	108.00(9)
C1A	C3A	C2A	57.44(11)	C12B	C13B	C8B	119.31(17)	C4A	N5A	C8A	127.74(15)
C4A	C3A	C1A	120.47(14)	O15A	C4A	N5A	128.54(16)	C4A	N5A	N6A	112.55(14)
C4A	C3A	C2A	113.47(14)	N5B	C4B	C3B	104.49(14)	N6A	N5A	C8A	119.54(14)
C7A	C3A	C1A	126.51(15)	O15B	C4B	C3B	126.79(16)	C4B	N5B	C8B	128.49(14)
C7A	C3A	C2A	131.16(15)	O15B	C4B	N5B	128.71(16)	C4B	N5B	N6B	112.30(13)
C7A	C3A	C4A	103.30(14)	C3A	C7A	C14A	128.18(16)	N6B	N5B	C8B	119.14(13)
C1B	C3B	C2B	57.29(11)	N6A	C7A	C3A	110.94(15)	C7A	N6A	N5A	108.70(14)
C4B	C3B	C1B	118.30(14)	N6A	C7A	C14A	120.82(16)	C7B	N6B	N5B	109.05(14)
C4B	C3B	C2B	114.14(14)	C14B	C7B	C3B	128.46(16)	O17A	N16A	C1A	116.01(15)
C7B	C3B	C1B	126.21(15)	N6B	C7B	C3B	110.78(15)	O17A	N16A	O18A	125.21(16)
C7B	C3B	C2B	132.83(15)	N6B	C7B	C14B	120.73(16)	O18A	N16A	C1A	118.75(15)
C7B	C3B	C4B	103.36(14)	C9A	C8A	N5A	118.71(16)	O17B	N16B	C1B	115.87(16)
N5A	C4A	C3A	104.49(14)	C13A	C8A	C9A	121.04(17)	O18B	N16B	C1B	119.07(15)
O15A	C4A	C3A	126.95(15)	C13A	C8A	N5A	120.23(16)	O18B	N16B	O17B	125.03(17)

Table S13. Torsion angles for **9b**.

A	B	C	D	Angle/˚	A	B	C	D	Angle/˚
C1A	C2A	C3A	C4A	112.25(16)	C4B	C3B	C7B	C14B	178.34(18)
C1A	C2A	C3A	C7A	-112.3(2)	C4B	C3B	C7B	N6B	-0.03(19)
C1A	C2A	C19A	Cl1A	-111.33(16)	C4B	N5B	N6B	C7B	-0.9(2)
C1A	C2A	C19A	Cl2A	130.09(15)	C7A	C3A	C4A	N5A	-1.30(18)
C1A	C2A	C19A	Cl3A	7.8(2)	C7A	C3A	C4A	O15A	-179.71(18)
C1A	C3A	C4A	N5A	-149.33(16)	C7B	C3B	C4B	N5B	-0.51(18)
C1A	C3A	C4A	O15A	32.3(3)	C7B	C3B	C4B	O15B	179.01(18)
C1A	C3A	C7A	C14A	-30.3(3)	C8A	C9A	C10A	C11A	-0.4(3)
C1A	C3A	C7A	N6A	147.02(17)	C8A	N5A	N6A	C7A	175.92(16)
C1B	C2B	C3B	C4B	-109.25(16)	C8B	C9B	C10B	C11B	-0.8(3)

A	B	C	D	Angle/˚	A	B	C	D	Angle/˚
C1B	C2B	C3B	C7B	111.2(2)	C8B	N5B	N6B	C7B	-178.32(15)
C1B	C2B	C19	Cl1B	-13.2(2)	C9A	C8A	C13A	C12A	-1.3(3)
C1B	C2B	C19	Cl2B	-135.86(14)	C9A	C8A	N5A	C4A	155.42(18)
C1B	C2B	C19	Cl3B	104.53(16)	C9A	C8A	N5A	N6A	-19.4(2)
C1B	C3B	C4B	N5B	144.33(15)	C9A	C10A	C11A	C12A	-0.4(3)
C1B	C3B	C4B	O15B	-36.2(3)	C9B	C8B	C13B	C12B	-1.4(3)
C1B	C3B	C7B	C14B	37.3(3)	C9B	C8B	N5B	C4B	8.6(3)
C1B	C3B	C7B	N6B	-141.10(17)	C9B	C8B	N5B	N6B	-174.52(16)
C2A	C1A	C3A	C4A	-99.94(17)	C9B	C10B	C11B	C12B	-0.4(3)
C2A	C1A	C3A	C7A	119.9(2)	C10A	C11A	C12A	C13A	0.4(3)
C2A	C1A	N16A	O17A	163.16(15)	C10B	C11B	C12B	C13B	0.6(3)
C2A	C1A	N16A	O18A	-18.8(2)	C11A	C12A	C13A	C8A	0.5(3)
C2A	C3A	C4A	N5A	145.84(15)	C11B	C12B	C13B	C8B	0.2(3)
C2A	C3A	C4A	O15A	-32.6(3)	C13A	C8A	C9A	C10A	1.2(3)
C2A	C3A	C7A	C14A	45.7(3)	C13A	C8A	N5A	C4A	-23.4(3)
C2A	C3A	C7A	N6A	-137.00(19)	C13A	C8A	N5A	N6A	161.79(16)
C2B	C1B	C3B	C4B	101.90(17)	C13B	C8B	C9B	C10B	1.7(3)
C2B	C1B	C3B	C7B	-122.1(2)	C13B	C8B	N5B	C4B	-171.10(17)
C2B	C1B	N16B	O17B	-161.00(16)	C13B	C8B	N5B	N6B	5.8(2)
C2B	C1B	N16B	O18B	20.8(2)	C14A	C7A	N6A	N5A	176.32(17)
C2B	C3B	C4B	N5B	-151.21(14)	C14B	C7B	N6B	N5B	-177.96(16)
C2B	C3B	C4B	O15B	28.3(3)	C19	C2B	C3B	C1B	-110.2(2)
C2B	C3B	C7B	C14B	-39.2(3)	C19	C2B	C3B	C4B	140.59(18)
C2B	C3B	C7B	N6B	142.46(19)	C19	C2B	C3B	C7B	1.1(3)
C3A	C1A	C2A	C19A	-117.68(18)	C19A	C2A	C3A	C1A	111.2(2)
C3A	C1A	N16A	O17A	91.1(2)	C19A	C2A	C3A	C4A	-136.53(17)
C3A	C1A	N16A	O18A	-90.89(19)	C19A	C2A	C3A	C7A	-1.1(3)
C3A	C2A	C19A	Cl1A	172.42(14)	N5A	C8A	C9A	C10A	-177.56(17)
C3A	C2A	C19A	Cl2A	53.8(2)	N5A	C8A	C13A	C12A	177.49(17)
C3A	C2A	C19A	Cl3A	-68.5(2)	N5B	C8B	C9B	C10B	-177.96(17)
C3A	C4A	N5A	C8A	-174.45(16)	N5B	C8B	C13B	C12B	178.24(16)
C3A	C4A	N5A	N6A	0.68(19)	N16A	C1A	C2A	C3A	-110.49(16)
C3A	C7A	N6A	N5A	-1.3(2)	N16A	C1A	C2A	C19A	131.83(17)
C3B	C1B	C2B	C19	119.48(18)	N16A	C1A	C3A	C2A	108.21(18)

A	B	C	D	Angle/˚	A	B	C	D	Angle/˚
C3B	C1B	N16B	O17B	-89.4(2)	N16A	C1A	C3A	C4A	8.3(2)
C3B	C1B	N16B	O18B	92.38(19)	N16A	C1A	C3A	C7A	-131.86(18)
C3B	C2B	C19	Cl1B	63.5(2)	N16B	C1B	C2B	C3B	109.65(17)
C3B	C2B	C19	Cl2B	-59.1(2)	N16B	C1B	C2B	C19	-130.86(17)
C3B	C2B	C19	Cl3B	-178.70(15)	N16B	C1B	C3B	C2B	-108.23(18)
C3B	C4B	N5B	C8B	177.95(16)	N16B	C1B	C3B	C4B	-6.3(2)
C3B	C4B	N5B	N6B	0.87(19)	N16B	C1B	C3B	C7B	129.69(19)
C3B	C7B	N6B	N5B	0.6(2)	O15A	C4A	N5A	C8A	3.9(3)
C4A	C3A	C7A	C14A	-175.74(19)	O15A	C4A	N5A	N6A	179.05(18)
C4A	C3A	C7A	N6A	1.6(2)	O15B	C4B	N5B	C8B	-1.6(3)
C4A	N5A	N6A	C7A	0.3(2)	O15B	C4B	N5B	N6B	-178.63(18)

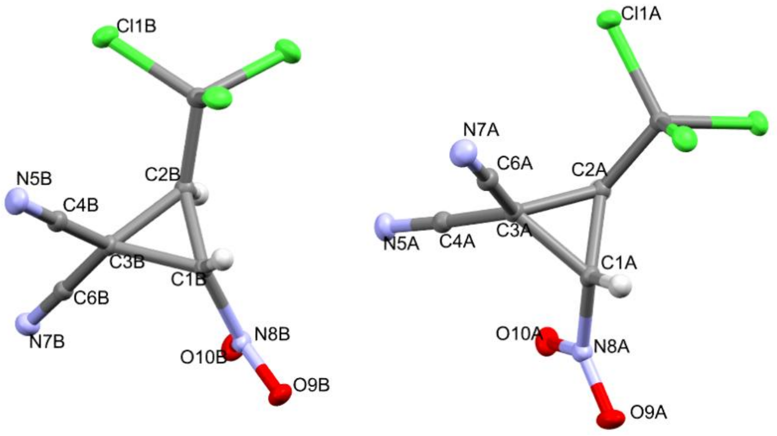

Figure S47. Geometry of 2 independent molecules (A and B) **2** in crystal. Ellipsoids of anisotropic displacements are shown with 50% probability.

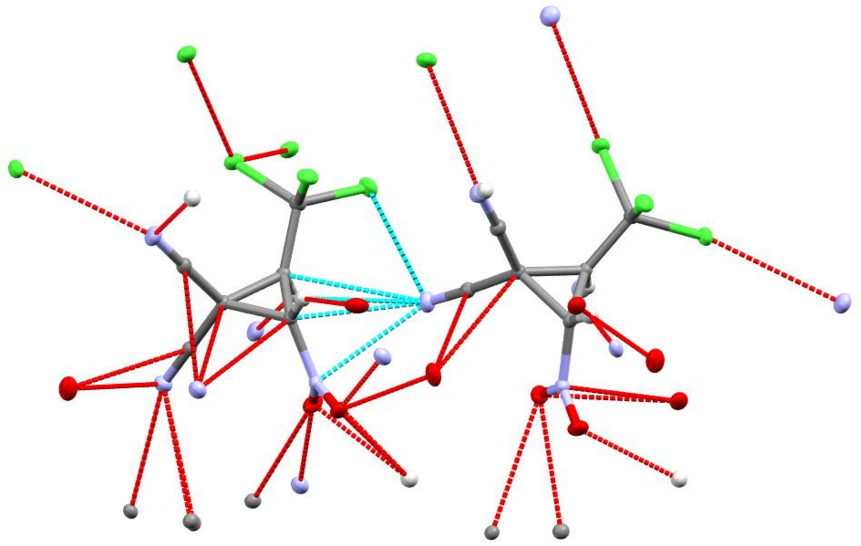

Figure S48. System of short contacts in crystal **2**. Contacts are shown with dotted lines.

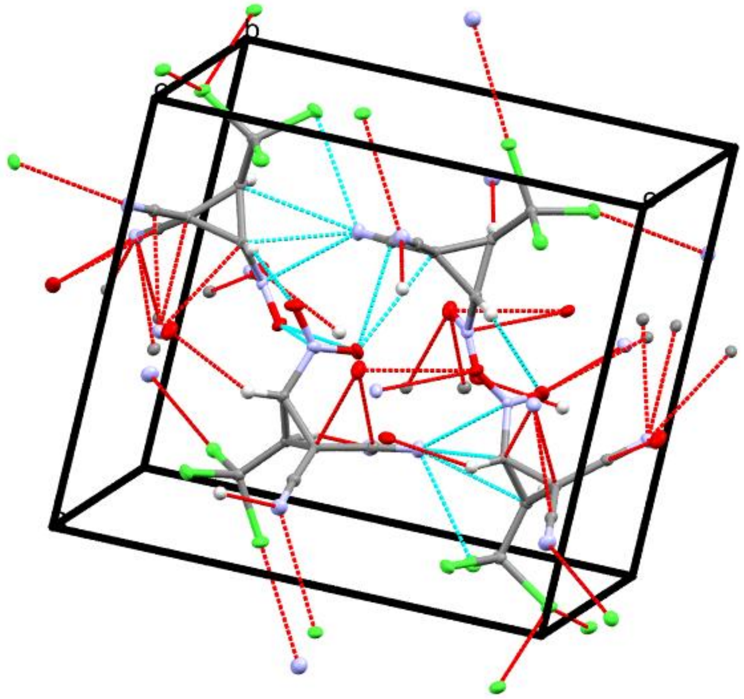

Figure S49. Crystal packaging of **2**. Short contacts are shown with dotted lines.

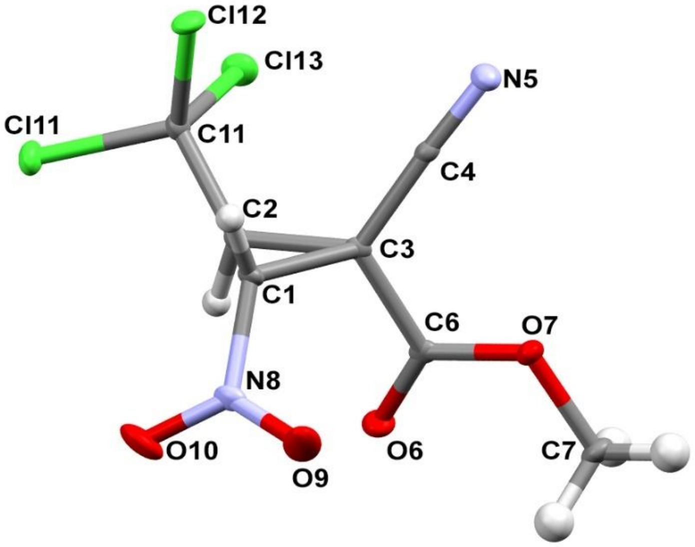

Figure S50. Geometry of 2 independent molecules (A and B) **3** in crystal.

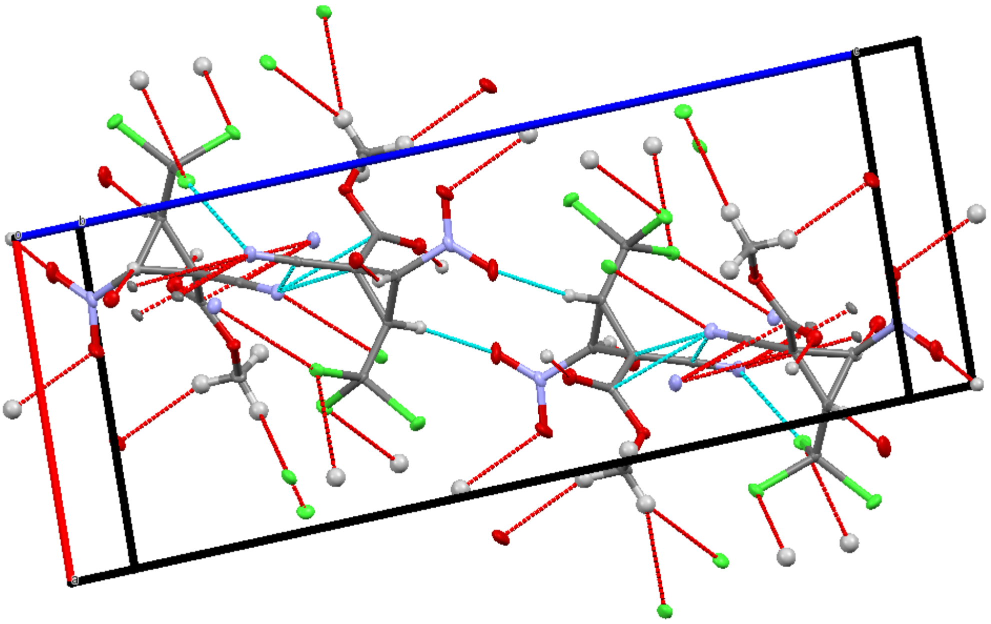

Figure S51. Crystal packaging of **3**. Short contacts are shown with dotted lines.

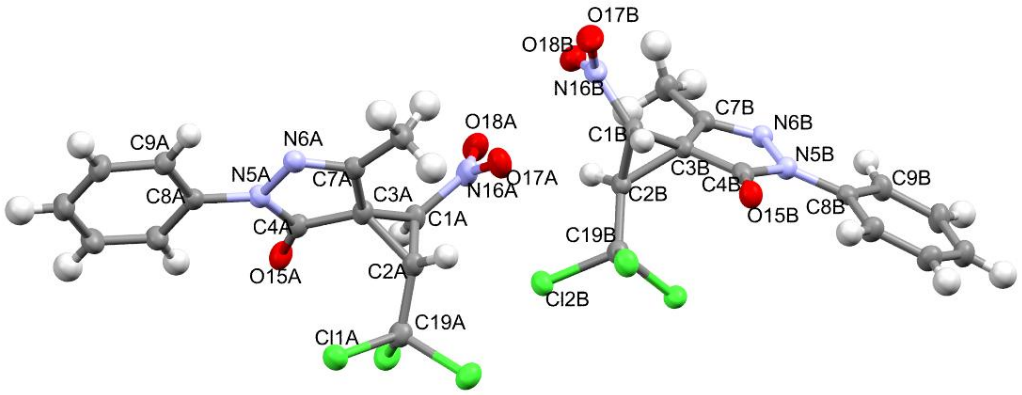

Figure S52. Geometry of 2 independent molecules **9a** in crystal. Ellipsoids of anisotropic displacements are shown with 50% probability.

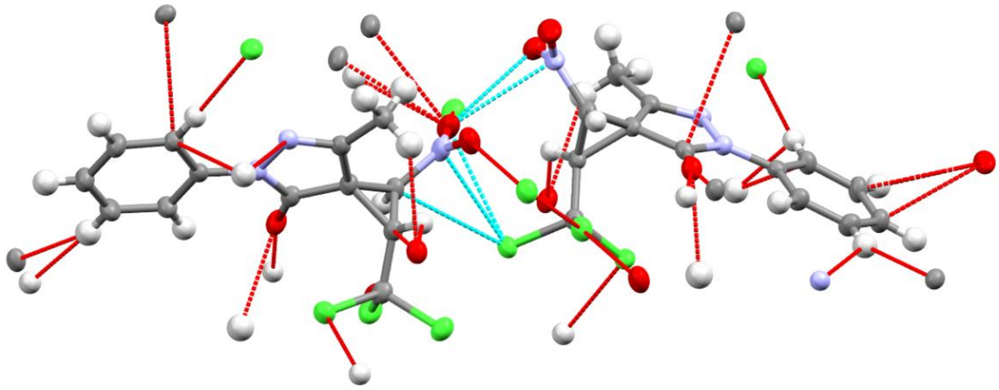

Figure S53. System of short contacts in crystal **9a**. Short contacts are shown with dotted lines.

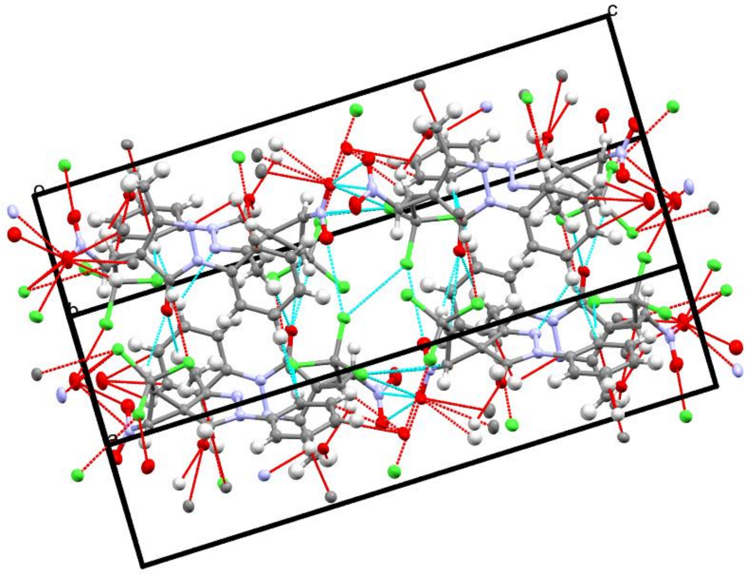

Figure S54. Fragment of **9a** crystal packaging. Short contacts are shown with dotted lines.

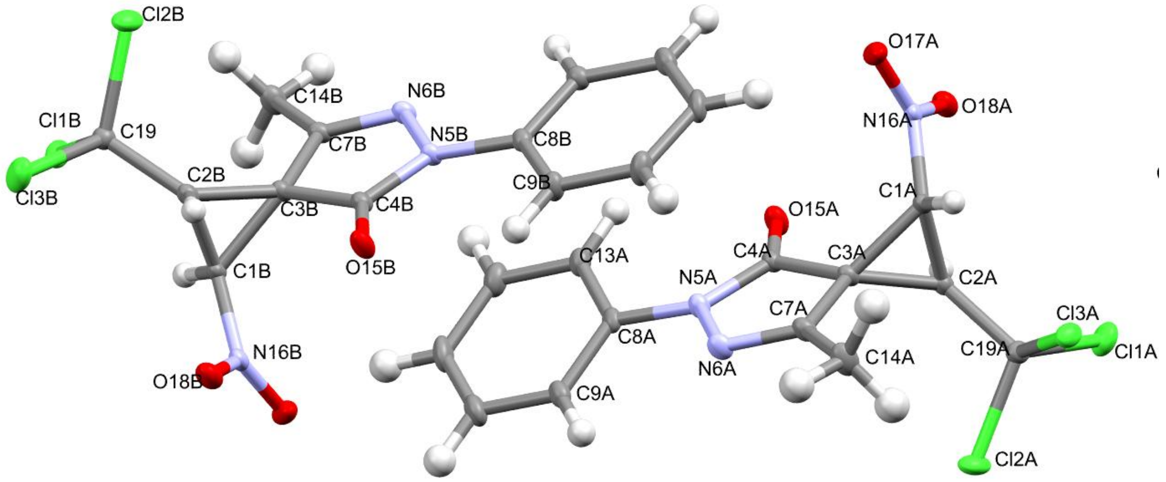

Figure S55. Geometry of 2 independent molecules **9b** in crystal. Ellipsoids of anisotropic displacements are shown with 50% probability.

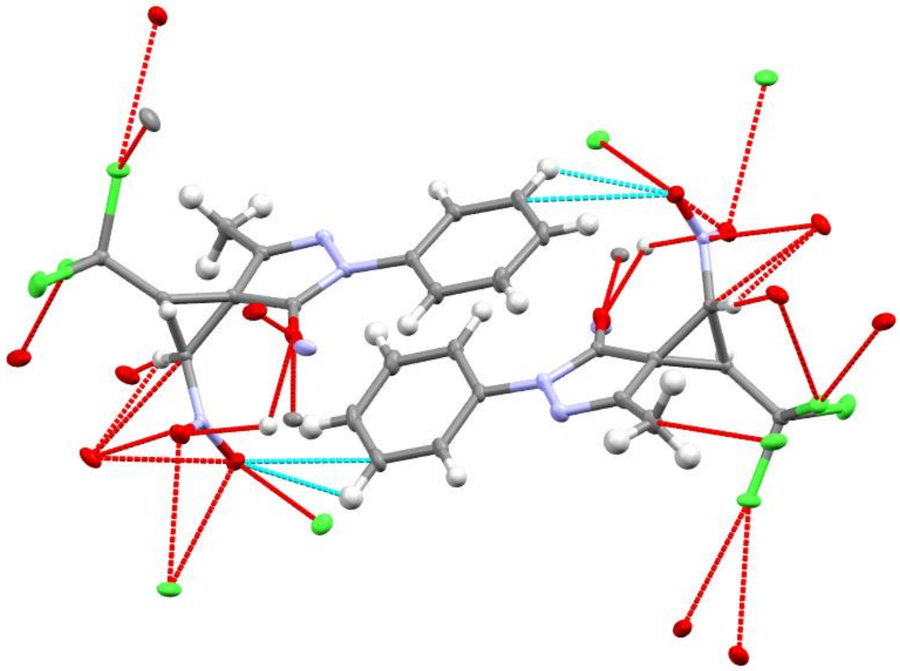

Figure S56. System of short contacts in crystal **9b**. Short contacts are shown with dotted lines.

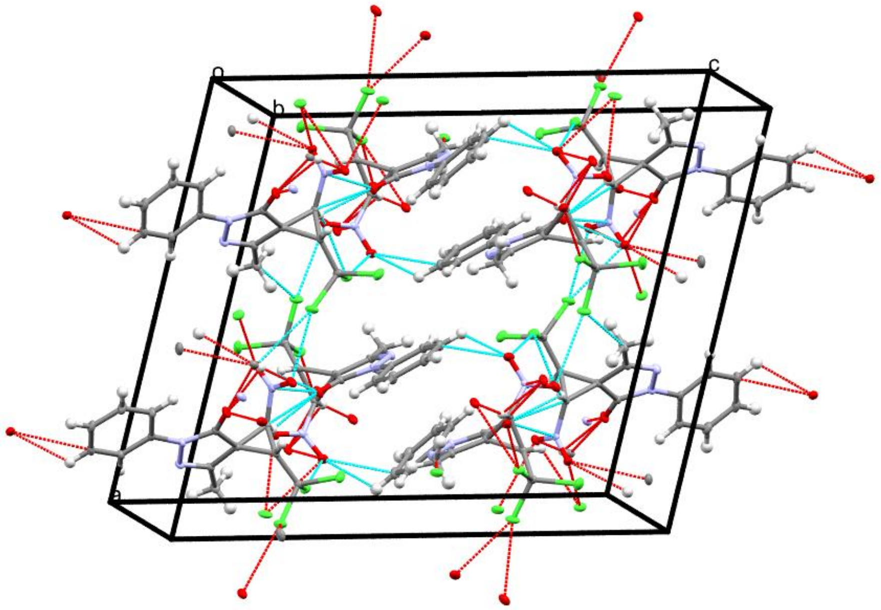

Figure S57. Fragment of **9b** crystal packaging. Short contacts are shown with dotted lines.

References

1. Sheldrick, G.M. *Acta Crystallogr. Sect. A Found. Adv*., **2015**, 71, 3-8.

2. Sheldrick, G.M. *Acta Crystallogr. Sect*. *C,* **2015**, 71, 3-8.

3. Farrugia, L.J. *J. Appl. Crystallogr*., **2012**, 45, 849-854.

4. Spek, A.L. *Acta Crystallogr. Sect. D Biol. Crystallogr*., **2009**, 65, 148-155.

5. Durden, Jr, J. A., Heywood, D. L., Sousa, A. A., Spurr, H. W. *J. Agric. Food. Chem.* **1970**, 18 (1), 50-56. DOI: 10.1021/jf60167a011.
